# Targeted Penetrating Motif Engineering of BH3 Mimetic: Harnessing Non‐Canonical Amino Acids for Coinhibition of MCL‐1 and BCL‐xL in Acute Myeloid Leukemia

**DOI:** 10.1002/advs.202503682

**Published:** 2025-04-30

**Authors:** Zhe Wang, Ruizhi Lai, Xinpei Wang, Xu Chen, Youjian Zhou, Shengbin Li, Xiaohui Qiu, Zekai Zeng, Jianye Yuan, Jinghuan Mao, Zhidong Chen, Junqing Wang

**Affiliations:** ^1^ Department of Pathology The Eighth Affiliated Hospital Sun Yat‐sen University Shenzhen 518033 China; ^2^ School of Pharmaceutical Sciences Shenzhen Campus of Sun Yat‐sen University Shenzhen 518107 China

**Keywords:** acute myeloid leukemia, drug resistance, MCL‐1 & BCL‐xL inhibition, non‐canonical amino acids, targeted penetrating peptides

## Abstract

Acute Myeloid Leukemia (AML) remains a formidable clinical challenge, predominantly due to the emergence of resistance to existing therapeutic regimens, including BCL‐2 inhibitors like Venetoclax. Here, a novel approach is introduced by engineering BH3 mimetics utilizing non‐canonical amino acids (ncAAs) to achieve dual inhibition of MCL‐1 and BCL‐xL. Through site saturation mutagenesis scanning, the I58(Chg) mutation is identified, significantly enhancing binding affinity with IC_50_ values of 2.77 nm for MCL‐1 and 10.69 nm for BCL‐xL, reflecting an increase of fourfold or more. The developed vMIP‐II‐TAT‐I peptide, incorporating a CXCR4‐targeted penetrating motif, demonstrated superior cellular uptake, with mean fluorescence intensity (MFI) 7.2‐fold higher in CXCR4‐positive AML cells and exhibited a high selectivity index (SI) for AML cells, with minimal impact on normal human hematopoietic stem cells (HSCs). When combined with Venetoclax, this peptide induced synergistic apoptosis, reducing tumor burden and prolonging survival in an AML mouse model, with median survival extended to 53 days from 37 days with Venetoclax alone. These findings reveal the therapeutic potential of dual inhibition in overcoming Venetoclax resistance and selectively targeting leukemic cells with reduced off‐target effects, while laying the foundation for developing advanced BH3 mimetics with enhanced targeting, binding affinity, and efficacy for AML treatment.

## Graphical abstract

A structure‐guided peptide derived from a non‐canonical amino acid library simultaneously targets MCL‐1 and BCL‐xL to overcome apoptotic resistance in acute myeloid leukemia. Fused with a CXCR4‐binding motif, the engineered peptide exhibits selective intracellular delivery and potent in vivo efficacy, offering a promising therapeutic strategy against drug‐resistant hematological malignancies.

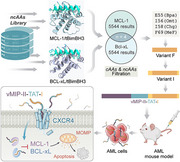

## 1 Introduction

AML is an aggressive hematologic malignancy marked by abnormal myeloid cell proliferation in the bone marrow (BM) and peripheral blood, accounting for ≈80% of adult leukemia cases.^[^ ^1^ ^]^ Despite intensive chemotherapy and subsequent allogeneic stem cell transplantation, the prognosis remains poor, with a 5‐year survival rate of ≈30%, improving slightly in younger patients.^[^ ^2^ ^]^ Recurrence due to unresolved resistance mechanisms poses a major challenge.^[^ ^3^ ^]^ The pathogenesis of AML involves genetic and epigenetic alterations that disrupt key pathways in differentiation, proliferation, and apoptosis, contributing to its heterogeneity and drug resistance.^[^ ^4^ ^]^ Traditional chemotherapies target rapidly dividing cells but fail to effectively eliminate leukemic stem cells, leading to persistent disease and relapse.^[^ ^5^ ^]^

Recent advancements in AML treatment have identified B‐cell lymphoma 2 (BCL‐2) as a key anti‐apoptotic protein essential for AML cell survival by inhibiting intrinsic apoptosis.^[^ ^6^ ^]^ BCL‐2 inhibition targets oxidative phosphorylation, selectively eliminating quiescent leukemia stem cells (LSCs) with low oxidative states (ROS‐low) and BCL‐2 overexpression.^[^ ^7^ ^]^ These LSCs, resistant to conventional chemotherapy, can be effectively targeted by BCL‐2 inhibitors, reducing leukemia burden and improving treatment outcomes. Venetoclax (ABT‐199), a BCL‐2 inhibitor, has shown promising clinical activity, particularly in combination with demethylating agents or low‐dose cytarabine, leading to higher response rates and improved survival in elderly AML patients.^[^ ^8^ ^]^ Other inhibitors, including Navitoclax (ABT‐263),^[^ ^9^ ^]^ Obatoclax (GX15‐070), and Oblimersen sodium (G3139), further support the potential of targeting BCL‐2 to address the unique metabolic dependencies of AML and improve outcomes.^[^ ^10^ ^]^ However, clinical efficacy is often limited by resistance mechanisms, including upregulation of alternative anti‐apoptotic proteins such as MCL‐1 and BCL‐xL, which can compensate for BCL‐2 inhibition and sustain leukemic cell survival and proliferation.^[^ ^11^ ^]^ This adaptive resistance necessitates the development of therapeutic strategies capable of simultaneously targeting multiple anti‐apoptotic pathways.

The dual inhibition of MCL‐1 and BCL‐xL has emerged as a promising strategy to overcome resistance to BCL‐2 inhibitors.^[^ ^12^ ^]^ Recent studies have demonstrated that small‐molecule dual inhibitors, which concurrently target both MCL‐1 and BCL‐2/BCL‐xL proteins, have IC_50_ values in the nanomolar to micromolar range, thereby significantly enhancing apoptotic responses.^[^ ^13^ ^]^ An alternative strategy involves the development of BH3 peptidomimetics, which are designed to emulate the natural binders more closely.^[^ ^14^ ^]^ By modifying peptides that correspond to the α‐helical BH3 domains found within recognized BCL‐2 family members, these peptide inhibitors can achieve higher affinity and specificity than their small‐molecule counterparts, attributable to their expansive interaction surfaces.^[^ ^15^ ^]^ The possibility of identifying peptides with the requisite properties can be enhanced through the screening of expanded libraries of ncAAs.^[^ ^16^ ^]^ In silico scanning of ncAAs within peptides offers substantial advantages, including the optimization of intermolecular interactions to enhance binding affinity and the prediction and modulation of peptide characteristics to boost cell permeability and in vivo stability. The vast chemical space accessible through modifications such as D‐amino acids, N‐methyl amino acids, artificial side‐chain amino acids, and others underscores the potential for extensive peptide diversification.

**Figure 1:**
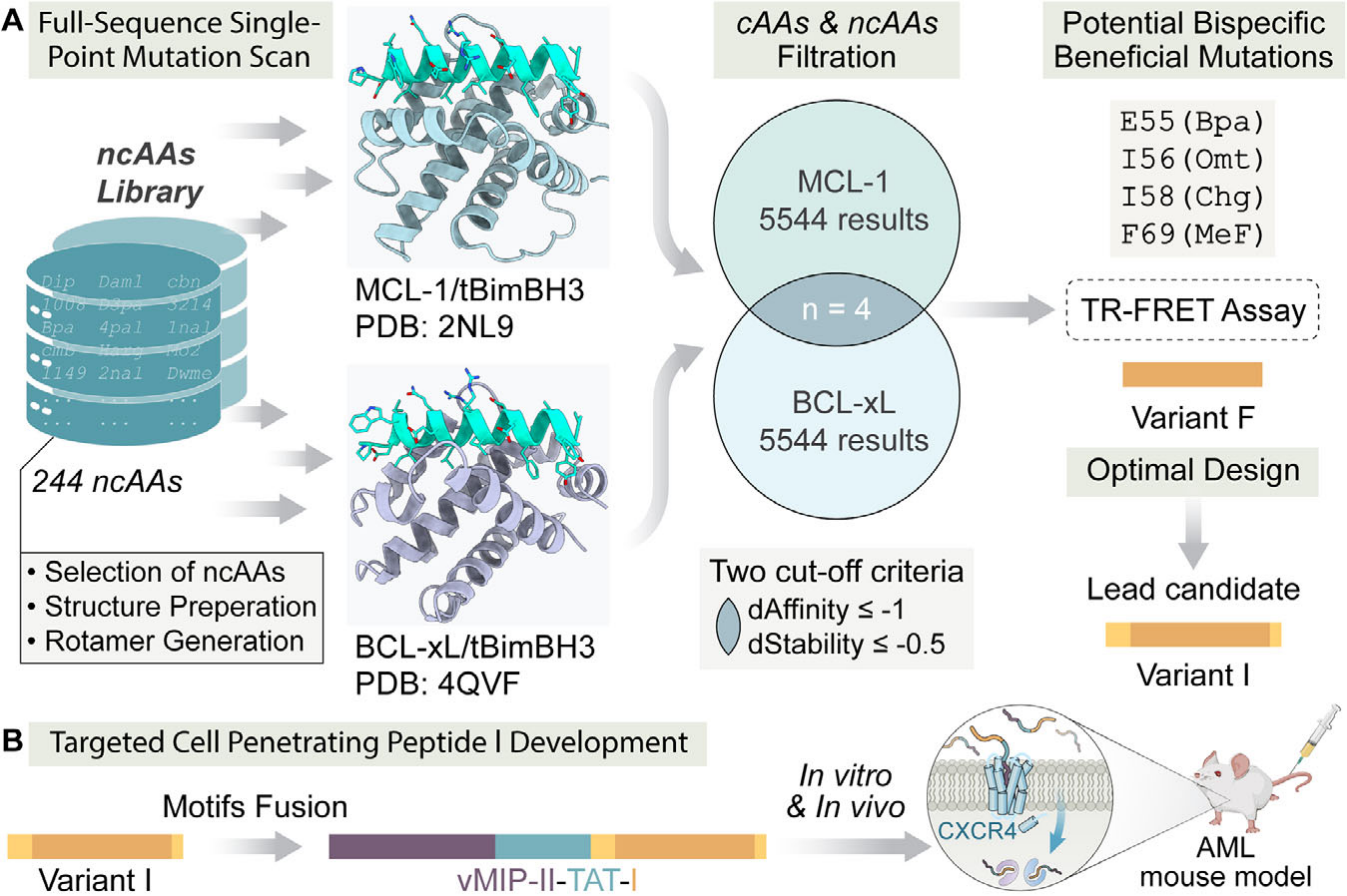
A workflow for developing bispecific BH3 peptidomimetics composed of beneficial mutations and CXCR4 selective cell‐penetrating motifs. A) It begins with a comprehensive library of 244 non‐canonical amino acids (ncAAs), undergoing selection, structure preparation, and rotamer generation. The structures of MCL‐1/tBimBH3 (PDB: 2NL9) and BCL‐xL/tBimBH3 (PDB: 4QVF) are shown.^[^ ^22^ ^]^ A filtration process results in 5,544 possible mutations for both MCL‐1 and BCL‐xL, with an overlap of four significant mutations based on the two cut‐off criteria. Identified mutation (E55 (Bpa), I56 (Omt), I58(Chg), F69 (MeF)) combinations are validated using a TR‐FRET assay, leading to the refinement of the F variant and design of a lead candidate I. B) The functionalization of variant I through motifs fusion results in a targeted cell‐penetrating peptide (vMIP‐II‐TAT‐I), which is further studied in vitro and in vivo in an AML mouse model.

In this study, we attempted to engineer targeted penetrating motifs within BH3 mimetics, focusing on the exploration of 20 canonical and 244 ncAAs for the dual inhibition of MCL‐1 and BCL‐xL through site saturation mutagenesis scanning at every position within the peptide (**Figure** **1**A). The lead peptide motifs were further engineered to possess targeting capabilities, enhanced cell penetration, and reduced off‐target effects, thereby reinforcing the consideration of peptidomimetics as potential therapeutic agents. CXCR4 is a key therapeutic target in AML due to its roles in cell survival, migration, chemoresistance, and BM microenvironment interaction.^[^ ^17^ ^]^ High CXCR4 expression correlates with poor prognosis,^[^ ^18^ ^]^ and its inhibition can disrupt the protective niche, mobilize leukemic cells for enhanced chemotherapy, and directly induce cytotoxicity, potentially reducing relapse.^[^ ^19^ ^]^ A CXCR4‐binding peptide derived from vMIP‐II (LGASWHRPDKCCLGYQKRPLP) can selectively deliver BH3 peptidomimetics to CXCR4‐expressing cells,^[^ ^20^ ^]^ while a TAT‐derived nonapeptide (RKKRRQRRR) enhances cellular internalization of these agents.^[^ ^21^ ^]^ By employing an in‐silico mutagenesis‐guided modular fusion design, we propose a dual targeting penetrating BH3‐mimetic, namely, vMIP‐II‐TAT‐I (Figure 1B), which is designed to circumvent the challenges of cellular impermeability and limited specificity for certain cell types that are commonly encountered with conventional BH3 mimetics. Moreover, it seeks to improve concurrent binding affinity to both MCL‐1 and BCL‐xL, addressing the persistent challenge of drug resistance in AML.

## 2 Results and Discussion

### 2.1 Enhancing Peptide‐Protein Interactions with Potential ncAAs and Design Optimization of Peptide Variants Against MCL‐1 and BCL‐xL

**Figure 2:**
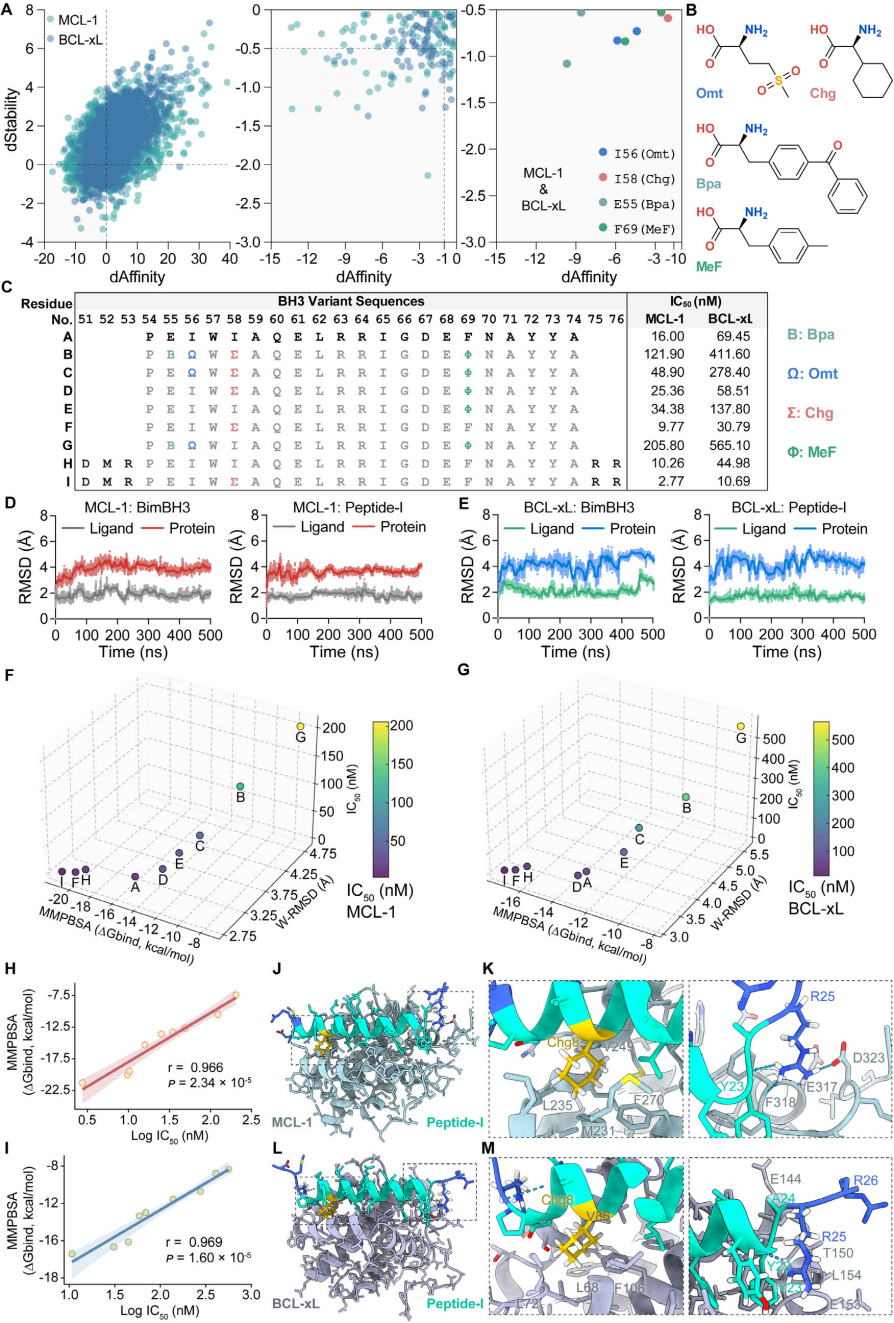
NcAAs‐Driven Optimization of BH3 Mimetic Binding Affinity, Stability, and Structural Dynamics for MCL‐1 and BCL‐xL Dual Inhibition. A) Saturation virtual mutagenesis of tBimBH3 (54–74) using 244 ncAAs identified four mutations that enhance binding to MCL‐1 and BCL‐xL. The second plot presents single specificity results, while the third highlights mutations exhibiting dual specificity for both targets. dStability values (kcal mol^−1^), representing the difference in Boltzmann‐weighted averages, indicate relative thermostability, with more negative values denoting greater structural stability. B) Chemical structures of the four selected ncAAs (Omt, Chg, MeF, and Bpa) were identified from the screening process. C) Binding affinities (IC_50_ values) of tBimBH3 (Peptide‐A) variants (B–I) were determined using a cell‐free TR‐FRET assay, assessing inhibitory activity against MCL‐1 and BCL‐xL. D–E) Root Mean Square Deviation (RMSD) trajectories over 500 ns molecular dynamics (MD) simulations, evaluating stability and conformational dynamics of MCL‐1 and BCL‐xL in complex with BimBH3(Peptide‐H) and Peptide‐I. F–G) 3D plots of binding free energy (ΔGbind, Molecular Mechanics Poisson–Boltzmann Surface Area (MMPBSA) method), Weighted RMSD (W‐RMSD), and IC₅₀ values for different peptide variants targeting MCL‐1 and BCL‐xL. H–I) Pearson correlation analysis between binding free energy (ΔGbind, MMPBSA) and IC_50_ values, showing notable correlations in both cases of MCL‐1 (red) and BCL‐xL (blue). Each data point represents the mean of four replicates, and shaded areas denote 95% confidence intervals. Correlation coefficients (r) and two‐tailed *p* values were calculated using Pearson's method, with *p* < 0.05 considered statistically significant. J–M) Structural representations of Peptide‐I bound to MCL‐1 and BCL‐xL.

Initially, a parameterized library comprising 244 commercially available ncAAs was constructed in the Molecular Operating Environment (MOE).^[^ ^23^ ^]^ To identify novel amino acid side chains that enhance peptide interactions with target proteins, we conducted single‐site saturation mutagenesis scanning. This involved sequentially screening 20 canonical amino acids (cAAs) and 244 ncAAs from our library at each residue position (residues 54–74) using the Residue Scan module in MOE (Figure S1, Supporting Information). The high‐resolution crystal structures of the truncated BimBH3 (tBimBH3) domain in complex with MCL‐1 (PDB: 2NL9) and BCL‐xL (PDB: 4QVF) were utilized to ensure the precise design. Through this exhaustive scanning of 21 residues of the tBimBH3 peptide for each target protein, a total of 5,544 mutations were generated. For the purpose of evaluating ncAAs mutations in silico design accuracy, we focused on the evaluations of the energy influence of the mutated side chains to select favorable mutations. This involved measuring the differences in binding affinity (dAffinity) and thermostability (dStability) of the mutant residues compared to the reference complex (**Figure** **2**A). By considering an ensemble of conformations and calculating the Boltzmann average affinities and stability, we performed a comprehensive assessment of how mutations affect binding affinity and protein stability across multiple states, rather than relying on a single static conformation. The effectiveness of this approach is supported by similar methodologies used in antibody and peptide inhibitor design studies,^[^ ^24^ ^]^ where residue scan methodologies and molecular dynamics (MD) simulations are employed to assess binding free energies and stability changes, ensuring a thorough evaluation of potential inhibitors. Two cut‐off criteria (dAffinity ≤ −1 and dStability ≤ −0.5) were applied to filter mutations that significantly enhanced binding to both Mcl‐1 and BCL‐xL. This process identified four favorable mutations: E55(Bpa), I56(Omt), I58(Chg), and F69(MeF) (Figure 2B and Table S1, Supporting Information).

The resulting mutation permutations were subsequently validated using a cell‐free TR‐FRET assay (Figure S2A–I, Supporting Information). The analysis of BH3 variant sequences from B to I with respect to the native tBimBH3 (peptide A) reveals the impact of specific ncAAs mutations on binding affinity to MCL‐1 and BCL‐xL (Figure 2C; Figure S3A–D, Supporting Information). Variant B integrates all four selected mutations (E55(Bpa), I56(Omt), I58(Chg), and F69(MeF)), unexpectedly resulting in IC_50_ values of 121.9 nm for MCL‐1 and 411.6 nm for BCL‐xL, which indicates a suboptimal binding conformation, suggesting that one or more mutation may be detrimental to the binding efficacy for both targets. Excluding E55(Bpa) in variant C improved IC_50_ values to 48.9 nm for MCL‐1 and 278.4 nm for BCL‐xL, yet still inferior to the natural peptide A. Variant D, with I58(Chg) and F69(MeF), demonstrated moderate binding enhancement with IC_50_ values of 25.36 nm for MCL‐1 and 58.51 nm for BCL‐xL. Variant E, characterized by the F69(MeF) mutation, yielded IC_50_ values of 34.38 nm for MCL‐1 and 137.8 nm for BCL‐xL, signifying a diminished binding affinity in comparison to variant D. This suggests that the F69(MeF) mutation might introduce additional steric hindrance from the benzyl group, thereby reducing the binding affinity. Notably, variant F, incorporating the I58(Chg) mutation, exhibited a significant enhancement with IC_50_ values of 9.77 nm for MCL‐1 and 30.79 nm for BCL‐xL, underscoring a substantial improvement in binding efficacy. To corroborate the binding attributes conferred by the I58(Chg) mutation, variant G was examined to include the mutations E55(Bpa), I56(Omt), and F69(MeF). This variant yielded IC_50_ values of 205.8 nm for MCL‐1 and 565.1 nm for BCL‐xL, exhibits a detrimental binding profile compared to other variants. Therefore, the I58(Chg) mutation is identified as a pivotal favorable mutation that significantly enhances the bispecific binding affinity to MCL‐1 and BCL‐xL, thereby establishing its critical role in the optimization of peptide binding profiles.

Building upon previous studies that have demonstrated the significant influence of BH3 peptide lengths, ranging from 16 to over 30 amino acids, on their binding affinity to anti‐apoptotic proteins, with Bim peptides showing affinities from less than 10 to over 100 nm for BCL‐xL, MCL‐1, and related proteins,^[^ ^25^ ^]^ we synthesized variant H (BimBH3) as an elongated form of tBimBH3 to investigate the effect of extended residues on the N‐ and C‐termini on binding efficacy. This variant displayed enhanced binding affinity and specificity, with IC_50_ values of 10.26 nm for MCL‐1 and 44.98 nm for BCL‐xL, which are superior to those of peptide A (Figure 2C). These findings corroborate the notion that the extension of tBimBH3 termini augments co‐targeting affinity, aligning with the established trend of increased peptide length correlating with greater binding potency.^[^ ^26^ ^]^ Moreover, Peptide‐I, incorporating the pivotal mutation, displayed an exceptional binding profile, with IC_50_ values of 2.77 nm for MCL‐1 and 10.69 nm for BCL‐xL, reflecting an approximately fourfold increase in binding affinity compared to BimBH3. Collectively, while variant F exhibited notable enhancements in binding affinity, Peptide‐I, bearing elongated terminal residues, emerged as the most promising candidate, characterized by its superior IC_50_ values, which indicate its potential therapeutic efficacy in engaging MCL‐1 and BCL‐xL.

To compare the stability and conformational dynamics of target protein complexes with BimBH3 (Peptide‐H) and Peptide‐I, we measured the Root Mean Square Deviation (RMSD) over 500 ns MD simulations (Figure 2D,E; Figure S4A,B, Supporting Information). For the MCL‐1: BimBH3 complex, the protein RMSD stabilizes ≈4 Å with persistent fluctuations, while the ligand RMSD averages 2.0–2.5 Å, indicating movement within the binding pocket. In contrast, the MCL‐1: Peptide‐I complex shows more stable interactions, with protein RMSD stabilizing at 3.5–4 Å and fewer fluctuations, and ligand RMSD averaging ≈1.5–2 Å, reflecting a tighter binding profile. For the BCL‐xL: BimBH3 complex, the protein RMSD stabilizes near 4.5–5 Å with variability, and the ligand RMSD averages 2.5–3 Å. However, the BCL‐xL: Peptide‐I complex exhibits significantly improved stability, with protein RMSD ≈4–4.5 Å and ligand RMSD consistently lower at ≈1.5–2 Å. Across both MCL‐1 and BCL‐xL complexes, Peptide‐I demonstrates greater stability and reduced conformational changes compared to BimBH3, suggesting the formation of more stable interactions, contributing to enhanced binding stability and affinity within the binding pocket. This observation is further substantiated by the results in Figure S4C (Supporting Information), comparing RMSD values of complexes over 0–500 ns and 250–500 ns periods. For the MCL‐1: BimBH3 complex, protein RMSD values are 3.89 ± 0.43 Å and 3.94 ± 0.26 Å, with ligand RMSD at 1.90 ± 0.30 Å and 1.87 ± 0.23 Å. In contrast, MCL‐1: Peptide‐I exhibits lower protein RMSD (3.60 ± 0.30 Å and 3.68 ± 0.18 Å) and reduced ligand RMSD (1.78 ± 0.22 Å and 1.78 ± 0.24 Å), indicating enhanced stability. Similarly, BCL‐xL: BimBH3 shows protein RMSD of 4.17 ± 0.58 Å and 4.36 ± 0.62 Å, with ligand RMSD of 2.10 ± 0.41 Å and 2.12 ± 0.47 Å. The BCL‐xL: Peptide‐I complex maintains similar protein stability (4.21 ± 0.56 Å and 4.42 ± 0.41 Å) but exhibits significantly lower ligand RMSD (1.69 ± 0.23 Å and 1.72 ± 0.23 Å). The consistently reduced RMSD values suggest Peptide‐I achieves superior binding stability and conformational control. The ΔGbind values further confirmed the superior binding of Peptide‐I (Figure S3C,D, Supporting Information), with all binding free energy values in this study determined using the Molecular Mechanics Poisson–Boltzmann Surface Area (MMPBSA) method. The ΔGbind for Peptide‐I was −21.28 kcal mol^−1^ for MCL‐1 and −17.40 kcal mol^−1^ for BCL‐xL, which were more favorable than those of BimBH3, with respective values of −19.47 kcal mol^−1^ for MCL‐1 and −16.15 kcal mol^−1^ for BCL‐xL.

The 3D visualization of MCL‐1 and BCL‐xL complexes with various peptides correlates computational (W‐RMSD, ΔGbind) and experimental (IC_50_) evaluations (Figure 2F,G). W‐RMSD, calculated with equal weights (𝑤₁ = 𝑤₂ = 0.5) over 250–500 ns, showed agreement with experimental IC_50_ values. For instance, Peptide‐I had the lowest W‐RMSD and most favorable binding free energy, achieving the best IC_50_ values (2.77 nm for MCL‐1 and 10.69 nm for BCL‐xL), while Peptide‐G, with high W‐RMSD and less favorable binding energy, exhibited poor IC_50_ performance. This validates the accuracy and reliability of the computational pipeline in predicting peptide binding efficacy. Furthermore, the ΔGbind strongly correlates with experimental IC_50_ values, with Pearson correlation coefficients of r = 0.966 (*p* = 2.34 × 10^−5^) for MCL‐1 and r = 0.969 (*p* = 1.60 × 10^−5^) for BCL‐xL (Figure 2H,I), validating the reliability of computational pipeline. Additionally, W‐RMSD values show significant positive correlations with IC_50_ measurements (r = 0.948, *p* = 1.03 × 10^−4^ for MCL‐1 and r = 0.851, *p* = 3.6 × 10^−3^ for BCL‐xL), and with ΔGbind (Figure S3E,F, Supporting Information), indicating that lower W‐RMSD values are associated with more favorable binding free energy. The results demonstrate that lower W‐RMSD values, indicative of more stable peptide‐protein interactions, correlate with stronger experimental binding affinity. Integrating ΔGbind and W‐RMSD with experimental IC_50_ data provides a comprehensive approach to enhance peptide engineering strategies.

The structural analyses of Peptide‐I complexes with BCL‐xL and MCL‐1 were visualized to elucidate its binding modes within the key binding pockets of MCL‐1 (Figure 2J,K) and BCL‐xL (Figure 2L,M). Peptide‐I is observed to form a helical conformation in both complexes, engaging in key hydrophobic interactions of Chg58 with BCL‐xL and MCL‐1 residues, such as L68, L72, V86, and F106 in BCL‐xL, and M231, L235, V249, F270 in MCL‐1. Moreover, structural analysis of the MCL‐1: Peptide‐I complex elucidates additional hydrogen bonding interactions pivotal for enhanced binding affinity and stability (Figure 2K). Key residues R25 and D323 form an H‐bond securing Peptide‐I within the binding pocket of MCL‐1. The backbone bond of F318 and E317 between R25 stabilizes the complex, maintaining structural integrity for effective binding, while the H‐bond between the Y23 backbone and R25 further reinforces stability and contributes to its high binding affinity and low RMSD values observed in simulations. It is noteworthy that although the extended terminal residues of Peptide‐I display relatively weaker intermolecular interactions with BCL‐xL, only the longer Bim peptides can activate BAX, thereby inducing mitochondrial outer membrane permeabilization and ultimately triggering apoptosis (Figure 2M).^[^ ^27^ ^]^ These functional interactions, consistent with the superior binding properties of Peptide‐I, are fundamental to its improved affinity and stability, corroborating binding affinity data and MD simulations. The insights from this analysis provide valuable guidance for peptide‐based therapeutic development of co‐targeting MCL‐1 and BCL‐xL.

### 2.2 Evaluation of BH3 Variant Cytotoxicity and Targeted Peptide Enhancements in Leukemia Cell Lines

**Figure 3:**
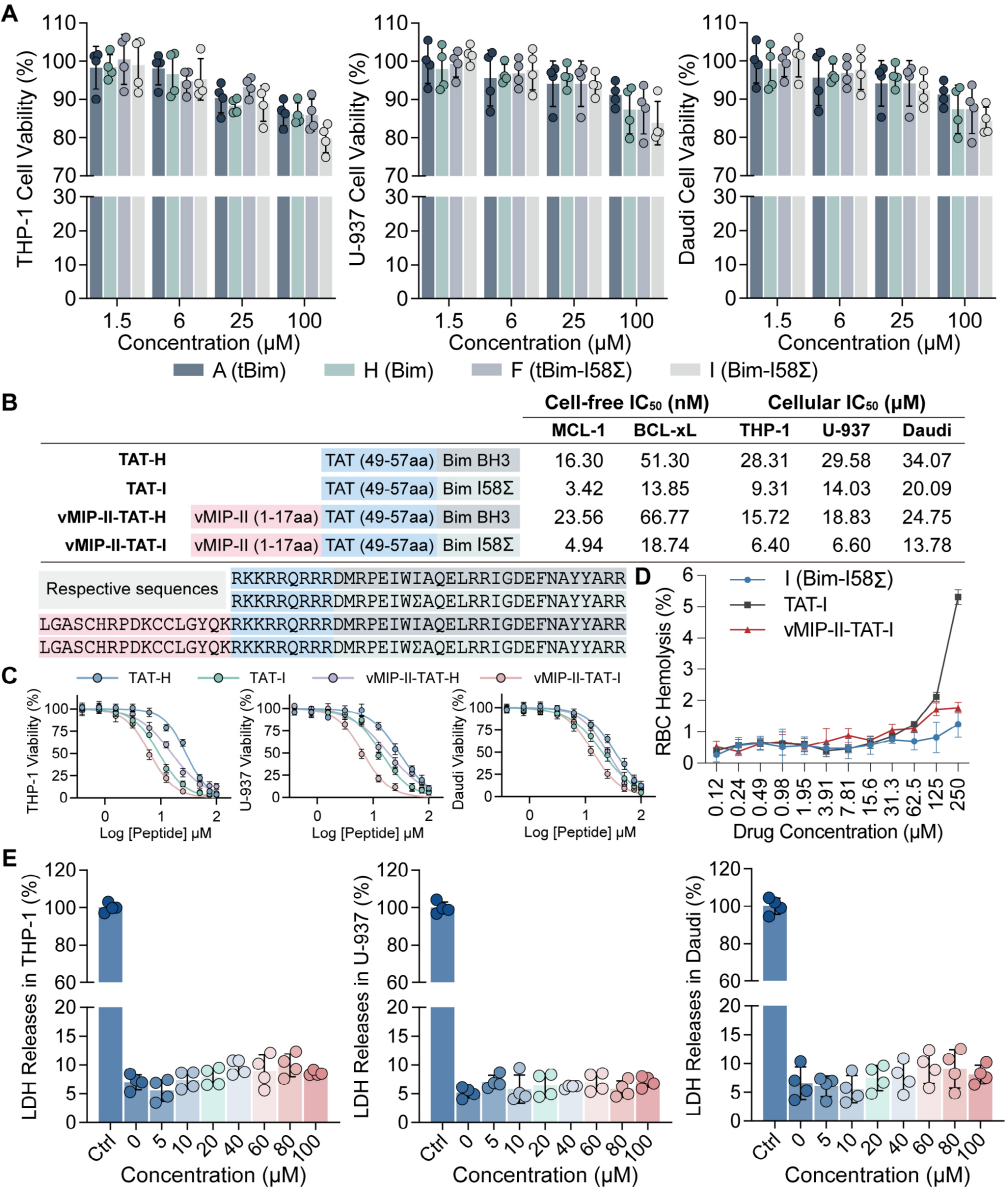
Cytotoxicity and cellular selectivity of functional BH3 peptides. A) Cell viability was assessed in THP‐1, U‐937, and Daudi cell lines following treatment with Peptide‐A, Peptide‐H, Peptide‐F, and Peptide‐I at concentrations ranging from 1.5 to 100 µm (*n* = 4). B) The IC_50_ values of various BH3 variants fused with TAT and vMIP‐II‐TAT were assessed in both cell‐free and cellular contexts. Cell‐free IC_50_ measurements were conducted for MCL‐1 and BCL‐xL, whereas cellular IC_50_ values were determined in THP‐1, U‐937, and Daudi cell lines. C) The proliferation of leukemia cells in THP‐1, U‐937, and Daudi cell lines was inhibited by BH3‐mimetic peptides introduced at concentrations ranging from 0 to 100 µm (*n* = 4). Cell viability was measured after 48 h using the CCK‐8 assay. D) Hemolytic activity of Peptide‐I (Bim‐I58(Chg)), TAT‐I (TAT‐Bim I58(Chg)), and vMIP‐II‐TAT‐I (vMIP‐II‐TAT‐Bim I58(Chg)) was evaluated across a concentration gradient from 0.12 to 250 µm (*n* = 4). The percentage of red blood cell (RBC) hemolysis was measured to assess the safety profile of each peptide. E) Lactate dehydrogenase (LDH) release assays were performed to assess the impact of vMIP‐II‐TAT‐I on cell membrane integrity in THP‐1, U‐937, and Daudi cell lines, with concentrations ranging from 5 to 100 µm (*n* = 4). Error bars represent mean ± SD. *p* values were calculated by two‐way (A) or one‐way (E) analysis of variance (ANOVA), with both comparisons not significant (ns, *p* > 0.05).

Next, we assessed the cell‐free activity and in vitro cytotoxicity of various BH3 peptides, including Peptide‐A (tBimBH3), Peptide‐H (BimBH3), Peptide‐F (tBimBH3 with I58(Chg) mutation), and Peptide‐I (BimBH3 with I58(Chg) mutation). As shown in **Figure** **3**A, these peptides exhibited minimal pro‐apoptotic effects in THP‐1, U‐937, and Daudi cell lines at concentrations from 1.5 to 100 µm, with high cell viability observed except for a modest reduction to 77% in THP‐1 cells. This suggests that these BH3 peptides, in their current forms, are inefficient at inducing apoptosis, likely due to insufficient cellular permeability. To enhance cellular uptake, we conjugated the TAT cell‐penetrating peptide (CPP) to the N‐terminus of Peptide‐H and Peptide‐I (Figure S2J,K, Supporting Information). The modified peptides, TAT‐H and TAT‐I, displayed significantly reduced IC_50_ values in the low micromolar range (Figure 3B,C). Specifically, TAT‐I had an IC_50_ of 9.31 µm in THP‐1 cells, approximately three‐fold lower than TAT‐H (28.31 µm), and 14.03 µm in U‐937 cells, ≈1.5 times lower than TAT‐H (29.58 µm). In Daudi cells, TAT‐I showed an IC_50_ of 20.09 µm, compared to 34.07 µm for TAT‐H. These results indicate the critical role of the TAT CPP in improving cellular uptake and the enhanced cytotoxic efficacy of TAT‐I, attributed to the I58(Chg) mutation.

Given the non‐specificity of the current Peptide‐I formulation, which may lead to off‐target effects such as apoptosis in healthy cells, improving its selectivity is essential to avert such adverse outcomes. Considering the overexpression of CXCR4 in leukemia and HSCs, targeting CXCR4 is a promising therapeutic approach for hematological malignancies. Consequently, a CXCR4‐targeted variant, vMIP‐II‐TAT‐I, was engineered by integrating the vMIP‐II chemokine subdomain (LGASWHRPDKCCLGYQK) to enhance specificity (Figure S2L,M, Supporting Information). Comparative analysis revealed that both vMIP‐II‐TAT‐I and its reference, vMIP‐II‐TAT‐H, exhibited significantly lower IC_50_ values than their non‐targeted counterparts (Figure 3B,C). Specifically, vMIP‐II‐TAT‐H showed IC_50_ values of 15.72 µm in THP‐1, 18.83 µm in U‐937 cells, and 24.75 µm in Daudi. Conversely, vMIP‐II‐TAT‐I achieved lower IC_50_ values of 6.40 µm in THP‐1, 6.60 µm in U‐937 cells, and 13.78 µm in Daudi. Additionally, in cell‐free assays, vMIP‐II‐TAT‐I demonstrated strong nanomolar binding affinities to MCL‐1 (4.94 nm) and BCL‐xL (18.74 nm) (Figure 3B; Figure S5A,B, Supporting Information). This substantial decrease in IC_50_ values suggests a significant enhancement in the cytotoxic efficacy and selectivity of vMIP‐II‐TAT‐I. This targeted approach is anticipated to enhance anti‐leukemic efficacy while reducing off‐target effects, marking a strategic advancement in the development of more selective and potent peptide‐based therapies for leukemia.

The stability analysis of Peptide‐H, Peptide‐I, vMIP‐II‐TAT‐H, and vMIP‐II‐TAT‐I in 10% plasma over a 4‐h incubation period highlights differences between the two comparative sets: Peptide‐H versus Peptide‐I and vMIP‐II‐TAT‐H versus vMIP‐II‐TAT‐I (Figure S5C, Supporting Information). Peptide‐I demonstrates superior stability with slower degradation compared to Peptide‐H, as evidenced by its stronger and more persistent band intensities over time in SDS‐PAGE analysis (Figure S5D, Supporting Information). Similarly, vMIP‐II‐TAT‐I shows improvement in plasma stability over vMIP‐II‐TAT‐H, maintaining band intensities throughout the incubation period and reflecting slower degradation rates. These findings indicate that introduced modifications resulted in enhanced plasma stability,^[^ ^28^ ^]^ with Peptide‐I and vMIP‐II‐TAT‐I consistently demonstrating superior performance compared to their natural counterparts.

The hemolysis assay, which quantifies red blood cell (RBC) membrane lysis, was employed to assess the safety profiles of Peptide‐I, TAT‐I, and vMIP‐II‐TAT‐I (Figure 3D). In a hemolysis study across a concentration gradient from 0.12 to 250 µm, the peptides exhibited minimal hemolytic activity (<2%) at lower concentrations, aligning with the therapeutic safety threshold. However, at elevated concentrations, TAT‐I surpassed the safety threshold, reaching over 5% hemolysis, whereas Peptide‐I and vMIP‐II‐TAT‐I kept hemolysis below 2%. The incorporation of the vMIP‐II domain in vMIP‐II‐TAT‐I significantly reduced the hemolytic potential of the TAT motif, indicating its enhanced safety as a therapeutic candidate. This diminished hemolytic effect, combined with efficient cellular penetration, is likely to improve the efficacy and safety of BH3‐based therapeutics.

To determine the mode of cell death induced by vMIP‐II‐TAT‐I in THP‐1, U‐937, and Daudi cell lines, lactate dehydrogenase (LDH) release was monitored after exposure to concentrations ranging from 5 to 100 µm. The data (Figure 3E) revealed negligible LDH release across all tested concentrations akin to untreated controls, indicating that vMIP‐II‐TAT‐I maintains cell membrane integrity. The Triton X‐100 (positive control) induced substantial LDH release, confirming assay sensitivity. These observations suggest that vMIP‐II‐TAT‐I does not significantly induce necrosis, as evidenced by the unchanged LDH levels, and supports the likelihood of apoptosis as the primary cell death mechanism. Collectively, the results thus suggest that vMIP‐II‐TAT‐I can selectively induce leukemia cell death without extensive membrane damage, presenting a safer therapeutic profile.

**Figure 4:**
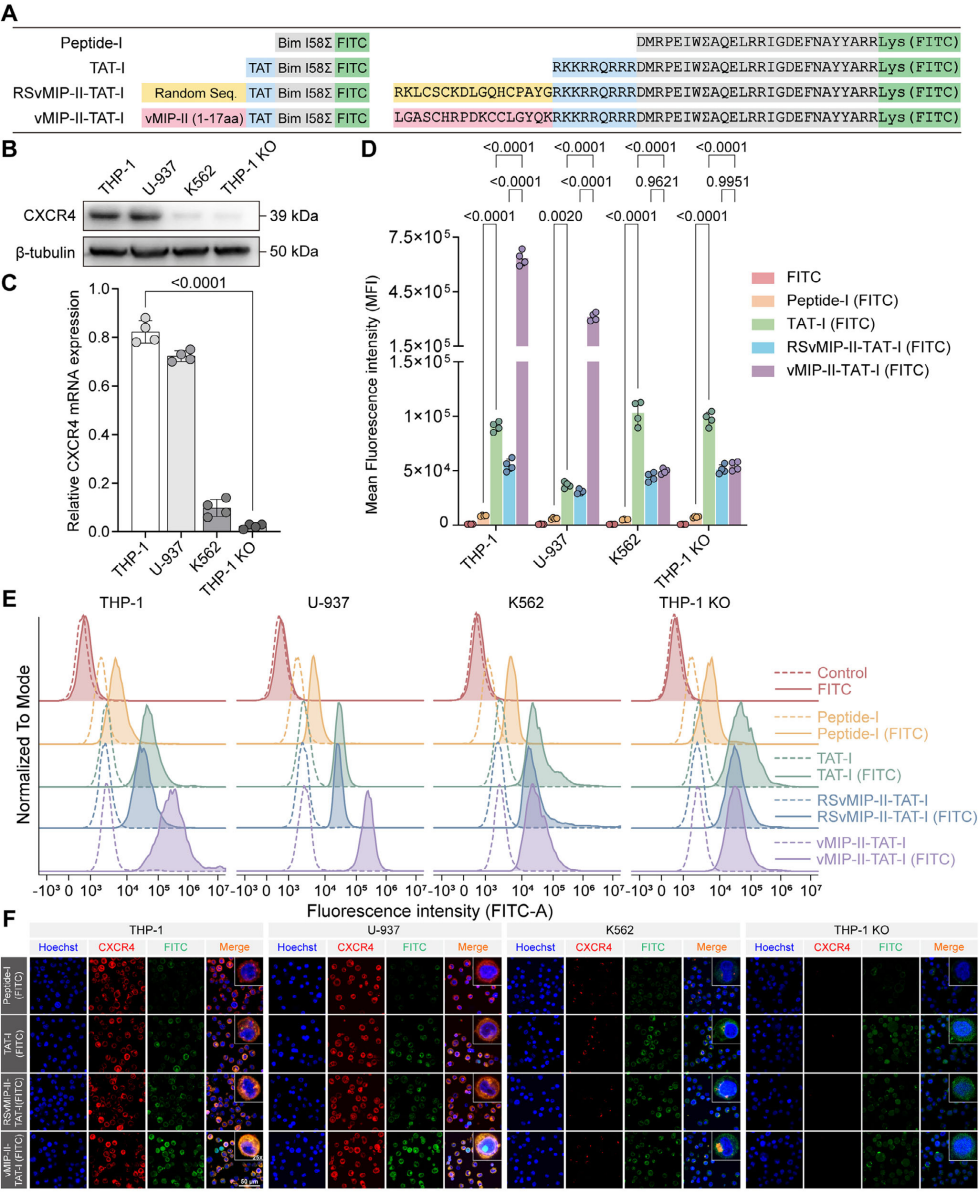
Cellular uptake and localization of FITC‐labeled peptide derivatives. A) Sequences of Peptide‐I derivatives, including TAT‐I, RSvMIP‐II‐TAT‐I, and vMIP‐II‐TAT‐I, conjugated to fluorescein isothiocyanate (FITC) for fluorescence‐based visualization. B,C) Western blot and PCR (*n* = 4) analyses confirmed CXCR4 expression levels in THP‐1, U‐937, K562, and THP‐1‐CXCR4 KO cells. D,E) Flow cytometry analysis, presented as bar graphs and histograms, quantifies peptide uptake efficiency across cell lines (*n* = 4). Mean fluorescence intensity (MFI) values of FITC‐labeled peptides were determined after subtracting background signals from non‐FITC‐labeled controls. F) Confocal microscopy images depicting intracellular localization of FITC‐labeled peptides (green) relative to CXCR4 (red) and nuclei (blue) in the respective cell lines. Error bars represent mean ± SD. Statistical analysis was performed using Student's *t*‐test for (C) and two‐way ANOVA for (D); *p* < 0.05 was considered statistically significant.

Following the characterization of cytotoxicity and cellular selectivity, we examined the cellular uptake efficiency of Peptide‐I (FITC), TAT‐I (FITC), RSvMIP‐II‐TAT‐I (FITC), and vMIP‐II‐TAT‐I (FITC) across AML cell lines with varying CXCR4 expression levels, utilizing FITC‐labeled peptides for detection (**Figure** **4**A; Figure S2N–Q, Supporting Information). To further validate the specificity of vMIP‐II‐TAT‐I for CXCR4 on AML cell surfaces, we generated a THP‐1‐CXCR4 knockout (KO) cell line and assessed CXCR4 expression at both the protein and mRNA levels across THP‐1, U‐937, K562, and THP‐1‐CXCR4 KO cells. Western blot analysis (Figure 4B) confirmed robust CXCR4 expression in THP‐1 and U‐937 cells, low expression in K562 cells, and complete absence in the THP‐1‐CXCR4 KO line. These findings were corroborated by RT‐qPCR analysis of relative CXCR4 mRNA levels (Figure 4C), which demonstrated high expression in THP‐1 and U‐937 cells, reduced expression in K562 cells, and negligible expression in the THP‐1‐CXCR4 KO cells. The THP‐1‐CXCR4 KO cell line serves as a robust model to investigate the dependence of vMIP‐II‐TAT‐I cellular uptake on CXCR4 and to differentiate specific CXCR4‐mediated interactions from the nonspecific uptake observed with TAT‐I and RSvMIP‐II‐TAT‐I.

Flow cytometry analysis (Figure 4D) revealed significant differences in MFI among the tested peptides. To ensure accuracy and specificity, a rigorous gating strategy was employed, incorporating appropriate controls: untreated cells (PBS control), free FITC (FITC control), and both FITC‐labeled and unlabeled derivatives of Peptide‐I and its variants (Figure 4E). This approach minimized nonspecific signals and ensured the reliability of the observed differences. In THP‐1 cells, which express high levels of CXCR4, vMIP‐II‐TAT‐I (FITC) exhibited the highest cellular uptake, with an MFI of 685,011, ≈73.9‐fold higher than Peptide‐I (FITC) (9,264), 7.2‐fold higher than TAT‐I (FITC) (95,167), and 13.0‐fold higher than RSvMIP‐II‐TAT‐I (FITC) (52,677). A similar trend was observed in CXCR4‐expressing U‐937 cells, where vMIP‐II‐TAT‐I (FITC) displayed the highest MFI (288,528), corresponding to a 53.7‐fold increase compared to Peptide‐I (FITC) (5,373), 8.6‐fold compared to TAT‐I (FITC) (33,589), and 9.7‐fold compared to RSvMIP‐II‐TAT‐I (FITC) (29,770). These findings indicate that vMIP‐II enhances the CXCR4‐targeting capacity of TAT‐I, leading to the highest uptake efficiency in CXCR4‐positive cells. Conversely, RSvMIP‐II‐TAT‐I, which contains a scrambled vMIP‐II sequence, exhibited markedly lower cellular uptake, suggesting a loss of CXCR4 specificity.

In contrast, in K562 cells, which have low CXCR4 expression, TAT‐I (FITC) showed the highest MFI (98,019), surpassing RSvMIP‐II‐TAT‐I (FITC) (42,326) by 2.5‐fold and vMIP‐II‐TAT‐I (FITC) (46,614) by 2.1‐fold. Similarly, in CXCR4‐deficient THP‐1‐CXCR4 KO cells, TAT‐I (FITC) demonstrated the strongest cellular uptake (MFI = 104,480), 1.8‐fold higher than RSvMIP‐II‐TAT‐I (FITC) (56,757) and 1.8‐fold higher than vMIP‐II‐TAT‐I (FITC) (57,378). These results confirm that TAT‐I exhibits nonspecific membrane penetration in the absence of CXCR4, consistent with previous findings on TAT‐mediated peptide translocation across cell membranes.^[^ ^29^ ^]^ Notably, the inclusion of the vMIP‐II motif in vMIP‐II‐TAT‐I substantially enhanced CXCR4 specificity, leading to reduced MFI values in CXCR4‐deficient K562 and THP‐1‐CXCR4 KO cells. The spatial arrangement of the TAT sequence within the peptide appears to modulate its membrane‐penetrating efficiency, with the central positioning of TAT in vMIP‐II‐TAT‐I likely attenuating nonspecific cellular uptake in CXCR4‐negative cells.

Confocal microscopy imaging (Figure 4F) further corroborates the flow cytometry findings, providing spatial visualization of FITC‐labeled peptide co‐localization with CXCR4 in THP‐1, U‐937, K562, and THP‐1‐CXCR4 KO cells. The co‐localization of FITC fluorescence (green) from Peptide‐I derivatives with CXCR4 (red) is depicted as orange in the merged images, highlighting the uptake specificity of vMIP‐II‐TAT‐I (FITC) in CXCR4‐expressing cells. In THP‐1 and U‐937 cells, which express high levels of CXCR4, vMIP‐II‐TAT‐I exhibited prominent orange signals, indicative of strong co‐localization with CXCR4. In contrast, RSvMIP‐II‐TAT‐I (FITC) and TAT‐I (FITC) displayed weaker and more diffuse green fluorescence, suggesting reduced or nonspecific uptake. In K562 cells, characterized by low CXCR4 expression, and THP‐1‐CXCR4 KO cells, which lack CXCR4 entirely, all peptides exhibited minimal or no co‐localization, implying the requirement of CXCR4 for vMIP‐II‐TAT‐I cellular uptake. Notably, the structural design of vMIP‐II‐TAT‐I, in which the TAT sequence is positioned centrally, appears to mitigate nonspecific membrane penetration. Compared to TAT‐I, which lacks a targeting motif and exhibits greater nonspecific uptake, vMIP‐II‐TAT‐I demonstrates enhanced specificity for CXCR4‐positive cells, likely contributing to its improved uptake stability.

### 2.3 Selective and Synergistic Apoptosis Induction and Cell Viability Inhibition by vMIP‐II‐TAT‐I and Venetoclax in AML and HSC Models

**Figure 5:**
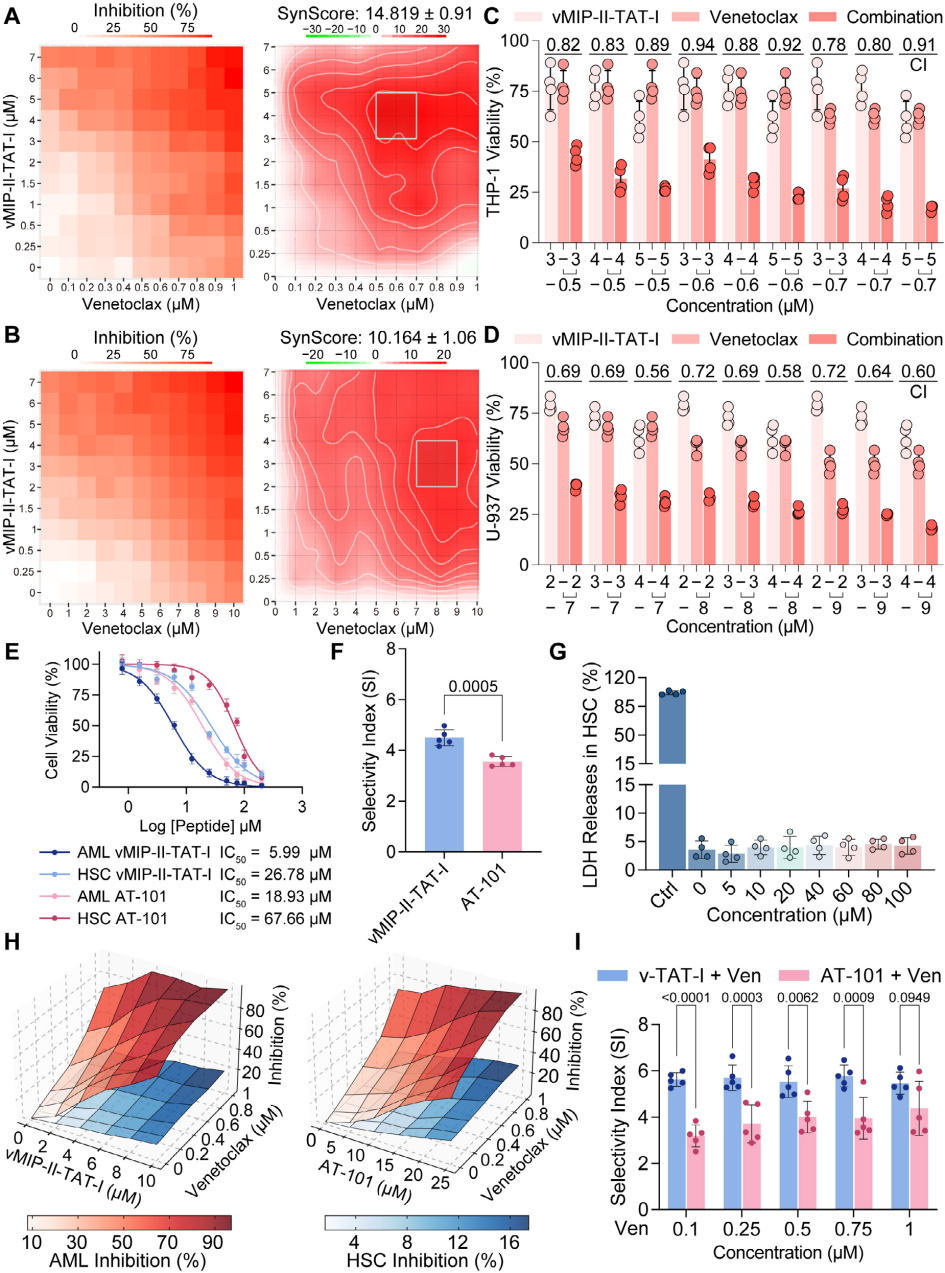
Synergistic effects and selectivity of vMIP‐II‐TAT‐I and AT‐101 in AML and HSC models. A,B) Heatmaps showing the inhibition rates of THP‐1 (A) and U‐937 (B) cells treated with vMIP‐II‐TAT‐I (0–7 µm) and Venetoclax (0–1 µm for THP‐1; 0–10 µm for U‐937). The corresponding right panels depict the ZIP synergy scores, with the highest synergy region highlighted within the square region. Data are represented as means ± SD. C,D) Cell viability analysis of THP‐1 (C) and U‐937 (D) cells within the optimal concentration range for both compounds, with synergy quantified using the Combination Index (CI) (*n* = 4). E) CCK‐8 assay results assessing cell viability in AML (THP‐1) and HSC models following treatment with increasing concentrations of vMIP‐II‐TAT‐I and AT‐101 (*n* = 5). F) Selectivity index (SI) comparison of vMIP‐II‐TAT‐I and AT‐101 in AML cells (*n* = 5). G) LDH release in human HSCs after treatment with vMIP‐II‐TAT‐I at varying concentrations, indicative of cytotoxicity (*n* = 4). H) 3D response surface plots illustrating inhibition rates of AML (THP‐1) and HSC cells within the effective therapeutic concentration ranges of vMIP‐II‐TAT‐I (left) and AT‐101 (right) in combination with Venetoclax. I) SI values for AML cells treated with vMIP‐II‐TAT‐I (0, 2, 4, 6, 8, 10 µm) or AT‐101 (0, 5, 10, 15, 20, 25 µm) in combination with Venetoclax (0, 0.2, 0.4, 0.6, 0.8, 1 µm), assessing treatment specificity for AML relative to HSCs (*n* = 5). Error bars represent mean ± SD. Statistical analysis was performed using one‐way ANOVA for (G), where comparisons were not significant (ns, *p* > 0.05), Student's *t*‐test for (F), and two‐way ANOVA for (I). *p* < 0.05 was considered statistically significant.

The combined treatment of vMIP‐II‐TAT‐I and Venetoclax in THP‐1 and U‐937 cell lines was analyzed using the Zero Interaction Potential (ZIP) model in SynergyFinder (Figure S7A, Supporting Information),^[^ ^30^ ^]^ with inhibition patterns and dose responses visualized through heatmaps and bar graphs. Heatmaps for THP‐1 (**Figure** **5**A) and U‐937 cells (Figure 5B) show inhibition levels across a range of concentrations (0.25–7 µm for vMIP‐II‐TAT‐I and 0.1/1–1/10 µm for Venetoclax), reaching over 75% at higher concentrations. Synergy scores of 14.819 ± 0.91 for THP‐1 and 10.164 ± 1.06 for U‐937 cells indicate strong positive interactions, with contour maps highlighting the best synergy score regions. Combination Index (CI) values were 0.78–0.94 in THP‐1 and 0.56–0.72 in U‐937 cells (Figure 5C,D; Figure S7B,C, Supporting Information). Bar graphs demonstrate reduced viability with combined treatment compared to monotherapies, supporting synergistic effects. Both cell lines show significant synergistic responses, aligning with prior studies on drug synergy in leukemia and lymphoma treatments.^[^ ^31^ ^]^

To assess the selectivity and potential impact of vMIP‐II‐TAT‐I on HSCs, we isolated HSCs from human cord blood (Figure S8, Supporting Information), and compared the cytotoxicity and selectivity of vMIP‐II‐TAT‐I and AT‐101, a known MCL‐1/BCL‐xL inhibitor,^[^ ^32^ ^]^ in AML cells (THP‐1) and HSCs. The cell viability data (Figure 5E; Figure S9C, Supporting Information) show that vMIP‐II‐TAT‐I has a lower IC_50_ in AML cells (5.99 µm) than in HSCs (26.78 µm), indicating higher selectivity for AML cells. This is supported by Figure 5F, where vMIP‐II‐TAT‐I exhibits a higher SI (4.49) compared to AT‐101 (3.56). Although both AML cells and HSCs express CXCR4, vMIP‐II‐TAT‐I selectively binds to AML cells with higher expression of CXCR4, resulting in stronger leukemia inhibition and protection of normal HSCs.^[^ ^33^ ^]^ In contrast, the broader action of AT‐101 against anti‐apoptotic proteins in both cell types leads to a lower SI.

LDH release assays (Figure 5G) confirm that vMIP‐II‐TAT‐I has minimal cytotoxicity on HSCs, highlighting its preferential targeting of AML cells. A 3D dose‐response surface plot (Figure 5H) visualizes the combined inhibitory effects of vMIP‐II‐TAT‐I/AT‐101 and Venetoclax on both AML cells and HSCs, showing that the combination effectively inhibits AML cells while having minimal impact on HSCs across various concentrations. In combination treatment studies (Figure 5I; Figure S9D, Supporting Information), vMIP‐II‐TAT‐I used with Venetoclax demonstrated enhanced selectivity for AML cells, with an increased SI at multiple concentrations, particularly at Venetoclax 0.25, 0.5, and 0.75 µm. The SI for vMIP‐II‐TAT‐I combined with Venetoclax remained high (5.60) compared to AT‐101 (3.85). However, at 1 µm Venetoclax, both vMIP‐II‐TAT‐I and AT‐101 showed similar selectivity profiles, suggesting that higher Venetoclax concentrations may equalize the effects of the two compounds. Nonetheless, vMIP‐II‐TAT‐I maintains superior selectivity at lower Venetoclax concentrations, indicating its potential as a more targeted and effective AML treatment with minimal impact on normal HSCs.

**Figure 6:**
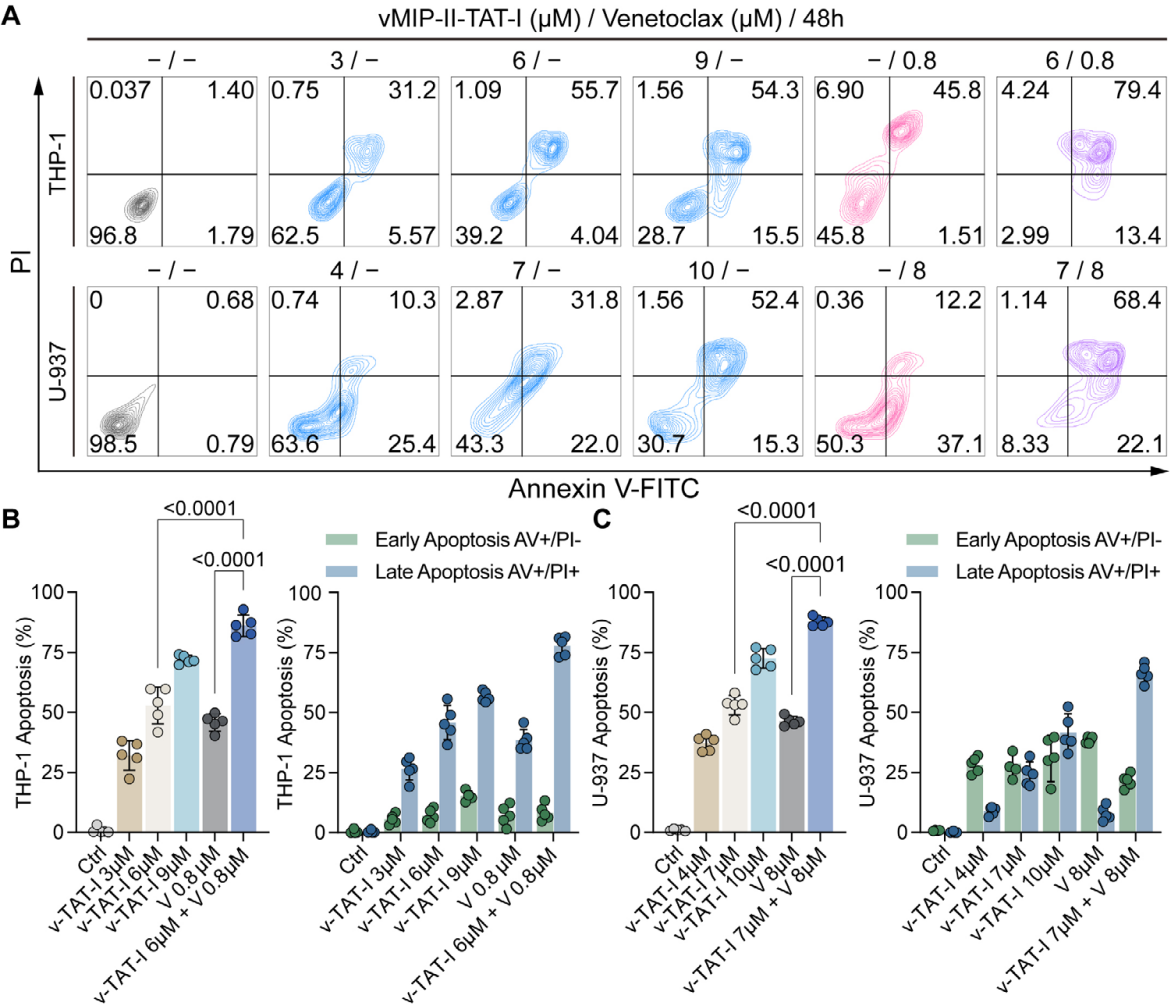
Apoptosis induction by vMIP‐II‐TAT‐I and Venetoclax in THP‐1 and U‐937 cells. A) The induction of apoptosis by vMIP‐II‐TAT‐I, alone or combined with Venetoclax for 48 h in THP‐1 and U‐937 cells, with late and early apoptosis indicated in the upper right and lower right quadrants, respectively. B,C) Dose‐dependent increases in apoptosis are shown for THP‐1 and U‐937 cells (*n* = 5). Error bars represent mean ± SD. Statistical analysis was performed using one‐way ANOVA for (B,C), and *p* < 0.05 was considered statistically significant.

Flow cytometry analysis shows that vMIP‐II‐TAT‐I, alone and in combination with Venetoclax, induces apoptosis in THP‐1 and U‐937 cells over 48 h (**Figure** **6**A). In THP‐1 cells, 3 µm vMIP‐II‐TAT‐I induced 36.8% apoptosis, increasing to 69.8% at 9 µM. Venetoclax alone at 0.8 µm induced 47.3% apoptosis, while the combination of 6 µm vMIP‐II‐TAT‐I with 0.8 µm Venetoclax enhanced apoptosis to 92.8%. In U‐937 cells, 4 µm vMIP‐II‐TAT‐I induced 35.7% apoptosis, increasing to 67.7% at 10 µm. Venetoclax at 8 µm induced 49.3% apoptosis, while the combination of 7 µm vMIP‐II‐TAT‐I with 8 µm Venetoclax increased apoptosis to 90.5%. Quantitative analysis indicates that vMIP‐II‐TAT‐I induces dose‐dependent apoptosis in both cell lines (Figure 6B,C), with combination treatments showing significantly higher rates. This observed synergy aligns with previous reports demonstrating that dual inhibition of MCL‐1 and BCL‐2, such as S63845 combined with Venetoclax, enhances apoptosis in AML cells.^[^ ^34^ ^]^ The differential sensitivity of AML cell lines to single‐agent therapies further supports the enhanced apoptotic response observed in THP‐1 and U‐937 cells upon co‐administration of vMIP‐II‐TAT‐I and Venetoclax.^[^ ^35^ ^]^ Statistical analysis confirmed the significance of these effects. These findings suggest that combining vMIP‐II‐TAT‐I with Venetoclax enhances anti‐leukemic efficacy, potentially allowing for lower drug doses.

### 2.4 vMIP‐II‐TAT‐I Enhances Apoptosis in Leukemia Cells by Disrupting Mitochondrial Membrane Potential and Downregulating Anti‐Apoptotic Proteins

**Figure 7:**
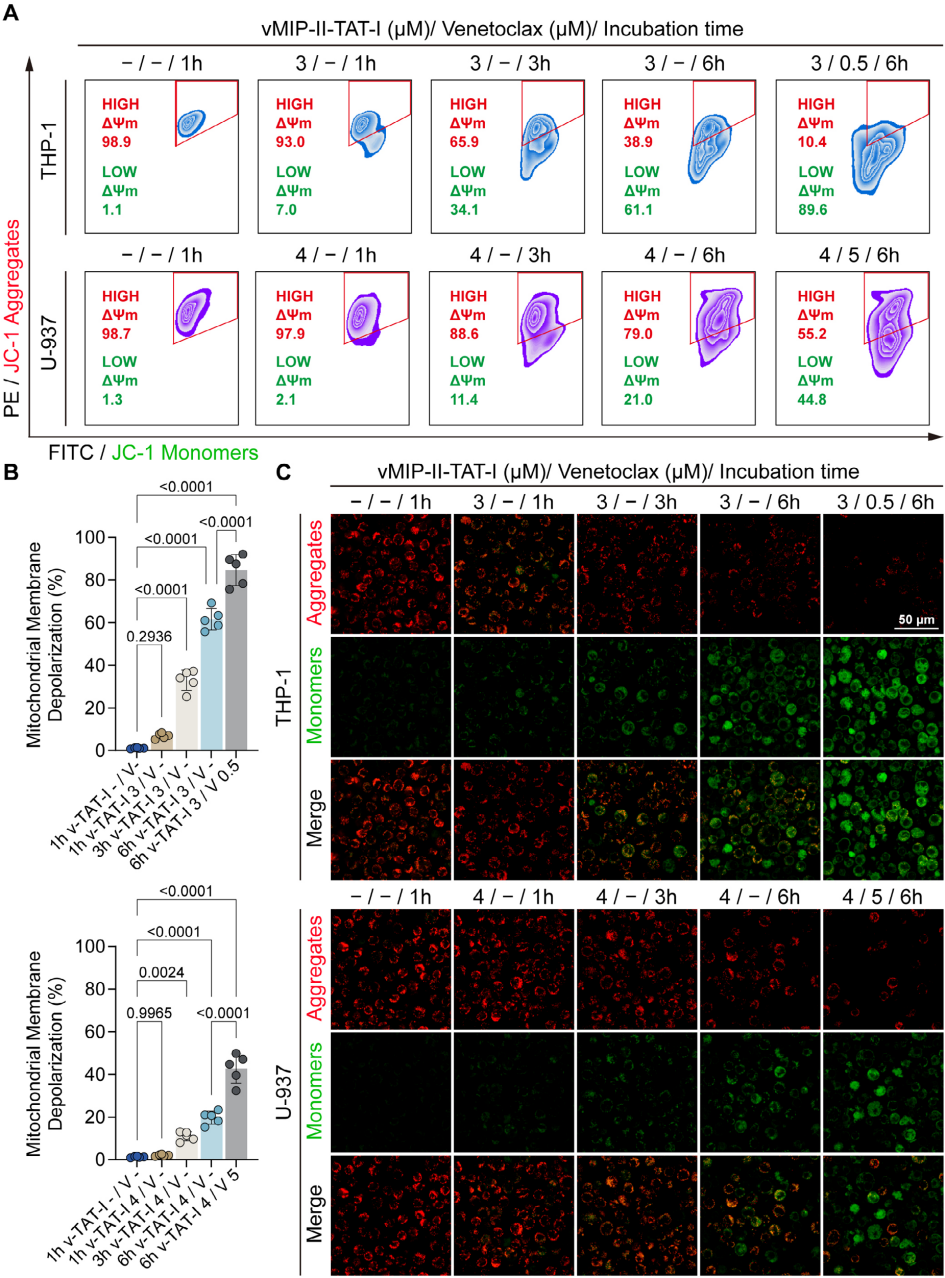
Disruption of mitochondrial membrane potential (MMP) following vMIP‐II‐TAT‐I and Venetoclax treatment. A) Flow cytometry analysis of MMP dynamics in THP‐1 and U‐937 cells treated with vMIP‐II‐TAT‐I alone or in combination with Venetoclax over varying incubation periods. PE/JC‐1 aggregates (red, indicative of intact MMP, high ΔΨm) and FITC/JC‐1 monomers (green, indicative of MMP depolarization, low ΔΨm) were assessed. B) Quantification of mitochondrial depolarization based on flow cytometry data (*n* = 5). C) Confocal microscopy images visualizing MMP disruption, with JC‐1 aggregates (red) marking intact mitochondria and JC‐1 monomers (green) indicating depolarization across different treatment conditions in THP‐1 and U‐937 cells. Error bars represent mean ± SD. Statistical analysis was performed using one‐way ANOVA for (B). *p* < 0.05 was considered statistically significant.

To determine the hallmarks of cellular apoptosis, we subsequently evaluate the impact of vMIP‐II‐TAT‐I and Venetoclax on mitochondrial membrane potential (MMP) in THP‐1 and U‐937 cells. We performed flow cytometry analysis, monitoring the shift from PE/JC‐1 aggregates to FITC/JC‐1 monomers, indicative of mitochondrial depolarization (**Figure** **7**A). In THP‐1 cells, JC‐1 aggregates decreased from 98.9% to 38.9% after 6 h of vMIP‐II‐TAT‐I treatment alone, and to 10.4% with combination treatment, indicating substantial MMP disruption. In U‐937 cells, the combination therapy reduced JC‐1 aggregates to 55.2% after 6 h. Quantitative JC‐1 ratio analysis (Figure 7B) revealed the lowest ratios after 6 h of combination treatment in both cell lines, denoting significant depolarization. These results confirm the synergistic effect of vMIP‐II‐TAT‐I and Venetoclax in disrupting mitochondrial function, consistent with the known mechanism of Venetoclax as a BCL‐2 inhibitor that induces apoptosis by impairing MMP.^[^ ^36^ ^]^ Similarly, A‐1331852, a BCL‐xL inhibitor, induces apoptosis via mitochondrial depolarization (ΔΨm), demonstrating the critical role of mitochondrial integrity in BCL‐2 family inhibitor‐induced apoptosis.^[^ ^37^ ^]^

Confocal microscopy analysis confirms MMP disruption, as indicated by a shift from JC‐1 aggregate‐associated red fluorescence to monomer‐associated green fluorescence in THP‐1 and U‐937 cells (Figure 7C). In THP‐1 cells, red fluorescence remained predominant in untreated controls and after 1‐h vMIP‐II‐TAT‐I treatment, indicating intact MMP. However, a marked increase in green fluorescence, reflecting mitochondrial depolarization and apoptosis, emerged at 3 h and intensified at 6 h in the presence of both vMIP‐II‐TAT‐I and Venetoclax. A similar but less pronounced trend was observed in U‐937 cells, suggesting a cell line‐specific response. These findings align with the apoptotic mechanism of Venetoclax, which disrupts MMP, and indicate that vMIP‐II‐TAT‐I enhances mitochondrial depolarization, potentiating Venetoclax‐induced apoptosis. These results suggest a potent synergistic interaction that may effectively target apoptosis‐resistant cancer cells, offering a promising strategy to overcome therapeutic resistance.

**Figure 8:**
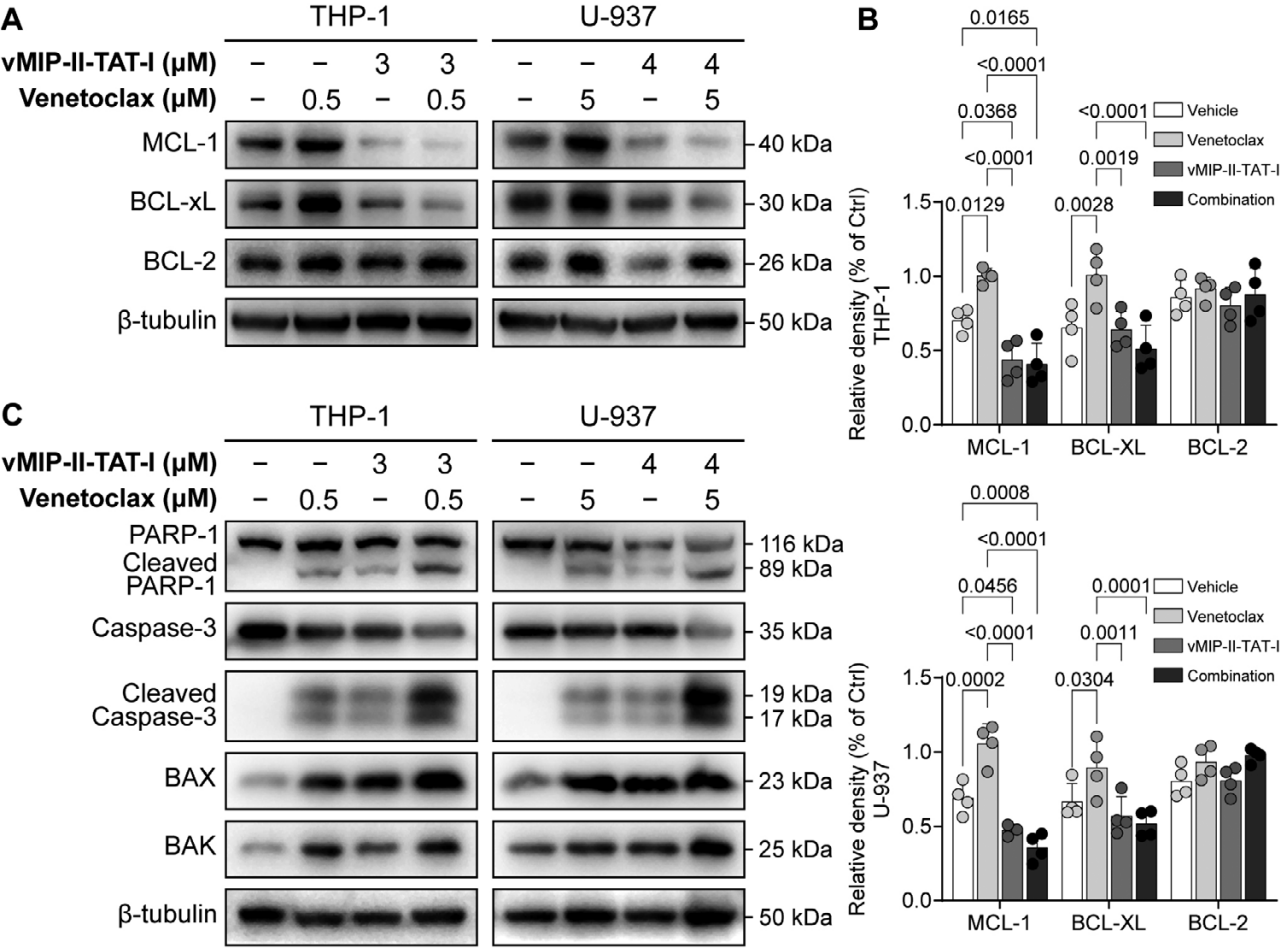
vMIP‐II‐TAT‐I regulates the expression of apoptotic and anti‐apoptotic markers. A) Western blot analysis of MCL‐1, BCL‐xL, BCL‐2, and β‐tubulin expression levels in cells treated with vMIP‐II‐TAT‐I, Venetoclax, and their combination for 6 h. B) Quantification of relative protein expression levels for MCL‐1, BCL‐xL, and BCL‐2 (*n* = 4). C) Western blot analysis of downstream apoptotic markers, including PARP‐1, cleaved PARP‐1, Caspase‐3, cleaved Caspase‐3, BAX, and BAK in cells treated under the same conditions. β‐tubulin was used as an internal reference for standardization. Error bars represent mean ± SD. Statistical analysis was performed using two‐way ANOVA for (B). *p* < 0.05 was considered statistically significant.

Dysregulation of anti‐apoptotic proteins such as MCL‐1, BCL‐xL, and BCL‐2 plays a critical role in the development of drug resistance in hematologic malignancies. Venetoclax is known to induce compensatory upregulation of MCL‐1 and BCL‐xL, enabling cancer cells to evade apoptosis.^[^ ^38^ ^]^ In non‐Hodgkin lymphoma (NHL), dual inhibition of MCL‐1 and BCL‐xL has been shown to enhance the efficacy of Venetoclax, with MCL‐1 suppression significantly amplifying apoptotic responses.^[^ ^39^ ^]^ To investigate this compensatory mechanism, Western blot analysis was performed to assess MCL‐1, BCL‐xL, and BCL‐2 expression in THP‐1 and U‐937 cells under different treatment conditions (**Figure** **8**A). As expected, Venetoclax treatment alone led to a marked upregulation of MCL‐1 and BCL‐xL, likely reflecting an adaptive cellular response to counteract BCL‐2 inhibition. Notably, co‐treatment with vMIP‐II‐TAT‐I significantly reduced MCL‐1 and BCL‐xL levels, potentially overcoming Venetoclax‐induced resistance and restoring apoptosis sensitivity. Importantly, BCL‐2 expression remained unchanged across treatments, suggesting that vMIP‐II‐TAT‐I does not interfere with BCL‐2 transcription or protein stability.

The ability of vMIP‐II‐TAT‐I to downregulate MCL‐1 and BCL‐xL reveals its potential therapeutic role in disrupting leukemia cell survival mechanisms. Quantitative densitometric analysis (Figure 8B) revealed that in THP‐1 cells, combination treatment reduced MCL‐1 and BCL‐xL levels to 40% and 45% of control levels, respectively, while in U‐937 cells, these levels decreased to 30% and 40% of control. These statistically significant reductions indicate a robust suppression of anti‐apoptotic signaling, enhancing apoptotic cell death. Western blot analysis (Figure 8C) shows the expression of key apoptotic markers in THP‐1 and U‐937 cells treated with vMIP‐II‐TAT‐I and Venetoclax individually and in combination. Proteins analyzed include PARP‐1, cleaved PARP‐1, Caspase‐3, cleaved Caspase‐3, BAX, and BAK. In THP‐1 cells, Venetoclax alone slightly increases cleaved PARP‐1 and Caspase‐3, while vMIP‐II‐TAT‐I alone has minimal effect. The combination significantly elevates cleaved PARP‐1, Caspase‐3, BAX, and BAK, indicating synergistic apoptosis enhancement. U‐937 cells show similar trends, with combined treatment markedly increasing cleaved proteins and pro‐apoptotic markers. These findings suggest that vMIP‐II‐TAT‐I, particularly in combination with Venetoclax, effectively counteracts drug resistance by modulating key apoptotic regulators, supporting its potential as a therapeutic strategy in hematologic malignancies.

The synergistic apoptotic response elicited by vMIP‐II‐TAT‐I and Venetoclax stems from their complementary mechanisms of action. This mechanistic interplay parallels findings by Kazi et al., who demonstrated that the BH3 α‐helix mimetic BH3‐M6 effectively binds BCL‐2, BCL‐xL, and MCL‐1, triggering cytochrome c release, caspase activation, and PARP cleavage, ultimately leading to apoptosis.^[^ ^40^ ^]^ Similarly, Venetoclax selectively inhibits BCL‐2, liberating BAX and BAK from sequestration, while vMIP‐II‐TAT‐I downregulates MCL‐1 and BCL‐xL, further alleviating inhibitory constraints on pro‐apoptotic effectors. This dual disruption of anti‐apoptotic proteins shifts the balance toward apoptosis, amplifying downstream signaling cascades and reinforcing an irreversible commitment to programmed cell death.

The significant upregulation of BAX and BAK following combination treatment unveils its ability to enhance mitochondrial outer membrane permeabilization (MOMP), disrupt ΔΨm, and trigger caspase‐dependent apoptotic execution. These findings align with previous studies highlighting the essential roles of BAX and BAK in apoptosis induction,^[^ ^41^ ^]^ particularly in hematologic malignancies where MCL‐1 and BCL‐xL overexpression frequently drive resistance to therapy.^[^ ^42^ ^]^ The suppression of MCL‐1 and BCL‐xL by vMIP‐II‐TAT‐I facilitates the activation of BAX and BAK, intensifying mitochondrial outer membrane permeabilization and driving apoptotic commitment. This disruption of anti‐apoptotic signaling dismantles key resistance mechanisms, shifting the cellular fate toward irreversible apoptosis.

### 2.5 In Vivo Assessment of vMIP‐II‐TAT‐I Mediated CXCR4‐Targeted Dual Inhibition of MCL‐1 and BCL‐xL in AML Treatment

**Figure 9:**
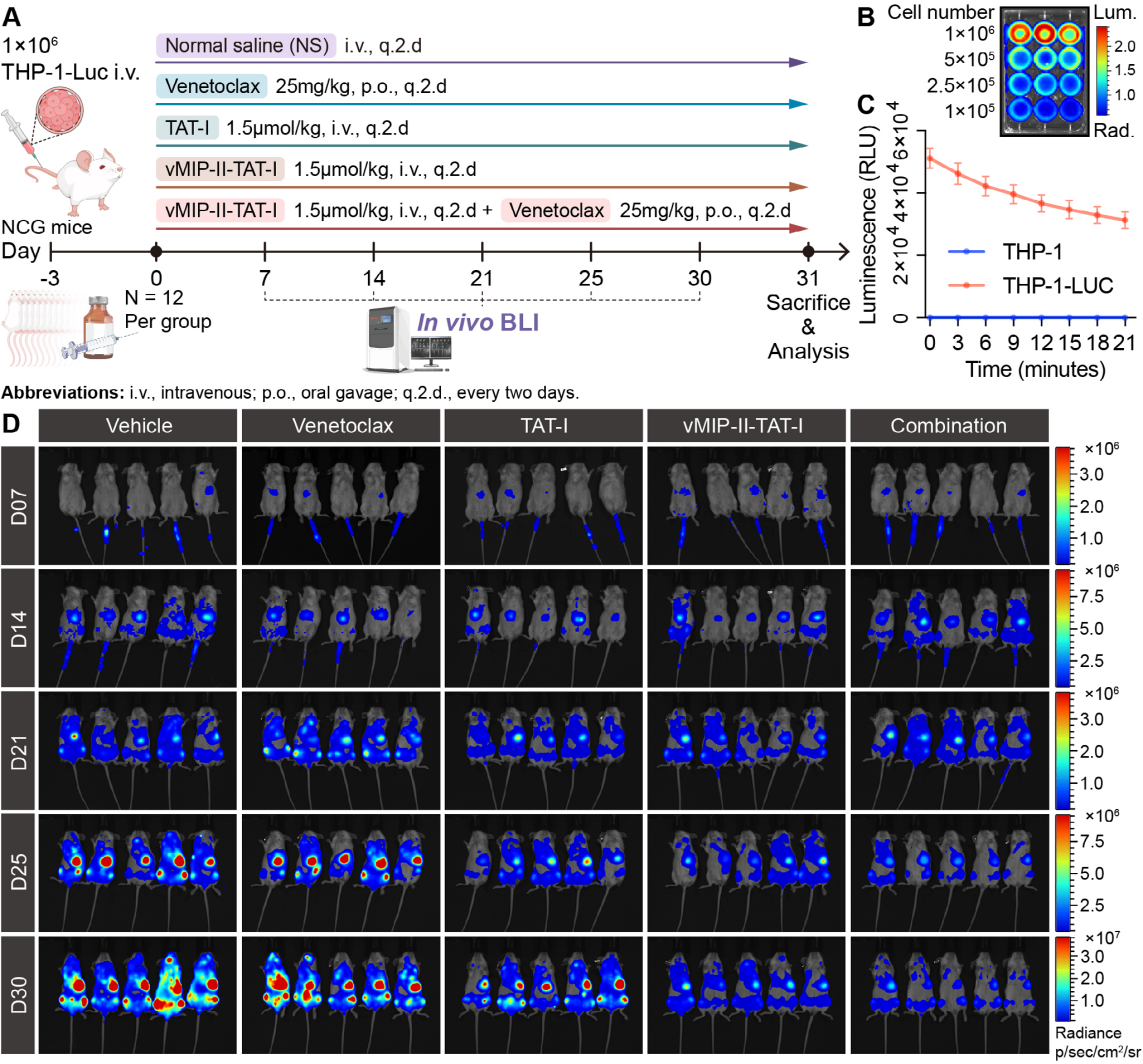
In vivo experimental design and bioluminescence imaging (BLI) assessment. A) Schematic representation of the in vivo treatment regimen in NCG mice inoculated with THP‐1‐Luc cells. Mice received intravenous (i.v.) or oral (p.o.) administration of normal saline (NS), Venetoclax, TAT‐I, vMIP‐II‐TAT‐I, or vMIP‐II‐TAT‐I plus Venetoclax every 2 days (q.2.d.) (*n* = 12). B) BLI validation of THP‐1‐Luc cells, with luminescence intensity correlating with cell number (*n* = 3). C) Time‐dependent luminescence quantification in THP‐1‐Luc and control THP‐1 cells (*n* = 4), confirming BLI sensitivity and signal stability. D) Longitudinal BLI imaging of tumor burden across treatment groups at days 7, 14, 21, 25, and 30, illustrating differential treatment responses (*n* = 5). Error bars represent mean ± SD.

To assess the therapeutic efficacy of vMIP‐II‐TAT‐I‐mediated CXCR4‐targeted dual inhibition of MCL‐1 and BCL‐xL in AML, in vivo studies were conducted using THP‐1 luciferase‐transplanted xenografts in NCG mice (**Figure** **9**A). Five treatment groups (*n* = 12 per cohort) were evaluated: normal saline (NS), Venetoclax (25 mg kg^−1^), TAT‐I (1.5 µmol kg^−1^), vMIP‐II‐TAT‐I (1.5 µmol kg^−1^), and vMIP‐II‐TAT‐I plus Venetoclax (1.5 µmol kg^−1^ + 25 mg kg^−1^). Treatments were administered every 2 days from day 0, with bioluminescence imaging (BLI) performed on days 7, 14, 21, 25, and 30 to monitor tumor progression, and study endpoint at day 31. In vitro BLI validation confirmed luciferase activity in THP‐1‐Luc cells, with luminescence intensity correlating with cell density (Figure 9B). Luminescence decay was initially rapid, stabilizing within 21 min, whereas control THP‐1 cells exhibited negligible signal (Figure 9C). These findings establish the suitability of THP‐1‐Luc cells for real‐time in vivo therapeutic monitoring, providing a reliable platform for evaluating treatment responses.

The BLI of THP‐1‐Luc xenografts in NCG mice, subjected to different treatments for a period of 30 days, is depicted in Figure 9D. On days 7 and 14, negligible differences in tumor burden are noted among the groups. However, by day 21, differences gradually emerge among the groups. By day 25 and 30, a noticeable reduction in radiance is evident in the vMIP‐II‐TAT‐I and Combination therapy groups compared to the Vehicle group, with the Combination group showing the most substantial decrease in radiance values, indicative of a marked reduction in tumor burden. The Vehicle group shows a progressive increase in bioluminescence, signifying uncontrolled tumor growth, while the Venetoclax and TAT‐I groups display only moderate decreases. The sustained decrease in bioluminescence with vMIP‐II‐TAT‐I treatment underscores its efficacy in inhibiting tumor progression. The combination therapy, integrating vMIP‐II‐TAT‐I with Venetoclax, offers the most pronounced therapeutic advantage, with an early and sustained reduction in tumor burden. The observed synergistic effect confirms the enhanced therapeutic efficacy from prior findings, which is plausibly ascribed to the role of Venetoclax in apoptosis induction via BCL‐2 inhibition and the vMIP‐II‐TAT‐I interference of compensatory upregulation of MCL‐1 and BCL‐xL. This dual action is presumed to negate the resistance mechanisms utilized by tumor cells to avert apoptosis, culminating in a significant tumor burden reduction and potentially presenting a promising therapeutic approach for AML treatment.

**Figure 10:**
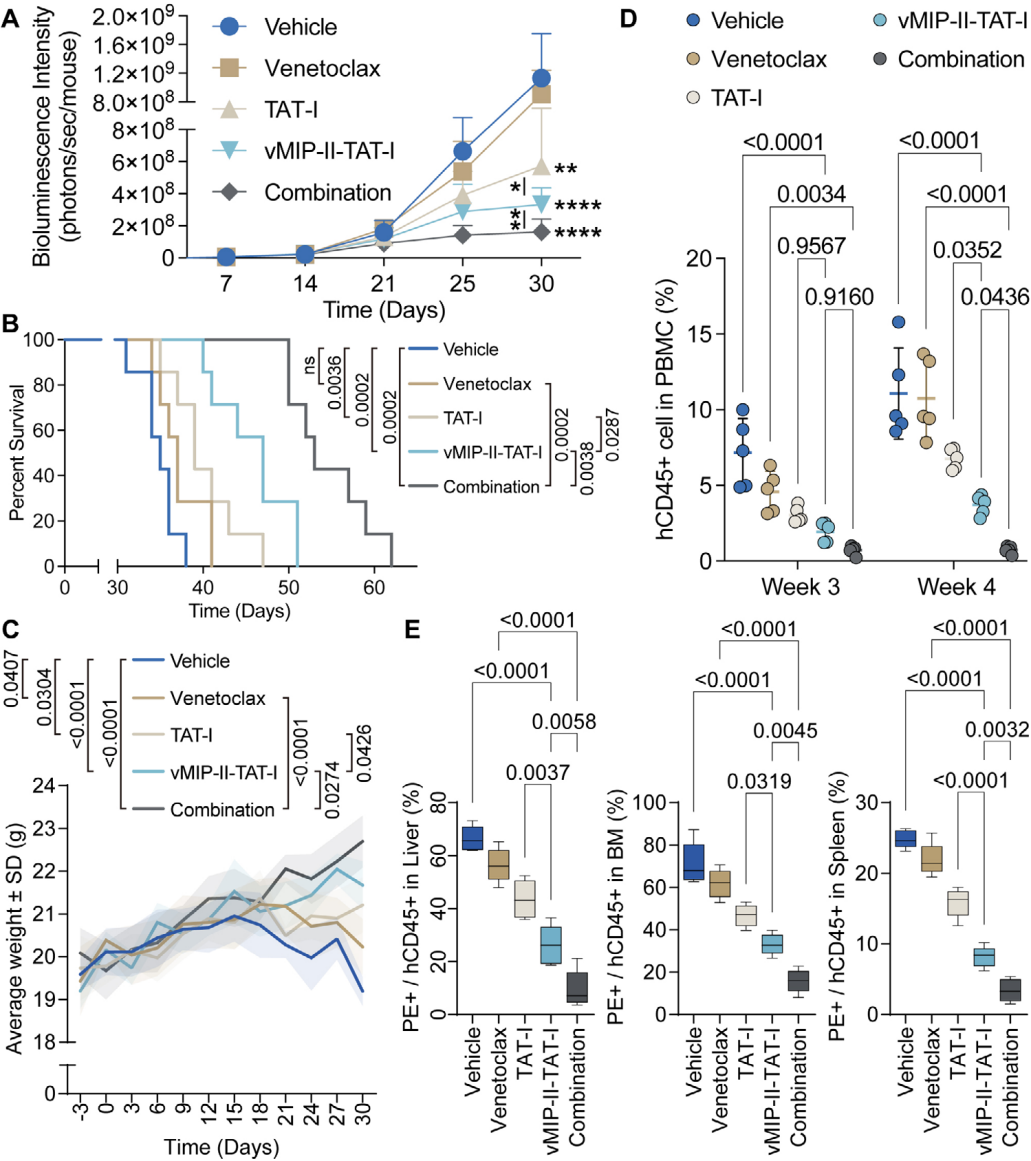
Synergistic tumor suppression, survival benefit, and leukemia burden reduction by vMIP‐II‐TAT‐I and Venetoclax in an in vivo AML model. A) Longitudinal BLI tracking leukemia progression across treatment groups (Vehicle, Venetoclax, TAT‐I, vMIP‐II‐TAT‐I, and Combination), illustrating tumor burden dynamics over time (*n* = 5). B) Kaplan–Meier survival analysis of mice receiving the indicated treatments, demonstrating differential survival benefits (*n* = 7). C) Body weight monitoring across treatment groups, providing insights into treatment‐related physiological effects (*n* = 12). Shaded areas represent the standard deviation (± SD) around the mean. D) Human CD45+ cell burden in peripheral blood mononuclear cells (PBMCs) at weeks 3 and 4, reflecting leukemia progression and treatment efficacy (*n* = 5). E) Quantification of human CD45+ cells in the spleen, bone marrow (BM), and liver at the study endpoint, highlighting differences in leukemic infiltration among treatment groups (*n* = 5). Error bars represent mean ± SD. Statistical analysis was performed using two‐way ANOVA for (A,C,D), log‐rank (Mantel‐Cox) test for (B), and one‐way ANOVA for (E). Significance is indicated as follows: *p* < 0.05 (*), *p* < 0.01 (**), *p* < 0.001 (***), *p* < 0.0001 (****); ns, not significant.

BLI was conducted over 30 days to monitor tumor progression across treatment cohorts (**Figure** **10**A). The Vehicle group showed continuous bioluminescence increase, indicating unrestrained tumor growth. The Venetoclax group exhibited a similar trend, with minimal therapeutic efficacy. TAT‐I treatment led to a modest bioluminescence reduction compared to Vehicle (*p* < 0.01), but insufficient for significant antitumor activity. In contrast, the vMIP‐II‐TAT‐I group displayed a more pronounced bioluminescence decline (*p* < 0.05 vs TAT‐I), particularly after day 25, consistent with tumor suppression. The Combination group showed the most significant bioluminescence reduction, significantly lower than vMIP‐II‐TAT‐I alone (*p* < 0.01), with sustained tumor burden suppression, indicating enhanced therapeutic efficacy beyond monotherapies. This trend was further corroborated by survival analysis (Figure 10B). The Vehicle group displayed the shortest median survival (35 days), with all mice succumbing within 38 days. Venetoclax and TAT‐I treatments extended median survival to 37 and 39 days, respectively. In contrast, vMIP‐II‐TAT‐I treatment significantly prolonged median survival to 47 days, while the Combination group showed the most pronounced survival advantage, with a median survival of 53 days and a subset of mice surviving beyond 60 days, underscoring the synergistic efficacy of the combined therapy in prolonging survival.

Body weight monitoring over 30 days (Figure 10C) showed significant differences among groups. At study end, the Vehicle group had the lowest average weight (≈19 g), while the Combination group exhibited the highest weight gain (≈22.5 g), indicating better overall health. Mice treated with Venetoclax showed slight weight gain (≈20 g), suggesting limited benefit. TAT‐I and vMIP‐II‐TAT‐I treatments resulted in moderate weight increases (≈20.5 g and ≈21.5 g, respectively), but were still lower than the Combination group. The superior weight gain in the Combination group implies that dual therapy enhances both tumor inhibition and overall physiological well‐being, supporting its potential as an optimal therapeutic strategy for AML.

Flow cytometry analysis of hCD45+ cells in PBMCs at weeks 3 and 4 revealed significant differences among treatment groups (Figure 10D; Figure S12A, Supporting Information). At week 3, the Vehicle group had the highest percentage of hCD45+ cells (≈7%), followed by Venetoclax (≈5%), TAT‐I (≈3%), and vMIP‐II‐TAT‐I (≈2%), with the Combination therapy showing a significant reduction (≈1%) compared to Venetoclax. By week 4, the Combination therapy demonstrated a marked decrease (≈1%) compared to Venetoclax and vMIP‐II‐TAT‐I. The hCD45+ population in the spleen, BM, and liver was also analyzed (Figure 10E; Figure S12B, Supporting Information). In the spleen, the Vehicle group had the highest percentage of PE+/hCD45+ cells (≈25%), with the Combination therapy achieving the lowest percentage (≈3%) and showing significant differences (for most comparisons). In the BM, the Vehicle group had the highest percentage (≈70%), with the Combination therapy significantly lowering it to ≈15%. In the liver, the Vehicle group had the highest percentage (≈65%), with the Combination therapy showing the lowest percentage (≈10%) and significant differences. These results confirm the superior efficacy of the Combination therapy in targeting leukemia cells, suggesting its potential to disrupt survival pathways more effectively than monotherapies.

**Figure 11:**
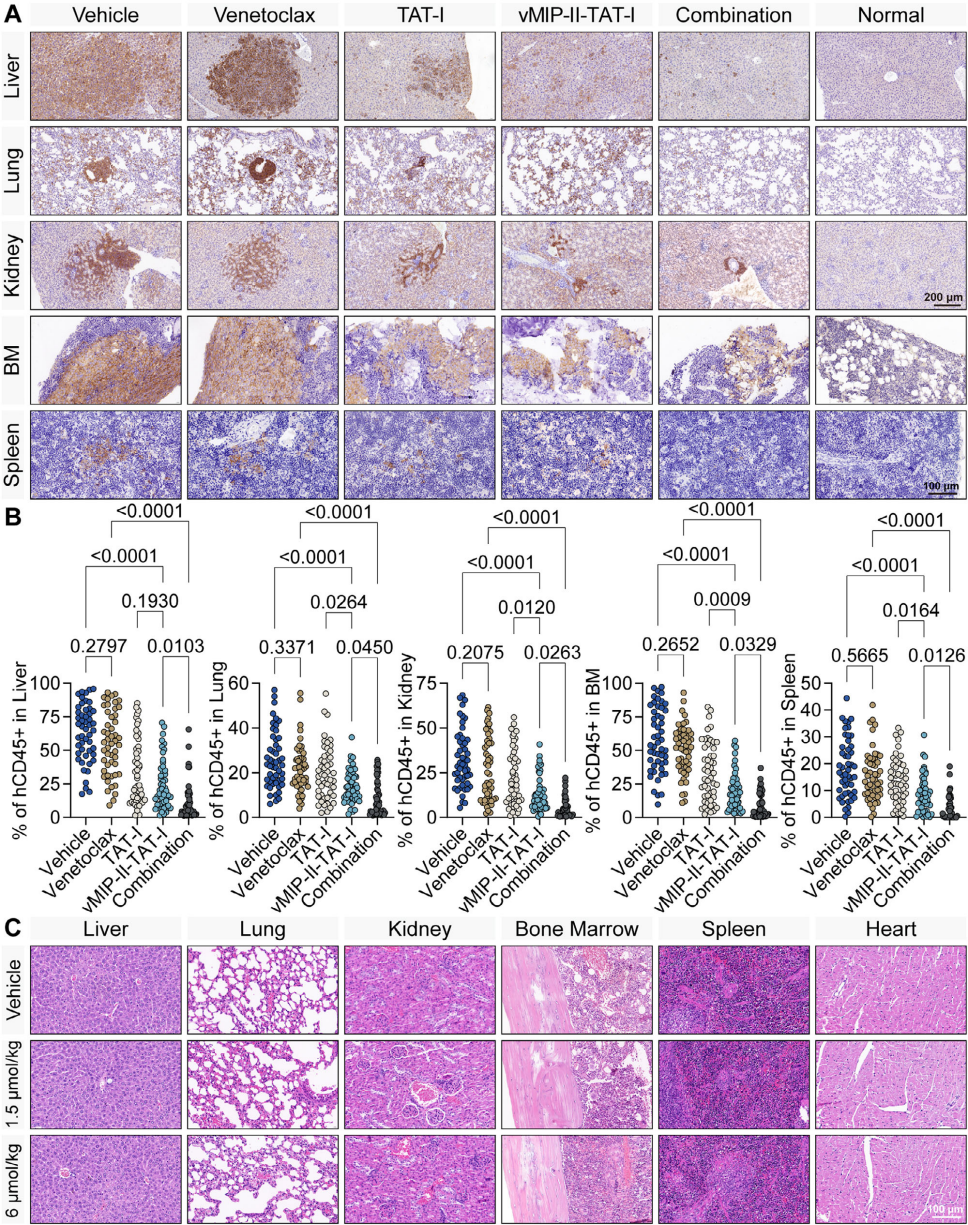
Histopathologic and immunohistochemical analysis of organ infiltration by hCD45+ cells and organ toxicity. A) Immunohistochemical staining of liver, lung, kidney, BM, and spleen sections, assessing hCD45+ cell infiltration under different treatment conditions (Vehicle, Venetoclax, TAT‐I, vMIP‐II‐TAT‐I, Combination, and Normal). Scale bars: 200 µm (liver, lung, kidney); 100 µm (BM, spleen). B) Quantitative analysis of hCD45+ cell infiltration across treatment groups, demonstrating the extent of leukemic burden in each organ (*n* = 50). C) Hematoxylin and eosin (H&E) staining of liver, lung, kidney, BM, spleen, and heart sections, evaluating dose‐dependent histological changes following vMIP‐II‐TAT‐I treatment (Vehicle, 1.5 µmol kg^−1^, 6 µmol kg^−1^). Scale bar: 100 µm. Statistical analysis in (B) was performed using the Shapiro–Wilk test for normality, followed by the Kruskal‐Wallis test and Dunn's post hoc test. *p* < 0.05 was considered statistically significant.

To evaluate the impact of Vehicle, Venetoclax, TAT‐I, vMIP‐II‐TAT‐I, and Combination therapy on leukemic cell infiltration, histopathological and immunohistochemical (IHC) analyses were performed on major organs, including the liver, lung, kidney, BM, spleen, and heart (**Figure** **11**A). In the Vehicle group, extensive hCD45+ cell infiltration was observed, indicating widespread leukemic burden. Venetoclax alone led to a modest reduction in leukemic infiltration, while TAT‐I and vMIP‐II‐TAT‐I resulted in more pronounced decreases. Notably, Combination therapy elicited the most substantial reduction, with hCD45+ cell levels approaching those observed in normal tissue. A similar trend was observed across the lung, kidney, BM, and spleen, where Combination therapy consistently yielded the lowest leukemic burden.

Quantitative analysis of hCD45+ cell percentages (Figure 11B) confirmed that Combination therapy significantly reduced leukemic infiltration to 5–10% across multiple organs, markedly lower than in the Vehicle and monotherapy groups. In contrast, Venetoclax alone exhibited limited efficacy, with hCD45+ cell levels ranging from 13% to 55%, whereas TAT‐I and vMIP‐II‐TAT‐I independently achieved moderate reductions (10–34%). Despite these individual effects, only Combination therapy consistently suppressed hCD45+ cell infiltration below 10% across all organs, underscoring its superior anti‐leukemic efficacy. Histopathological examination using H&E staining showed no significant differences in tissue structure or cellular morphology among groups, even at higher doses of vMIP‐II‐TAT‐I (6 µmol kg^−1^), indicating the safety and lack of toxicity of the polypeptide drug in vivo (Figure 11C), demonstrating that vMIP‐II‐TAT‐I is well‐tolerated in vivo and exhibits no overt toxicity.

Complete blood count (CBC) results (Table S2, Supporting Information) show that vMIP‐II‐TAT‐I administered at 1.5 µmol kg^−1^ (10 mg kg^−1^) and 6 µmol kg^−1^ (30 mg kg^−1^) does not induce significant hematological toxicity, with the majority of parameters remaining within reference ranges. This indicates a favorable safety profile and suggests that the vMIP‐II‐TAT‐I molecular design optimizes cellular uptake while minimizing toxic side effects. vMIP‐II‐TAT‐I targets CXCR4‐dependent pathways in AML cells, exploiting differences in the microenvironment and signaling between diseased and healthy cells. Combined with Venetoclax, vMIP‐II‐TAT‐I enhances leukemic cell death, significantly reducing CD45+ cells. Collectively, this combination therapy provides superior tumor growth inhibition, reduces leukemic cell infiltration in vital organs, prevents metastasis, and improves survival outcomes in AML models compared to monotherapies.

## 3 Conclusion 

In conclusion, our study presents a modular approach to designing BH3 mimetics for AML, leveraging ncAAs and targeted motif engineering to achieve dual inhibition of MCL‐1 and BCL‐xL, effectively addressing Venetoclax‐associated resistance mechanisms. Through site saturation mutagenesis scanning, the I58(Chg) mutation was identified, yielding IC_50_ values of 2.77 nm for MCL‐1 and 10.69 nm for BCL‐xL, representing an increase in binding affinity of fourfold or more compared to BimBH3. The engineered vMIP‐II‐TAT‐I peptide demonstrated superior cellular uptake, exhibiting a 7.2‐fold higher MFI in CXCR4‐positive AML cells compared to non‐targeted controls. Additionally, vMIP‐II‐TAT‐I showed higher selectivity for AML cells, achieving a greater SI than AT‐101, a known MCL‐1/BCL‐xL inhibitor. Notably, minimal off‐target effects were observed in HSCs, underscoring its favorable safety profile. The combination of vMIP‐II‐TAT‐I with Venetoclax induced synergistic apoptosis, with apoptosis rates reaching 92.8% in THP‐1 cells and 90.5% in U‐937 cells. In vivo studies further confirmed its therapeutic potential, demonstrating significant tumor burden reduction and a median survival extension to 47 days, compared to 37 days with Venetoclax monotherapy, highlighting the efficacy of dual inhibition in overcoming adaptive resistance. Importantly, this dual‐targeting strategy successfully neutralized compensatory upregulation of MCL‐1 and BCL‐xL, a major resistance mechanism limiting the efficacy of BCL‐2 inhibitors. These findings demonstrate that vMIP‐II‐TAT‐I selectively targets AML cells with high affinity, effectively inducing apoptosis while sparing HSCs. By overcoming resistance associated with BCL‐2 inhibitors, this dual‐targeting approach provides a precise and potent therapeutic strategy, minimizing off‐target toxicity and enhancing treatment efficacy in AML. While these results are promising, further investigations in larger preclinical cohorts and long‐term safety assessments are necessary. Future studies will focus on optimizing peptide stability, expanding in vivo evaluations, and advancing toward clinical trials to assess the therapeutic potential of vMIP‐II‐TAT‐I in combination with Venetoclax for refractory and relapsed AML. Collectively, this work establishes a new paradigm for BH3 mimetic design, providing a next‐generation strategy to enhance specificity, binding affinity, and therapeutic efficacy, ultimately offering new hope for patients with AML.

## Conflict of Interest

The authors declare no conflict of interest.

## Ethics Approval Statement

Ethical approval for the use of human specimens was obtained from the Research Ethics Committee of the Eighth Affiliated Hospital, Sun Yat‐sen University under Certification No. 2025‐009‐01. The animal use protocol was approved by the Institutional Animal Care and Use Committee (IACUC) of Sun Yat‐sen University under Approval No. SYSU‐IACUC‐2024‐000625.

## Supporting information



Supporting Information

## Data Availability

The data that support the findings of this study are available in the supplementary material of this article.
