# Supplementary material for: Targeted Penetrating Motif Engineering of BH3 Mimetic: Harnessing Non‐Canonical Amino Acids for Coinhibition of MCL‐1 and BCL‐xL in Acute Myeloid Leukemia
Source: Adv Sci (Weinh). 2025 Apr 30;12(27):2503682. doi: 10.1002/advs.202503682 (PMC12279208; doi:10.1002/advs.202503682)
Supplement: Supplementary file 1 — Supporting Information [file ADVS-12-2503682-s001.docx]

**Supplementary Information**

Targeted Penetrating Motif Engineering of BH3 Mimetic: Harnessing Non-Canonical Amino Acids for Coinhibition of MCL-1 and BCL-xL in Acute Myeloid Leukemia

Zhe Wang ^1#^, Ruizhi Lai ^1#^, Xinpei Wang ^2#^, Xu Chen ^2^, Youjian Zhou ^1^, Shengbin Li ^2^, Xiaohui Qiu ^2^, Zekai Zeng ^1^, Jianye Yuan ^1^, Jinghuan Mao ^2^, Zhidong Chen ^2^, and Junqing Wang ^2^**^*^**

^1^Department of Pathology, The Eighth Affiliated Hospital, Sun Yat-sen University, Shenzhen 518033, China

^2^School of Pharmaceutical Sciences, Shenzhen Campus of Sun Yat-sen University, Shenzhen 518107, China

Z.W., R.L., and X.W. Contributed equally to this work.

*****Correspondence: wangjunqing@mail.sysu.edu.cn (J.W.)

**Contents**

[Methods 3](#_Toc195382502)

[Database of amino acids 3](#_Toc195382503)

[Protein structure preparation and in silico mutagenesis scanning 3](#_Toc195382504)

[Molecular dynamics simulation and MMPBSA calculation 3](#_Toc195382505)

[Weighted Root Mean Square Deviation analysis 4](#_Toc195382506)

[Materials and reagents 4](#_Toc195382507)

[Polypeptide synthesis 4](#_Toc195382508)

[Cell lines and cell culture 5](#_Toc195382509)

[MCL-1 and BCL-xL Inhibition Assessment 5](#_Toc195382510)

[Cell viability assessment 6](#_Toc195382511)

[LDH release and membrane integrity 6](#_Toc195382512)

[Hemolytic activity evaluation 7](#_Toc195382513)

[Apoptosis detection 7](#_Toc195382514)

[RT-qPCR analysis 7](#_Toc195382515)

[Western blot 8](#_Toc195382516)

[Stability evaluation of peptides in plasma 9](#_Toc195382517)

[Cellular uptake of peptides 9](#_Toc195382518)

[Synergy methods 9](#_Toc195382519)

[Isolation and characterization of CD34+ hematopoietic stem cells 10](#_Toc195382520)

[Selectivity index calculation 10](#_Toc195382521)

[JC-1 mitochondrial membrane potential assay 10](#_Toc195382522)

[Cell luciferase 11](#_Toc195382523)

[In vivo study 11](#_Toc195382524)

[Immunohistochemistry and hematoxylin and eosin staining 12](#_Toc195382525)

[Statistics 13](#_Toc195382526)

[Figure S1. 20](#_Toc195382527)

[Figure S2. 37](#_Toc195382528)

[Figure S3 38](#_Toc195382529)

[Figure S4 39](#_Toc195382530)

[Figure S5 41](#_Toc195382531)

[Figure S6 42](#_Toc195382532)

[Figure S7 43](#_Toc195382533)

[Figure S8 44](#_Toc195382534)

[Figure S9 45](#_Toc195382535)

[Figure S10 46](#_Toc195382536)

[Figure S11 50](#_Toc195382537)

[Figure S12 51](#_Toc195382538)

[Table S1 52](#_Toc195382539)

[Table S2 53](#_Toc195382540)

# Methods

## Database of amino acids

The amino acids database for peptide construction consists of 264 amino acids, categorized into 20 canonical amino acids and 244 ncAAs. The ncAAs include 19 D-enantiomers of the canonical amino acids (except glycine), N-methyl amino acids, synthetic amino acids with artificial side chains, and others. These ncAAs were chosen based on their biological activity and commercial availability. To enhance the precision of side chain placement in peptides, all rotational isomers of the amino acids were analyzed using Molecular Operating Environment (MOE 2022.02, Chemical Computing Group Inc., Montreal, QC, Canada). Additionally, the amino acids were ionized to ensure accurate charge representation. Detailed structural information for the 244 ncAAs is provided in Figure S1.

## Protein structure preparation and in silico mutagenesis scanning

To ensure precise design, high-resolution crystal structures of the tBimBH3 domain in complex with MCL-1 (PDB: 2NL9) and BCL-xL (PDB: 4QVF) were retrieved from the RCSB Protein Data Bank. Protein structure preparation was performed using MOE, which included repairing deletions in the PDB structure and protonating the protein in its default ionization state to optimize the hydrogen bonding network. All water molecules were removed. The protonation states of amino acid residues were determined using the Protonate 3D module in MOE, with physiological conditions set to 300 K, pH 7.0, and 0.1 M salt. This optimization refined the protein’s conformation and ensured stability during subsequent analyses. Single-site saturation mutagenesis scanning was conducted using the Residue Scan module of MOE to explore novel amino acid side chains that enhance peptide interactions with target proteins. AMBER parameters were applied for proteins. The dStability values assessed the mutation's relative thermostability, while the dAffinity values evaluated its relative binding affinity to the wild type [1].

## Molecular dynamics simulation and MMPBSA calculation

Molecular dynamics (MD) simulations were performed using GROMACS 2023.02 [2] with the CHARMM36 force field [3]. Protein-peptide complexes were solvated in TIP3P-modeled water [4] within periodic cubic boundaries set at 1.2 nm. The system was neutralized and adjusted to physiological conditions with 0.145 M sodium chloride. Initial energy minimization was conducted based on the steepest-descent algorithm for up to 5000 steps. The system was then heated to 310 K over 1 ns in the NVT ensemble using the V-rescale thermostat [5], followed by the NPT ensemble to maintain a pressure of 1 bar for 1 ns with the Parrinello-Rahman algorithm [6]. Production simulations were conducted for 500 ns with a 2 fs time step, utilizing the LINCS algorithm [7] to constrain bonds involving hydrogen atoms. Long-range Coulomb interactions were calculated using the Particle Mesh Ewald method [8], with truncation distances for van der Waals and Coulomb interactions set at 1 nm.

The Molecular mechanics/Poisson–Boltzmann surface area (MMPBSA) method is known for its balance between accuracy and computational efficiency. gmx_MMPBSA [9] was used to calculate the binding free energy (ΔGbind) of proteins and peptides for further evaluation, based on 2000 frames averaged extracted from the entire simulation trajectories and was applied throughout this study for all ΔGbind estimations.

## Weighted Root Mean Square Deviation analysis

The Weighted Root Mean Square Deviation (W-RMSD) was calculated to evaluate the conformational stability of peptide-protein complexes during MD simulations[10]. The calculation focused on the simulation interval from 250–500 ns, which represents the equilibrated phase of the simulation, ensuring that the initial stabilization period (0–250 ns) does not influence the results. The formula for W-RMSD calculation is as follows:

$$W-\mathrm{RMSD}=w_{1}\times\text{RMSD (protein)}+w_{2}\times\text{RMSD (peptide)}$$

RMSD (protein) and RMSD (peptide) represent the root mean square deviations of the protein receptor and peptide ligand, respectively, and w₁ = w₂ = 0.5, indicating equal weighting for both components. RMSD values were extracted from periodic snapshots of the MD simulation trajectories performed using GROMACS. Periodic snapshots from 250–500 ns were averaged to derive stable W-RMSD values.

## Materials and reagents

Chemical reagents, including dimethyl sulfoxide (DMSO, >99%), ethanol, hydrochloric acid, and dimethyl benzene, were sourced from Macklin (Shanghai Macklin Biochemical Co., Ltd., Shanghai, China). Ultrapure water was obtained using a Millipore Milli-Q Integral 3 Ultrapure Water System (Billerica, MA, USA). New Zealand rabbit blood was acquired from Guangzhou Hongquan Biotechnology Co., Ltd. (Guangzhou, China). Roswell Park Memorial Institute (RPMI) 1640 medium, fetal bovine serum (FBS), Phosphate buffered saline (PBS), 0.25% trypsin-EDTA (1X), and penicillin-streptomycin solution (penicillin 10,000 units/mL and streptomycin 10,000 µg/mL) were obtained from GIBCO, Invitrogen (Grand Island, USA). Venetoclax (ABT-199, HY-15531) and AT-101 ((R)-(-)-Gossypol, HY-15464)

were procured from MedChemExpress. All reagents were used as received from commercial suppliers without further purification.

## Polypeptide synthesis

Solid-phase synthesis of BH3 variant peptides: BH3 variant peptides were synthesized using Fmoc-based solid-phase peptide synthesis (SPPS) protocols on Rink amide resin[11]. Coupling reactions were conducted with three equivalents of Fmoc-protected amino acids (Fmoc-AA), HATU, and five equivalents of Oxima-Pure, with DIPEA in DMF as the solvent. The reactions were stirred for one hour at room temperature. Following each coupling step, Fmoc deprotection was performed by treating the resin-bound peptide with 20% piperidine in DMF for 10 minutes, repeated twice. Upon completion of the synthesis, the peptides were cleaved from the resin using a cocktail of TFA, TIS, and water (94:3:3, v/v) for three hours at room temperature. The cleaved peptides were precipitated in diethyl ether, centrifuged, and dried under vacuum. Purification was achieved using reverse-phase high-performance liquid chromatography (RP-HPLC) with a Luna C18 column (Phenomenex) and a water/acetonitrile gradient (30–70%) containing 0.1% TFA. The purified peptides, with a purity exceeding 95%, were characterized by mass spectrometry (Waters ZQ2000 single quadrupole MS) and HPLC (Waters Alliance e2695) to confirm their identity and purity.

FITC labeling of BH3 variant peptides: To selectively label the C-terminal lysine side chain of the BH3 variant peptides with fluorescein isothiocyanate (FITC), Fmoc-Lys(Dde)-OH was introduced as the terminal amino acid on CTC resin. The peptide synthesis proceeded as described above. After completing the sequence, the N-terminal amine was deprotected and capped with a Boc group to prevent nonspecific reactions during labeling. The Dde protecting group on the lysine side chain was selectively removed using 4% hydrazine in DMF and applied three times for five minutes each. The free amine on the lysine side chain was then reacted with FITC in DMF containing DIPEA, with the reaction carried out in the dark for 12 hours to avoid photobleaching. The labeled peptides were cleaved from the resin using the TFA/TIS/water cocktail (94:3:3, v/v), followed by RP-HPLC purification under the same conditions as for unlabeled peptides. The FITC-labeled peptides were subsequently characterized by mass spectrometry and HPLC to verify successful labeling and ensure purity. This approach produced high-quality peptides suitable for further applications.

## Cell lines and cell culture

The human acute leukemia cell lines THP-1, U-937, Daudi, and K562 were obtained from the Cell Bank of the Chinese Academy of Sciences (CAS), Shanghai, China. The THP-1 CXCR4 KO cell line was generated using CRISPR-Cas9 by Ubigene Biosciences (Guangzhou, China) with guide sequences GAAGCATGACGGACAAGTAC AGG and CATCCTGGCCTTCATCAGTC TGG. Cells were cultured in RPMI-1640 medium, supplemented with 10% FBS, 10 mmol/L L-glutamine, 100 U/mL penicillin, and 10 mg/mL streptomycin. THP-1 cells stably expressing firefly luciferase (ffLuc) were established via lentiviral transduction, incorporating genes for firefly luciferase and puromycin resistance. These cells were subsequently cultured in complete medium with 1 µg/mL puromycin dihydrochloride (ST551, Beyotime, Shanghai, China) to facilitate bioluminescence (BLI) induction[12].

Cell cultures were maintained in an incubator (Esco Micro Pte. Ltd., Changi, Singapore) under 5% CO₂ and 90% relative humidity at 37°C. All culture operations adhered to the guidelines provided by the American Type Culture Collection (ATCC) to ensure optimal growth conditions. The cells' daily growth status was monitored using a binocular phase contrast microscope, and the medium was refreshed every 2–3 days. Only cells in the logarithmic growth phase were utilized for experimental procedures.

## MCL-1 and BCL-xL Inhibition Assessment

The inhibition of MCL-1 and BCL-xL binding to their respective ligands was quantified using time-resolved fluorescence resonance energy transfer (TR-FRET) assay kits (BPS Biosciences, Catalog Nos. 79506 and 50223). The assay protocol involved preparing samples containing a terbium-labeled donor, a dye-labeled acceptor, the MCL-1/BCL-xL protein, and the corresponding peptide ligand. All samples and controls were run in quadruplicate, ensuring a final DMSO concentration below 0.1%. Selected hit compounds were tested at various concentrations: 0.25, 0.5, 1, 2, 4, 8, 16, 32, 64, 125, 500, and 1000 nM. The test assay components were prepared as follows: A 3× MCL TR-FRET assay buffer was diluted with two parts distilled water to create a 1× MCL TR-FRET assay buffer. The anti-His terbium-labeled donor and dye-labeled acceptor were diluted 200-fold in 1× MCL TR-FRET assay buffer. Each well received 5 μL of the anti-His terbium-labeled donor and dye-labeled acceptor mixture. In the negative control wells, 2 μL of inhibitor buffer and 1× MCL-1 TR-FRET assay buffer were added. For the positive control wells, 2 μL of inhibitor buffer and the MCL-1 peptide ligand were added. Subsequently, 2 μL of the diluted test inhibitor solution and the MCL-1 peptide ligand were added to the test inhibitor wells. Finally, 3 μL of diluted MCL-1 protein solution was added to each well, and the plate was incubated at room temperature for 3 hours[13].

$$\text{TR-FRET Ratio}=\frac{\text{Emission at 665 nm}}{\text{Emission at 620 nm}}$$

The percent activity was calculated as follows:

$$\text{Percent Activity}=\left( \frac{\text{FRET}_{\text{sample}}-\text{FRET}_{\text{neg}}}{\text{FRET}_{\text{p}}-\text{FRET}_{\text{neg}}} \right)\times100\%$$

where FRET_sample_ is the sample FRET, FRET_neg_ is the negative control FRET, and FRET_p_ is the positive control FRET.

## Cell viability assessment

Equal numbers of viable cells were seeded into 96-well plates at a density of 1×10^4^ cells per well. The cells were incubated with 100 μL of media containing various concentrations of peptide-drug, each supplemented with 0.1% DMSO as the vehicle control. Drug treatments were standardized to achieve a final DMSO concentration of 0.1% across all samples. After a 48-hour incubation period, cell viability was assessed using the Cell Counting Kit-8 (CCK-8) assay (DOJINDO, CK04, Japan) in accordance with the manufacturer's protocol[14]. The optical density (OD) at 450 nm was measured using a microplate reader (Multileader Infinite M200, TECAN, ZH, Switzerland).

## LDH release and membrane integrity

The lactate dehydrogenase (LDH) Cytotoxicity Assay Kit (Beyotime Biotech, C0017) was employed to determine LDH release and assess cell membrane integrity[15]. The assay was conducted following the manufacturer's protocol. Briefly, 1×10^4^ cells per well were seeded into a 96-well plate and treated with the peptide at various concentrations for 48 hours. 25 μL of the LDH leakage reagent was added to one designated well to establish the maximum enzyme activity control. After a 1-hour incubation, the plate was centrifuged at 400g for 5 minutes. Subsequently, 120 μL of the supernatant was transferred to a new 96-well plate. Following the addition of 60 μL of the reaction solution, the plate was incubated for 30 minutes at 37°C. Absorbance was measured at 490 nm and 600 nm wavelengths using a microplate reader (Bio Tek Epoch2, USA).

## Hemolytic activity evaluation

An initial volume of one milliliter of sterile PBS was combined with an equivalent volume of New Zealand rabbit blood. The red blood cells were then isolated by centrifugation at 2500 rpm for 10 minutes. Following centrifugation, the supernatant was removed, and the red blood cells were resuspended in 2 mL of sterile PBS. This process of centrifugation and resuspension was repeated four to five times to ensure the acquisition of a homogenous red blood cell suspension[16]. Subsequently, 300 µL of a 4% erythrocyte suspension was combined with an equal volume of peptide gradient solution to prepare a series of 12 distinct peptide concentrations. Triton X-100 at a concentration of 2% served as a positive control to confirm complete hemolysis. The peptide solutions, or the Triton X-100, were incubated with the erythrocytes at 37°C for three hours. Following the incubation period, the samples underwent final centrifugation at 2500 rpm for 10 minutes. Subsequently, 100 µL of the resulting supernatant from each sample was transferred to a 96-well plate (n = 5)[17]. The hemolysis rate of the red blood cells was quantified by measuring the absorbance at 540 nm using a SpectraMax i3 microplate reader (Molecular Devices, Sunnyvale, CA, USA). The hemolysis rate was calculated using the following formula:

$$\text{Hemolysis Ratio }\left( \% \right)=\left( \frac{OD_{t}-OD_{b}}{OD_{c}-OD_{b}} \right)\times100\%$$

Where OD_t_ is the optical density of the test sample, OD_b_ is the optical density of the blank (PBS without cells), and OD_c_ is the optical density of the positive control (Triton X-100 treated cells).

## Apoptosis detection

Cell apoptosis was measured following the manufacturer's instructions for the Annexin V-FITC kit (Elabscience, E-CK-A211-100). 1 × 10⁵ cells were co-cultured with varying concentrations of peptides or Venetoclax in 24-well plates for 48 hours. After incubation, the cells were collected by centrifugation, washed with PBS, and incubated with Annexin V-FITC and PI staining solution for 15 minutes under light-protected conditions. Apoptosis was then identified and analyzed using a flow cytometer (Beckman CytoFLEX, USA)[18].

## RT-qPCR analysis

Total RNA was isolated from cells using Trizol reagent (Takara, Dalian, China) according to the manufacturer’s instructions. The integrity and purity of the RNA were assessed by measuring the absorbance at 260 and 280 nm using a Nanodrop spectrophotometer (Nanodrop, Wilmington, DE, USA), and RNA quality was confirmed via denaturing agarose gel electrophoresis. Reverse transcription (RT) was performed using the PrimeScript RT Reagent Kit (Takara, Dalian, China) in a total reaction volume of 20 μL, containing 2 μg of total RNA. The resulting complementary DNA (cDNA) was then amplified by PCR.

For PCR amplification, gene-specific primers for CXCR4 (forward: 5'-TGGCTGAAAAGGTGGTCTAG-3', reverse: 5'-CGATGCTGATCCAACTGATGT-3') and GAPDH (forward: 5'-TCATGGGTGGAACCATGAG-3', reverse: 5'-GGATGGGTGAACCTGAGAG-3') were designed and synthesized (Sango, Shanghai, China). PCR was performed using the 2× Taq Master Mix (Vazyme, P111-1) and a total reaction volume of 50 μL. PCR conditions were as follows: initial denaturation at 95°C for 3 min, followed by 30 cycles of denaturation at 95°C for 15 sec, annealing at 60°C for 15 sec, and extension at 72°C for 1 min. Gene expression levels were quantified using the comparative CT method[19], where CT values were normalized to GAPDH, and relative expression was calculated using the formula 2^−ΔΔCT^.

## Western blot

To conduct Western blotting (WB) analysis, the cells were collected by centrifugation following an incubation period in which they were treated with drugs in a mixture containing 1% phenyl methane sulfonyl fluoride (PMSF) (Beyotime, ST506, China), 2% protease and phosphatase inhibitor cocktail (Beyotime, P1045, China), and RIPA lysis buffer (Beyotime, P0013B, China) diluted with PBS. The cells were lysed on ice for 15 minutes, and the total protein was extracted by centrifugation at 13,000 rpm for 10 minutes at 4°C. The protein concentration of the collected supernatant was measured using an Enhanced BCA Protein Assay Kit (Beyotime, P0010, China). SDS-PAGE protein loading buffer (Beyotime, P0015, China) was added, and the solution was heated in a water bath at 100°C for 10 minutes. Subsequently, the proteins were separated on a 10% PAGE gel (EpiZyme, PG212, China) and transferred to 0.22 μm PVDF membranes (Merck Millipore, Cat. No. ISEQ00010). The membranes were blocked for 15 minutes in protein-free rapid blocking buffer (EpiZyme, PS108P, China) and incubated overnight at 4°C with the following primary antibodies: β-tubulin (1:1000, 10068-1-AP, Proteintech, China); caspase-3 and cleaved caspase-3 (1:1000, #9662, Cell Signaling Technology, USA); PARP-1 and cleaved PARP-1 (1:500, sc-8007, Santa Cruz Biotechnology, USA); BCL-2 (1:500, sc-7382, Santa Cruz Biotechnology, USA); MCL-1 (1:1000, YT2679, Immunoway, USA); BCL-xL (1:1000, YN5666, Immunoway, USA); and BAX (1:500, sc-20067, Cell Signaling Technology, USA), BAK (1:1000, YT0449, Immunoway, USA), CXCR4 (1:100, ab124824, Abcam, UK). The bands were washed three times with TBST (G2150-1L, Servicebio, Wuhan, China) for 10 minutes per wash and incubated with secondary antibodies. The membranes were incubated with either anti-mouse IgG secondary antibody (1:5000, SA00001-1, Proteintech, China) or anti-rabbit IgG secondary antibody (1:5000, SA00001-2, Proteintech, China) for one hour. The bands were visualized using an enhanced chemiluminescence reagent (P10300, NCM Biotech, Suzhou, China), and the blotting results were analyzed using the imaging system (baygene BG-gdsAUTO 730) to obtain band images. After testing, the PVDF membrane was incubated in primary and secondary antibody removal solution (Beyotime, Cat#P0025) for 20 minutes to strip and re-blot with the next primary antibody[20]. The final analysis was conducted using ImageJ software (version 1.38e, National Institutes of Health, Bethesda, MD, USA)[21].

**Stability evaluation of peptides in plasma**

Fresh blood was collected from BALB/c mice and centrifuged at 4°C, 4,000 rpm for 5 minutes to separate plasma. The plasma was diluted 10-fold with physiological saline, and a peptide working solution at 2 µg/µL was prepared. Diluted plasma and peptide solution were mixed at a 1:1 ratio and incubated at 37°C. Aliquots were collected at predetermined time points. Each aliquot was mixed with an equal volume of pre-chilled acetonitrile (4°C) to precipitate plasma proteins and centrifuged at 4°C, 12,000 rpm for 10 minutes. The supernatant was mixed with 5× SDS-PAGE loading buffer (Cat# P0285-15ml, Beyotime, Shanghai) and heated at 95°C for 5 minutes to denature proteins. Samples were analyzed using 15% SDS-PAGE (Cat# PG224, Epizyme, Shanghai), loading 1.2 µg protein per lane. A low molecular weight tricolor pre-stained protein ladder (3-40 kDa, Cat# WJ401, Epizyme, Shanghai, China) was used as a molecular weight marker. After electrophoresis, gels were stained overnight with Coomassie Blue Fast Staining Solution (Cat# PS111, Epizyme, Shanghai) and imaged the next day using a UVitec Essential V6 gel imaging system (UVitec, France). All experiments were performed in triplicate to ensure reproducibility.

## Cellular uptake of peptides

5×10⁵ cells were seeded into each well of a 24-well plate. The cells were briefly exposed to FITC-labeled peptide drugs at a low concentration (1 μM) for less than 5 seconds. Immediately after, the wells were rinsed with excess PBS to remove non-internalized peptides, and the cells were collected by centrifugation. The fluorescence intensity was measured using a CytoFLEX flow cytometer (Beckman Coulter, USA). Flow cytometry gating strategies were carefully determined based on at least two of the following controls: untreated cells (PBS control), free FITC (FITC control), non-FITC-labeled peptide derivatives (specificity control), and FITC-labeled peptide derivatives[22]. To visualize the cell nuclei, Hoechst33342 (C1026, Beyotime, China) was used as a nuclear stain, while CXCR4 localization was determined using a PE-conjugated mouse anti-human CXCR4 antibody (Clone HI30, BD Biosciences, Cat. No. 555483). Confocal images were captured using a Zeiss LSM880 microscope.

## Synergy methods

The effects of vMIP-II-TAT-I, Venetoclax, and their combination on the viability of leukemia THP-1 and U-937 cells were assessed using the zero interaction potential (ZIP) synergy model with SynergyFinderPlus, which integrates the principles of both Loewe additivity and Bliss independence models, offering a robust approach to quantify drug interactions [23]. The experimental data were formatted into data files according to the specified requirements and then visually represented as color-coded heatmaps or matrix plots. These graphical representations depicted the inhibition rate and highlighted instances of synergy, ineffectiveness, or antagonism. In the ZIP synergy model, a synergy score (σ) of less than -10 indicates antagonism, a score between -10 and 10 indicates additivity, and a score greater than 10 indicates synergy[24]. Additionally, the combination index (CI) was calculated using Calcusyn software (version 2.0) to determine the degree of synergy. A CI value of less than 1 signifies synergy, a value of 1 indicates additivity and a value greater than 1 suggests antagonism[25].

## Isolation and characterization of CD34+ hematopoietic stem cells

Ethical approval for the use of human specimens was obtained from the Research Ethics Committee of the Eighth Affiliated Hospital, Sun Yat-sen University under Certification No. 2025-009-01. Umbilical cord blood samples were collected from healthy full-term newborns at the Eighth Affiliated Hospital, with informed consent obtained from the donors. The study was approved by the Institutional Review Board (IRB) and adhered to strict ethical guidelines in accordance with the principles of the Declaration of Helsinki. Mononuclear cells were isolated using Ficoll density gradient centrifugation, and CD34⁺ hematopoietic stem cells (HSCs) were enriched using the Miltenyi Biotec CD34 MicroBead Kit (Miltenyi Biotec, Germany). Flow cytometry analysis was performed on a FACSAria II cytometer (BD Biosciences, USA) to assess cell purity, viability, and specific surface marker expression[26]. Antibodies used for staining included CD34-PE/Cy7, CD45-FITC, and PI (propidium iodide) (BioLegend, USA). Data were analyzed using FlowJo software (Tree Star Inc., USA). Cells were cultured in StemPro-34 serum-free medium (Gibco, Thermo Fisher Scientific, USA) supplemented with 30% fetal bovine serum (FBS, HyClone Laboratories, USA), 2 mM L-glutamine, and recombinant human cytokines, including stem cell factor (SCF, 200 ng/mL), Fms-like tyrosine kinase 3 ligand (FLT3L, 200 ng/mL), thrombopoietin (TPO, 100 ng/mL), and interleukin-3 (IL-3, 20 ng/mL) (StemSpan CC100; Stem Cell Technologies, Canada). Cultures were maintained at 37°C in a humidified atmosphere with 5% CO₂.

## Selectivity index calculation

The selectivity index (SI) was calculated to assess the specificity of peptide drugs toward leukemia cells relative to normal human HSCs. The SI is defined as the ratio of the IC_50_ value for HSCs to the IC_50_ value for leukemia cell lines, as shown in the following formula:

$$\mathrm{SI}=\frac{\mathrm{IC}_{50}\left( \mathrm{HSCs} \right)}{\mathrm{IC}_{50}\left( \text{leukemia cells} \right)}$$

The IC_50_ values of peptide drugs were determined in the experiments using the CCK-8 assay after a 24-hour incubation. The human acute leukemia cell line THP-1 was used as the leukemia cell model, while normal HSCs served as the control group. Higher SI values indicate greater selectivity of the peptide for leukemia cells over normal cells[27].

## **JC-1 mitochondrial membrane potential assay**

Enhanced Mitochondrial Membrane Potential Assay Kit JC-1 (Beyotime, C2003S) was used following the manufacturer's instructions. The cells were incubated with JC-1 staining solution for 20 minutes after co-culturing with the drugs. The fluorescence intensity of red and green fluorescence was measured in 10,000 cells using flow cytometry (Beckman CytoFLEX, USA). Confocal laser scanning microscopy (Zeiss LSM880) was used to acquire images[28].

## Cell luciferase

1×10^5^ THP-1 luciferase cells were seeded into each well of a 96-well plate, and luciferase activity was subsequently detected using the Bright-Lumi™ Firefly Luciferase Assay Kit (RG051S, Beyotime). The fluorescence signal was quantified with a microplate reader (Multileader Infinite, Z200ECAN, Switzerland). Prior to inoculating the NCG mice with THP-1-LUC, 24-well plates were prepared with varying cell densities, ranging from 100,000 to 1,000,000,000 cells per well[29]. Each well was then incubated with 20 microliters of 15 mg/ml D-luciferin potassium salt at 37°C for five minutes. The luminescence signals were captured using the IVIS in vivo imaging system (Tanon ABL-X3; Tanon, Shanghai, China) to confirm luciferase expression.

## In vivo study

The animal use protocol was approved by the Institutional Animal Care and Use Committee (IACUC) of Sun Yat-sen University under Approval No. SYSU-IACUC-2024-000625, and all animal care procedures adhered to the guidelines set by the IACUC. The NCG (NOD/ShiLtJGpt-Prkdcem26Cd52Il2rgem26Cd22/Gpt) female mice, aged between six and eight weeks, were obtained from GemPharmatech Co., Ltd (Guangdong, China). All NCG mice were housed in a specific pathogen-free (SPF) animal room with unrestricted access to food and water. To assess the safety of the peptide drug, three healthy, untreated NCG mice were assigned to the Control group (n = 3). Three additional groups (n = 3 each) received normal saline (Vehicle), 1.5 μmol/kg (10 mg/kg), or 6 μmol/kg (30 mg/kg) of the peptide intravenously every two days for four weeks. The hearts, livers, lungs, kidneys, spleens, right femurs, and tibiae were excised and preserved by embedding in paraffin for subsequent hematoxylin and eosin (H&E) staining and histologic analysis. A complete blood count (CBC) was conducted on anticoagulated blood collected from the mice.

To establish the AML mouse model, the animals were intravenously (IV) injected with luciferase-transfected THP-1 cells (THP-1 luciferase; 1 × 10⁶ cells/200 μL). The mice were randomly assigned to one of five experimental groups. Once engraftment had been confirmed, the mice were randomly assigned to one of five groups (n = 12 per group) and treated with either a vehicle, BH3 peptide (1.5 μmol/kg), Venetoclax (25 mg/kg), or a combination of the two, administered intravenously or orally via gavage every other day. Venetoclax was formulated for oral administration in a solution comprising 60% phosal 50 propylene glycol (PG), 30% polyethylene glycol (PEG) 400, and 10% ethanol, provided by MedChemExpress[30]. Before administration, both Venetoclax and BH3 peptide stock solutions were freshly diluted to working concentrations with sterile normal saline. The mice were anesthetized and administered 200 µL of a 15 mg/mL luciferase substrate, D-luciferin potassium salt (Beyotime Biotech, ST196-500mg), intraperitoneally. The progression of AML dissemination in mice was monitored until the day of euthanasia using an in vivo optical imaging system (Tanon ABL-X6; Tanon, Shanghai, China). The results were expressed as total flux (photons/s; radiance photons) ± standard deviation (SD) in both in vivo and ex vivo studies. The extent of the leukemia burden was determined by flow cytometry of peripheral blood human CD45+ cells. During the third and fourth weeks of the study, blood was collected from the orbital sinuses of mice in each group. Erythrocytes were lysed using Red Blood Cell Lysis Buffer (C3702, Beyotime, Shanghai, China) and stained with an hCD45-PE antibody (BD Pharmingen, 555483) prior to flow cytometry analysis. Flow cytometry was employed to ascertain the expression of hCD45 in PBMCs. All mice were euthanized on the day the first animal exhibited pertinent indications of disease, such as the development of a curved back or paralysis of the lower limbs, a lack of mobility, or a 10% weight loss. Following the completion of the designated treatment period, five mice from each group were randomly selected and euthanized by CO₂ asphyxiation. The remaining seven mice in each group were observed for survival. Kaplan-Meier survival curves were generated using GraphPad Prism version 10.1.2. The bone marrow (from the tibia and femur), spleen, and liver were minced and made into single-cell suspensions in PBS, followed by red blood cell lysis. The cells were then stained with hCD45-PE antibody and analyzed by flow cytometry. The heart, liver, lung, kidney, spleen, left femur, and tibia were excised, fixed in 10% formalin, and then embedded in paraffin for further hematoxylin and eosin (H&E) staining and immunohistochemistry (IHC) analysis[31].

## Immunohistochemistry and hematoxylin and eosin staining

Liver, lung, kidney, spleen, and left femur tissues were formalin-fixed and paraffin-embedded. Bone tissue was decalcified with 10% EDTA decalcification solution prior to paraffin embedding. Paraffin blocks were then sectioned at 2-4 μm thickness and mounted on microscope slides. Tissue sections were deparaffinized and rehydrated using 100% xylene and ethanol solutions of decreasing concentrations. The rehydrated tissue sections were subjected to heat-induced epitope retrieval, blocking endogenous peroxidase activity, serocontainment of non-immunized animals (Max Vision KIT-5010, Maixin Biotech, China). They were then incubated with mouse anti-human CD45 monoclonal antibody (D9M8I from CST, Cat# 13917), followed by sequential incubation with biotinylated secondary antibody, peroxidase-labeled streptavidin, and 3,30 diaminobenzidine tetrahydrochloride/H_2_O_2_ (DAB-0031, Maixin Biotech, China), resulting in a brown precipitate at the antigen site. All stained samples were digitized using a Carl Zeiss pannoramic microscope scanner (Pannoramic SCAN, 3DHISTECH Ltd., Budapest, Hungary). In the bottom-up approach used, the entire sample is captured at high resolution and low magnification images are automatically generated. To analyze the potential toxicity of peptide treatments in tissues, hematoxylin and eosin staining were performed in bone, lung, kidney, liver, heart, and spleen. Three independent expert pathologists evaluated possible toxicity in non-leukemic cells of these tissues using the Olympus BX53 microscope (Olympus). Interobserver agreement was achieved in 95% of the samples; the remaining slides were re-evaluated, and consensus decisions were reached[32]. Similarly, immunohistochemical staining was initially evaluated by three independent pathologists to ensure consistency in interpretation. For quantitative analysis, ten random fields per tissue section were selected, and the number of hCD45-positive cells was counted to assess hCD45 protein expression. Positive signals in IHC images were further quantified using the ImageJ IHC Image Analysis Toolbox (National Institutes of Health, Bethesda, USA)[33].

## Statistics

All 3D visualizations were generated using Python (v3.10) with the Matplotlib library (v3.5), and 3D scatter and surface plots were created using the mpl_toolkits.mplot3d module. Statistical analyses were performed using GraphPad Prism (v10.1.2). Unpaired or paired two-tailed Student’s t-tests were used for comparisons between two groups. For multiple group comparisons with normally distributed data, one-way ANOVA followed by Tukey’s post hoc test was applied. If data did not meet normality assumptions (as assessed by the Shapiro–Wilk test), non-parametric Kruskal–Wallis tests followed by Dunn’s post hoc test were used. Two-way ANOVA was used for multi-factorial designs. Correlations were analyzed using Pearson’s or Spearman’s correlation coefficients, with Pearson’s r calculated in Python via scipy.stats.pearsonr. Log-rank (Mantel-Cox) tests assessed survival differences, and hazard ratios were estimated using Cox proportional hazards models. Statistical assumptions such as normality, homogeneity of variance, and sufficient power were evaluated before analysis. All experiments were repeated at least three times. P < 0.05 was considered statistically significant. Significance is indicated as *P < 0.05, **P < 0.01, ***P < 0.001, ****P < 0.0001, and “ns” for not significant.

**References**

[1] M. O. E. (MOE), Chemical Computing Group ULC: 910-1010 Sherbrooke St. W., Montreal, QC H3A 2R7, 2024., **2022.02**; Vol. 2020.

[2] M. J. Abraham, T. Murtola, R. Schulz, S. Páll, J. C. Smith, B. Hess, E. Lindahl, *SoftwareX* **2015**, *1-2*, 19, <https://doi.org/10.1016/j.softx.2015.06.001>.

[3] J. Huang, S. Rauscher, G. Nawrocki, T. Ran, M. Feig, B. L. de Groot, H. Grubmuller, A. D. MacKerell, Jr., *Nat Methods* **2017**, *14* (1), 71, <https://doi.org/10.1038/nmeth.4067>.

[4] W. L. Jorgensen, J. Chandrasekhar, J. D. Madura, R. W. Impey, M. L. Klein, *The Journal of Chemical Physics* **1983**, *79* (2), 926, <https://doi.org/10.1063/1.445869>.

[5] N. Bou-Rabee, Time Integrators for Molecular Dynamics. In *Entropy*, **2014**; Vol. 16, pp 138.

[6] H. Okumura, S. G. Itoh, Y. Okamoto, *J Chem Phys* **2007**, *126* (8), 084103, <https://doi.org/10.1063/1.2434972>.

[7] B. Hess, H. Bekker, H. J. C. Berendsen, J. G. E. M. Fraaije, *Journal of Computational Chemistry* **1997**, *18* (12), 1463, <https://doi.org/10.1002/(sici)1096-987x(199709)18:12><1463::Aid-jcc4>3.0.Co;2-h.

[8] U. Essmann, L. Perera, M. L. Berkowitz, T. Darden, H. Lee, L. G. Pedersen, *The Journal of Chemical Physics* **1995**, *103* (19), 8577, <https://doi.org/10.1063/1.470117>.

[9] a) M. S. Valdes-Tresanco, M. E. Valdes-Tresanco, P. A. Valiente, E. Moreno, *J Chem Theory Comput* **2021**, *17* (10), 6281, <https://doi.org/10.1021/acs.jctc.1c00645>; b) X. Wang, X. Chen, Z. Chen, W. Xu, R. Lai, X. Qiu, Z. Zeng, C. Wang, Z. Wang, J. Wang, *Journal of Chemical Information and Modeling* **2024**, *64* (12), 4739, <https://doi.org/10.1021/acs.jcim.4c00381>.

[10] K. L. Damm, H. A. Carlson, *Biophysical journal* **2006**, *90* (12), 4558.

[11] I. Coin, M. Beyermann, M. Bienert, *Nature protocols* **2007**, *2* (12), 3247.

[12] R. Fazzina, L. Lombardini, L. Mezzanotte, A. Roda, P. Hrelia, A. Pession, R. Tonelli, *Int J Oncol* **2012**, *41* (2), 621, <https://doi.org/10.3892/ijo.2012.1504>.

[13] P. F. Lamie, J. N. J. B. C. Philoppes, **2021**, *116*, 105335.

[14] Z. Chen, S. Shojaee, M. Buchner, H. Geng, J. W. Lee, L. Klemm, B. Titz, T. G. Graeber, E. Park, Y. X. Tan, A. Satterthwaite, E. Paietta, S. P. Hunger, C. L. Willman, A. Melnick, M. L. Loh, J. U. Jung, J. E. Coligan, S. Bolland, T. W. Mak, A. Limnander, H. Jumaa, M. Reth, A. Weiss, C. A. Lowell, M. Muschen, *Nature* **2015**, *521* (7552), 357, <https://doi.org/10.1038/nature14231>.

[15] a) P. Lou, S. Liu, Y. Wang, C. Pan, X. Xu, M. Zhao, G. Liao, G. Yang, Y. Yuan, L. J. A. b. Li, **2021**, *135*, 100; b) K. Shi, J. Li, Z. Cao, P. Yang, Y. Qiu, B. Yang, Y. Wang, Y. Long, Y. Liu, Q. Zhang, J. Qian, Z. Zhang, H. Gao, Q. He, *J Control Release* **2015**, *217*, 138, <https://doi.org/10.1016/j.jconrel.2015.09.009>.

[16] Y. J. Lee, G. Johnson, J. P. Pellois, *Biochemistry* **2010**, *49* (36), 7854, <https://doi.org/10.1021/bi1008408>.

[17] B. He, S. Ma, G. Peng, D. J. N. N. He, Biology, Medicine, **2018**, *14* (2), 365.

[18] a) D. E. Banker, M. Groudine, T. Norwood, F. R. Appelbaum, *Blood* **1997**, *89* (1), 243; b) W. Zhang, M. Konopleva, V. R. Ruvolo, T. McQueen, R. L. Evans, W. G. Bornmann, J. McCubrey, J. Cortes, M. Andreeff, *Leukemia* **2008**, *22* (4), 808, <https://doi.org/10.1038/sj.leu.2405098>.

[19] a) Z. Xiang, Z. Zhou, G. Xia, X. Zhang, Z. Wei, J. Zhu, J. Yu, W. Chen, Y. He, R. Schwarz, *Oncogene* **2017**, *36* (36), 5122; b) L. Sand, K. Scotlandi, D. Berghuis, B. Snaar-Jagalska, P. Picci, T. Schmidt, K. Szuhai, P. Hogendoorn, *European journal of cancer* **2015**, *51* (17), 2624.

[20] E. A. Punnoose, J. D. Leverson, F. Peale, E. R. Boghaert, L. D. Belmont, N. Tan, A. Young, M. Mitten, E. Ingalla, W. C. Darbonne, A. Oleksijew, P. Tapang, P. Yue, J. Oeh, L. Lee, S. Maiga, W. J. Fairbrother, M. Amiot, A. J. Souers, D. Sampath, *Mol Cancer Ther* **2016**, *15* (5), 1132, <https://doi.org/10.1158/1535-7163.MCT-15-0730>.

[21] E. F. Lee, T. J. Harris, S. Tran, M. Evangelista, S. Arulananda, T. John, C. Ramnac, C. Hobbs, H. Zhu, G. Gunasingh, D. Segal, A. Behren, J. Cebon, A. Dobrovic, J. M. Mariadason, A. Strasser, L. Rohrbeck, N. K. Haass, M. J. Herold, W. D. Fairlie, *Cell Death Dis* **2019**, *10* (5), 342, <https://doi.org/10.1038/s41419-019-1568-3>.

[22] S. Vranic, N. Boggetto, V. Contremoulins, S. Mornet, N. Reinhardt, F. Marano, A. Baeza-Squiban, S. Boland, *Particle and fibre toxicology* **2013**, *10*, 1.

[23] B. Yadav, K. Wennerberg, T. Aittokallio, J. Tang, *Comput Struct Biotechnol J* **2015**, *13*, 504, <https://doi.org/10.1016/j.csbj.2015.09.001>.

[24] G. O. Rodriguez-Vazquez, A. O. Diaz-Quinones, N. Chorna, I. K. Salgado-Villanueva, J. Tang, W. I. S. Ortiz, H. M. Maldonado, *Biomed Pharmacother* **2023**, *166*, 115352, <https://doi.org/10.1016/j.biopha.2023.115352>.

[25] A. Latif, A. Newcombe, S. Li, K. Gilroy, N. Robertson, X. Lei, H. Stewart, J. Cole, M. Terradas, L. Rishi, BRD4-mediated repression of p53 is a target for combination therapy in AML. Nat Commun 12: 241. **2021**.

[26] M. F. Mata, D. Hernandez, E. Rologi, D. Grandolfo, E. Hassan, P. Hua, R. Kallmeier, S. Hirani, F. Heuts, V. Tittrea, *Transfusion* **2019**, *59* (12), 3560.

[27] I. Kissin, *Drug Design, Development and Therapy* **2013**, 93.

[28] J. Tang, Y. Li, X. Liu, G. Yu, F. Zheng, Z. Guo, Y. Zhang, W. Shao, S. Wu, H. J. S. o. T. T. E. Li, **2023**, *857*, 159432.

[29] S. Ackler, A. Oleksijew, J. Chen, B. J. Chyla, J. Clarin, K. Foster, T. McGonigal, S. Mishra, S. Schlessinger, M. L. Smith, S. K. Tahir, J. D. Leverson, A. J. Souers, E. R. Boghaert, J. Hickson, *Pharmacol Res Perspect* **2015**, *3* (5), e00178, <https://doi.org/10.1002/prp2.178>.

[30] a) A. J. Souers, J. D. Leverson, E. R. Boghaert, S. L. Ackler, N. D. Catron, J. Chen, B. D. Dayton, H. Ding, S. H. Enschede, W. J. J. N. m. Fairbrother, **2013**, *19* (2), 202; b) S. Peirs, F. Matthijssens, S. Goossens, I. Van de Walle, K. Ruggero, C. E. De Bock, S. Degryse, K. Canté-Barrett, D. Briot, E. J. B. Clappier, The Journal of the American Society of Hematology, **2014**, *124* (25), 3738; c) C. Bi, X. Zhang, T. Lu, X. Zhang, X. Wang, B. Meng, H. Zhang, P. Wang, J. M. Vose, W. C. Chan, T. W. McKeithan, K. Fu, *Haematologica* **2017**, *102* (4), 755, <https://doi.org/10.3324/haematol.2016.159160>.

[31] V. Pallares, Y. Nunez, L. Sanchez-Garcia, A. Falgas, N. Serna, U. Unzueta, A. Gallardo, L. Alba-Castellon, P. Alamo, J. Sierra, A. Villaverde, E. Vazquez, I. Casanova, R. Mangues, *J Control Release* **2021**, *335*, 117, <https://doi.org/10.1016/j.jconrel.2021.05.014>.

[32] X. Xie, W. Yang, W. Zhang, Y. Qiu, Z. Qiu, H. Wang, Y. Hu, Y. Li, X. Zhou, L. Li, Z. Chen, C. Zhao, Y. Lu, K. Zhang, E. Lai, X. Bai, *Biomed Pharmacother* **2022**, *154*, 113566, <https://doi.org/10.1016/j.biopha.2022.113566>.

[33] a) V. Pallares, U. Unzueta, A. Falgas, L. Sanchez-Garcia, N. Serna, A. Gallardo, G. A. Morris, L. Alba-Castellon, P. Alamo, J. Sierra, A. Villaverde, E. Vazquez, I. Casanova, R. Mangues, *J Hematol Oncol* **2020**, *13* (1), 36, <https://doi.org/10.1186/s13045-020-00863-9>; b) K. Young, H. Morrison, *Journal of visualized experiments: JoVE* **2018**, (136), 57648; c) F. Varghese, A. B. Bukhari, R. Malhotra, A. De, *PloS one* **2014**, *9* (5), e96801.


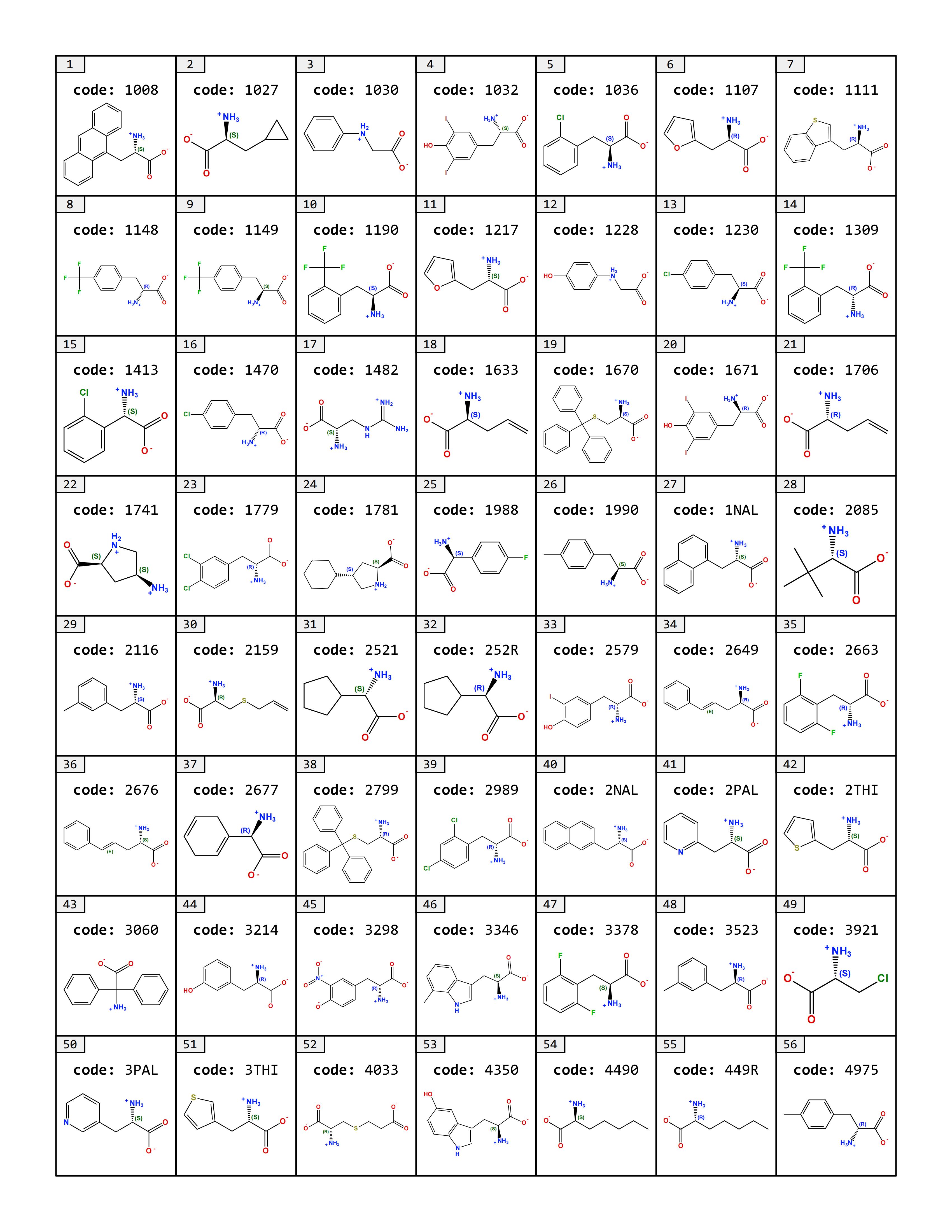


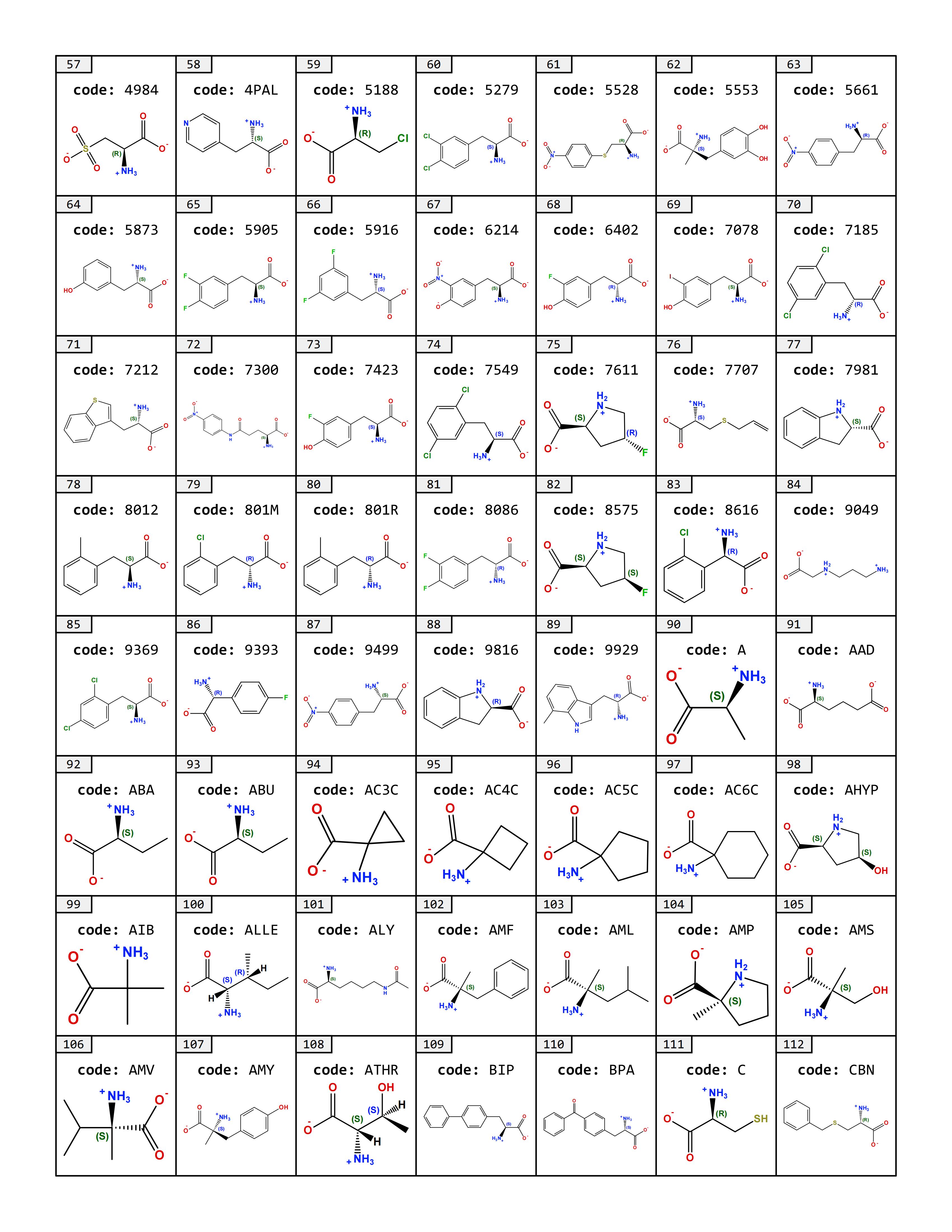


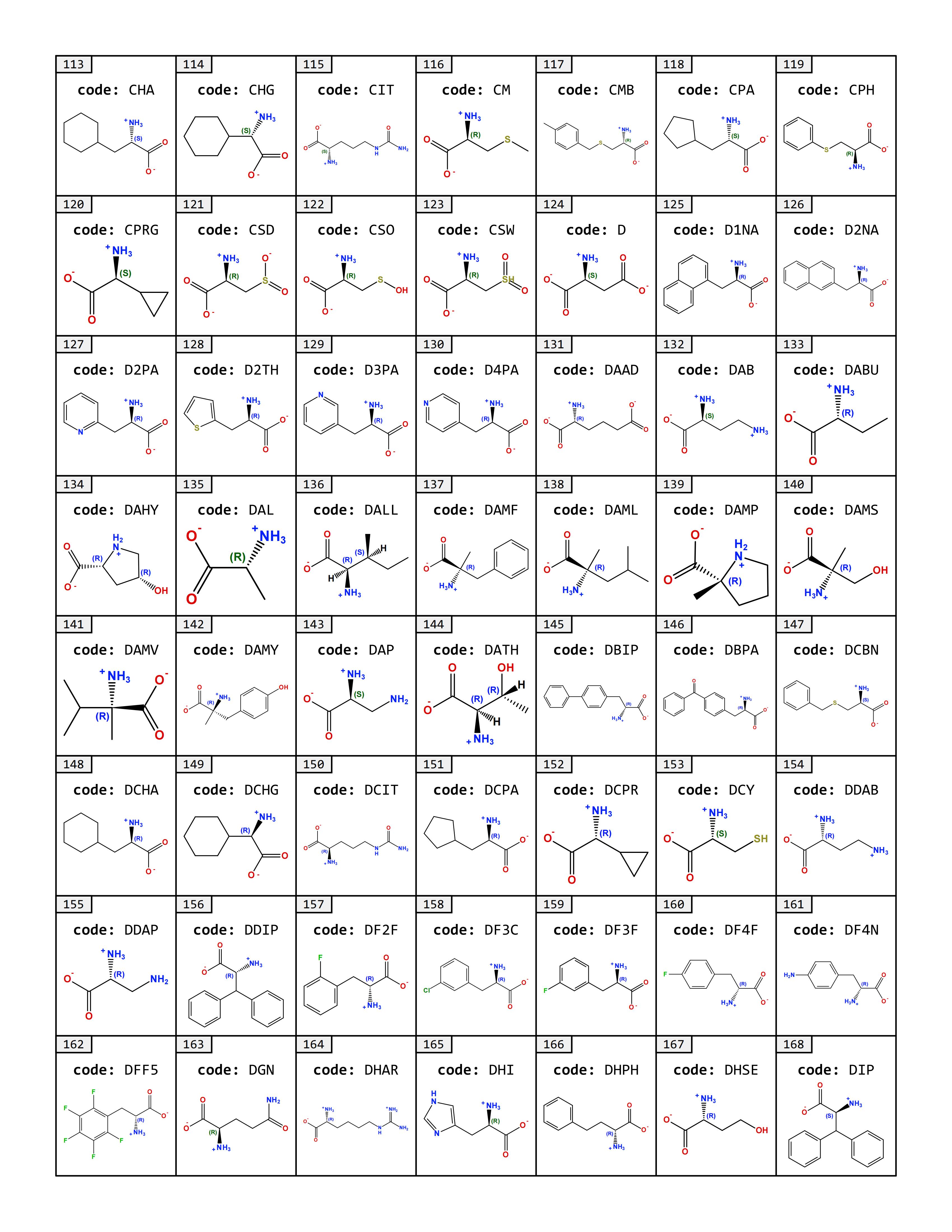


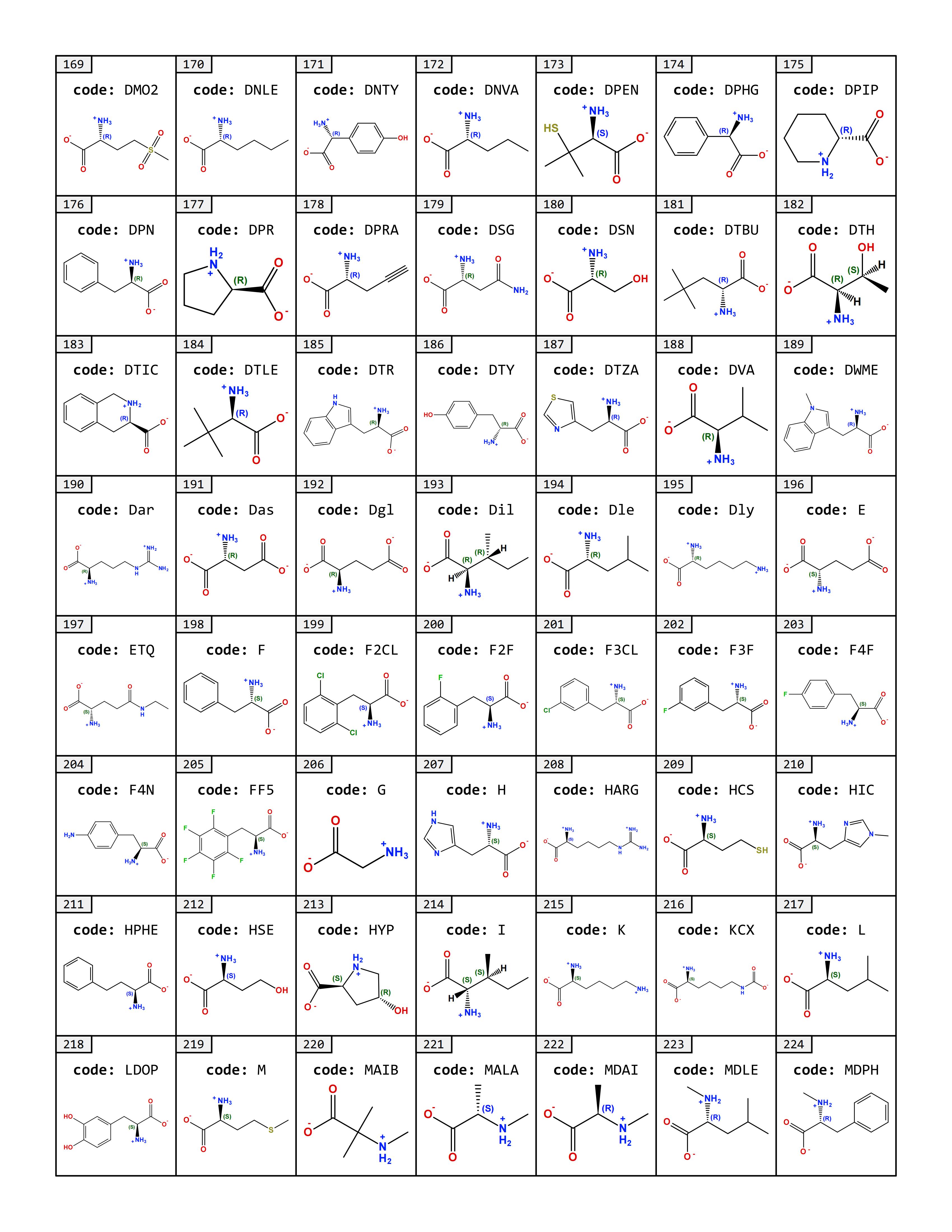


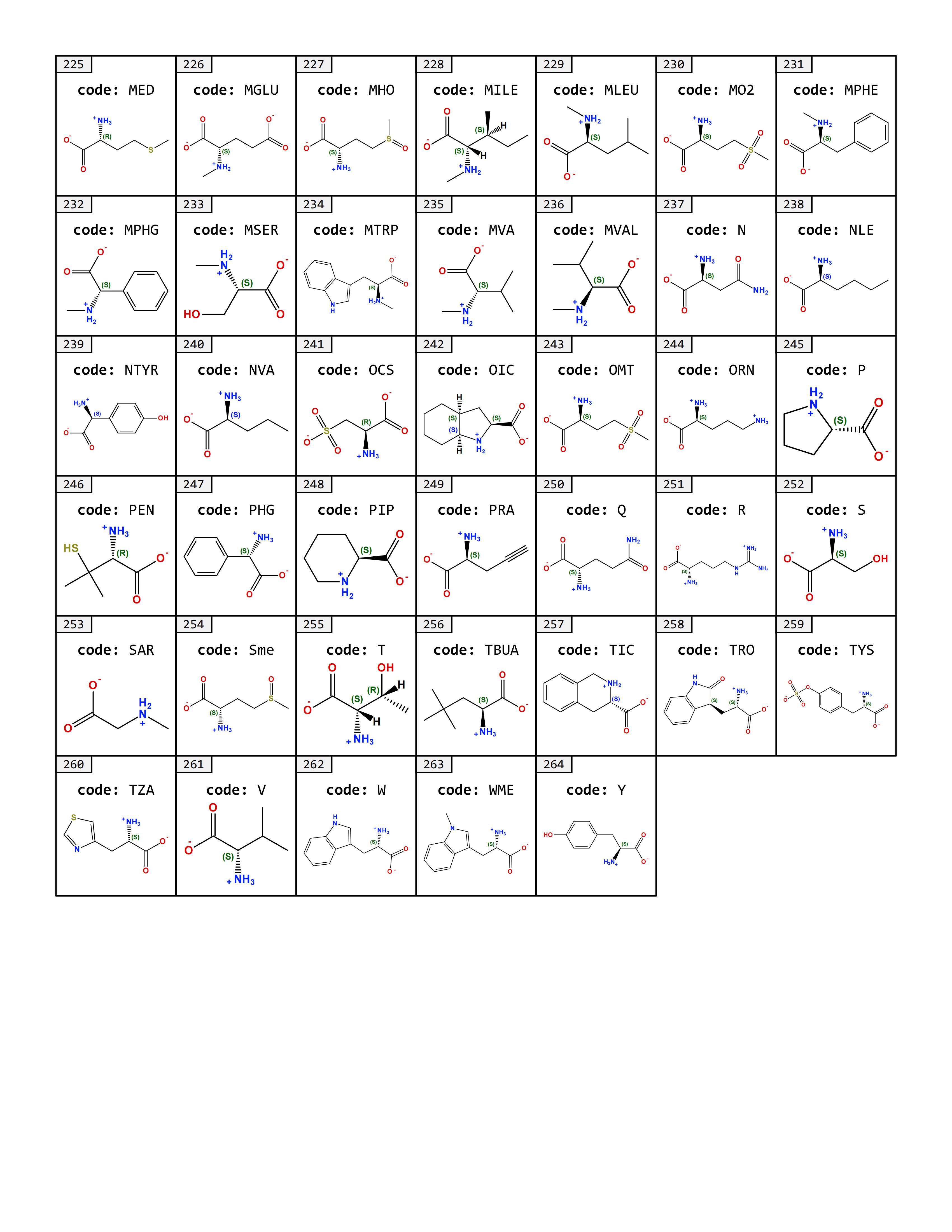


**Figure S1. Chemical structures of commercially available canonical and non-canonical amino acids.**


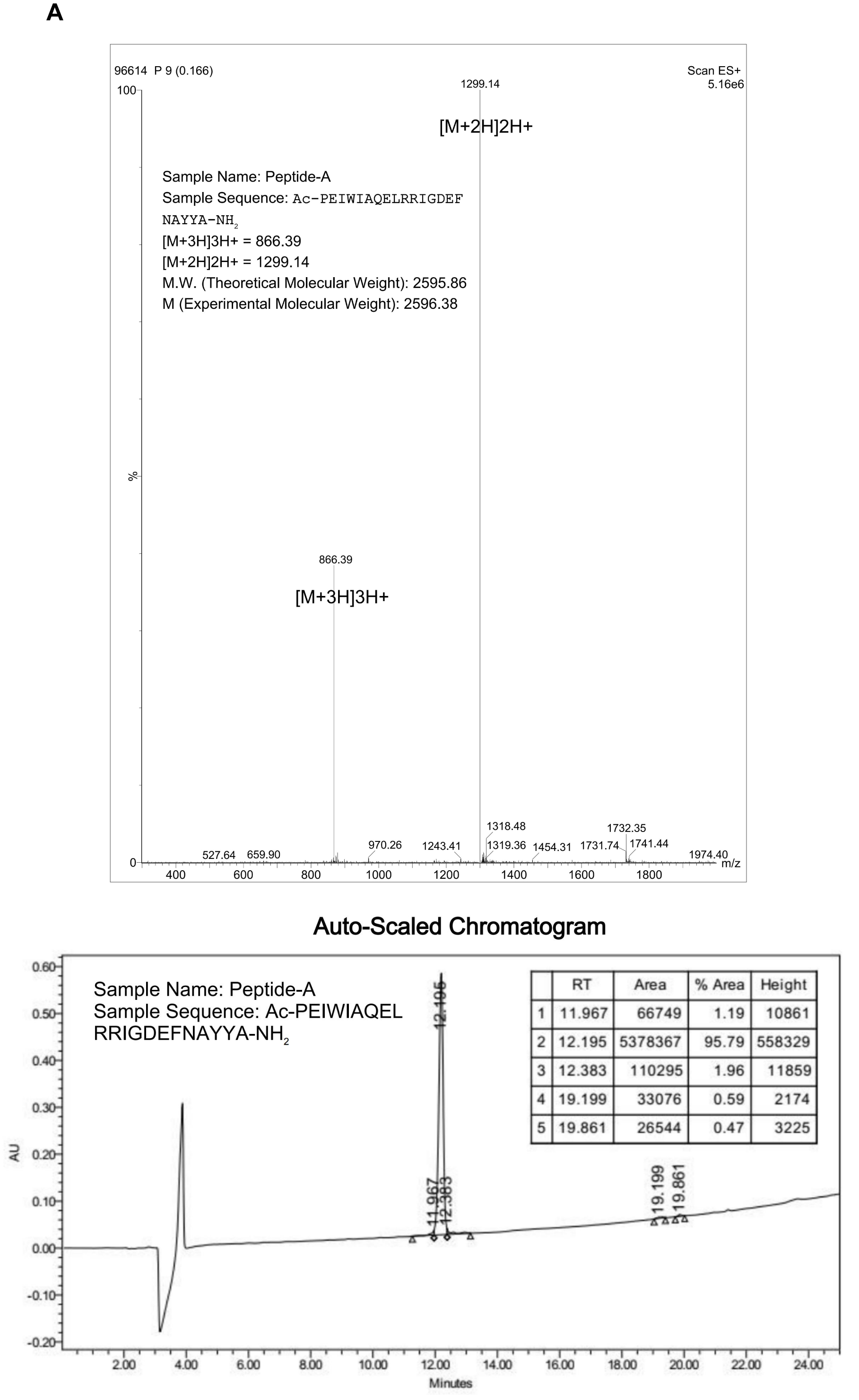


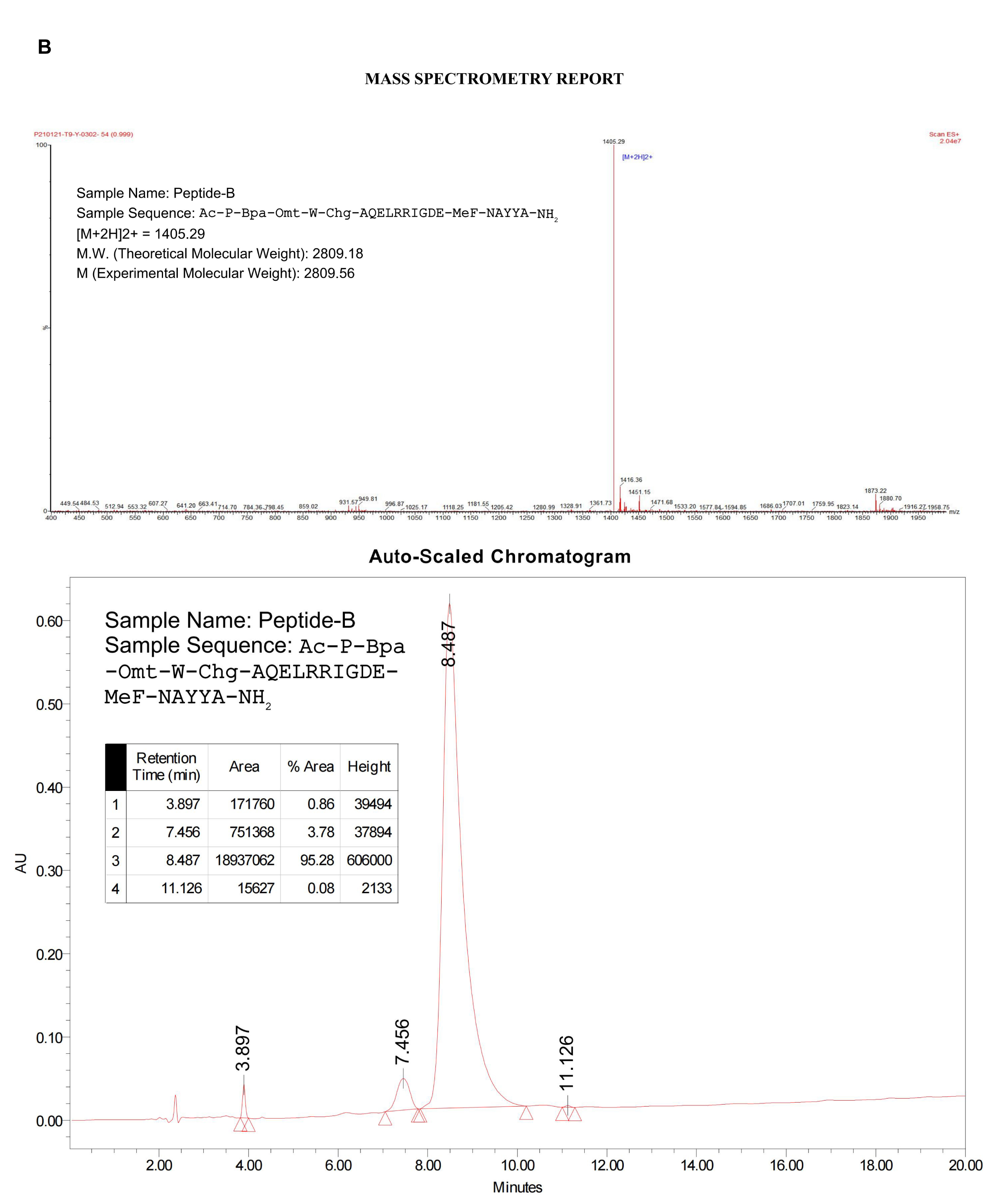


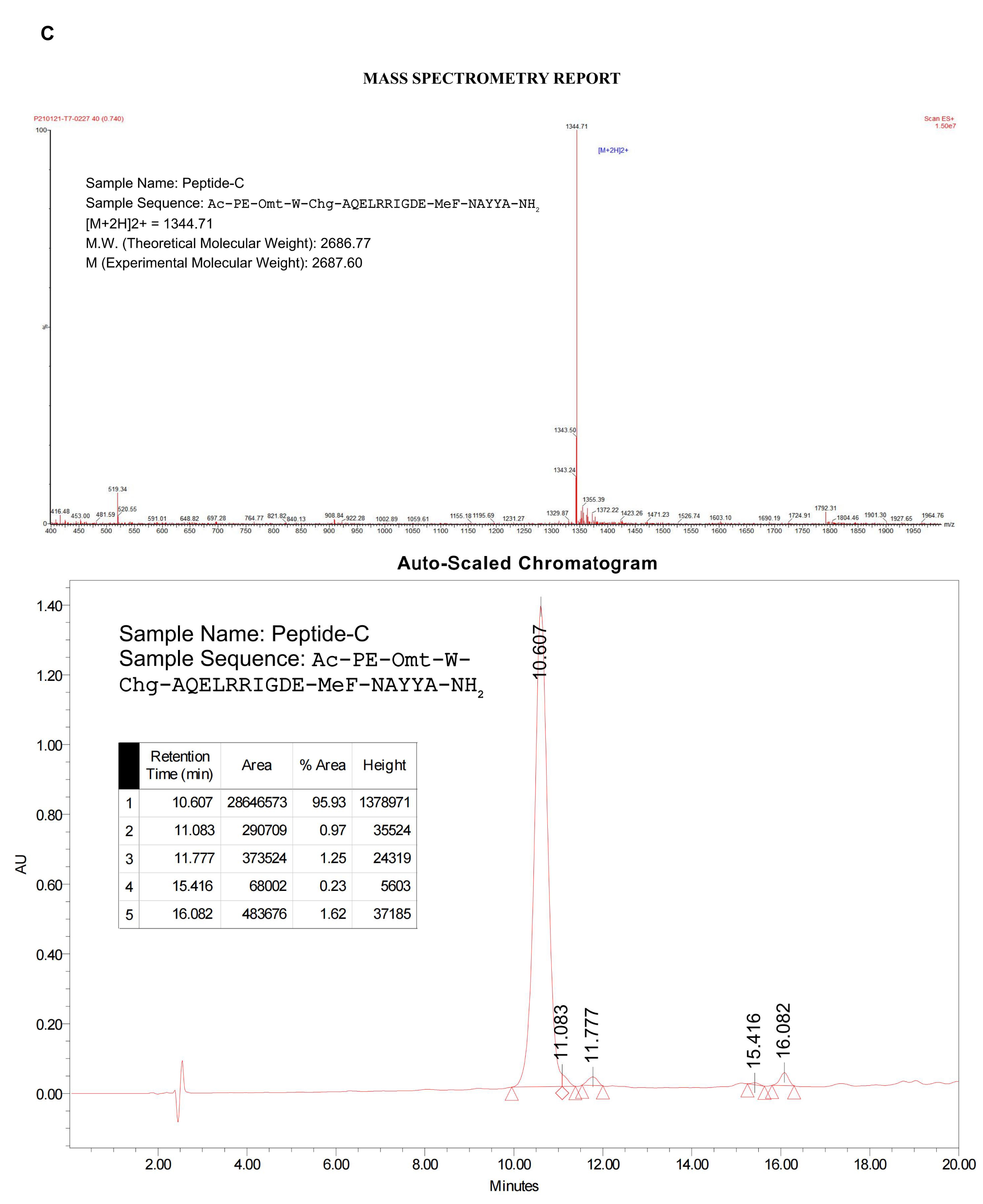


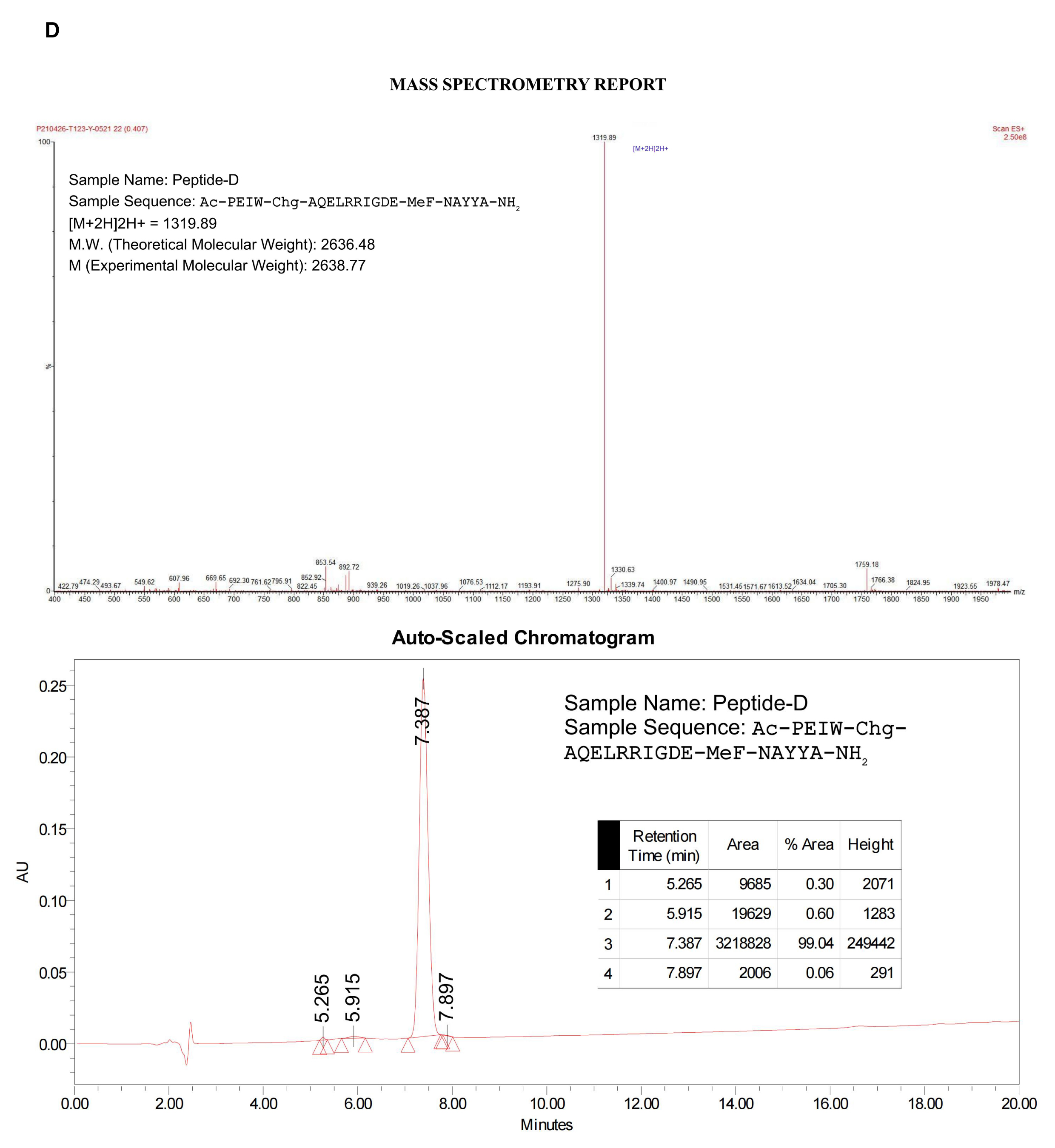


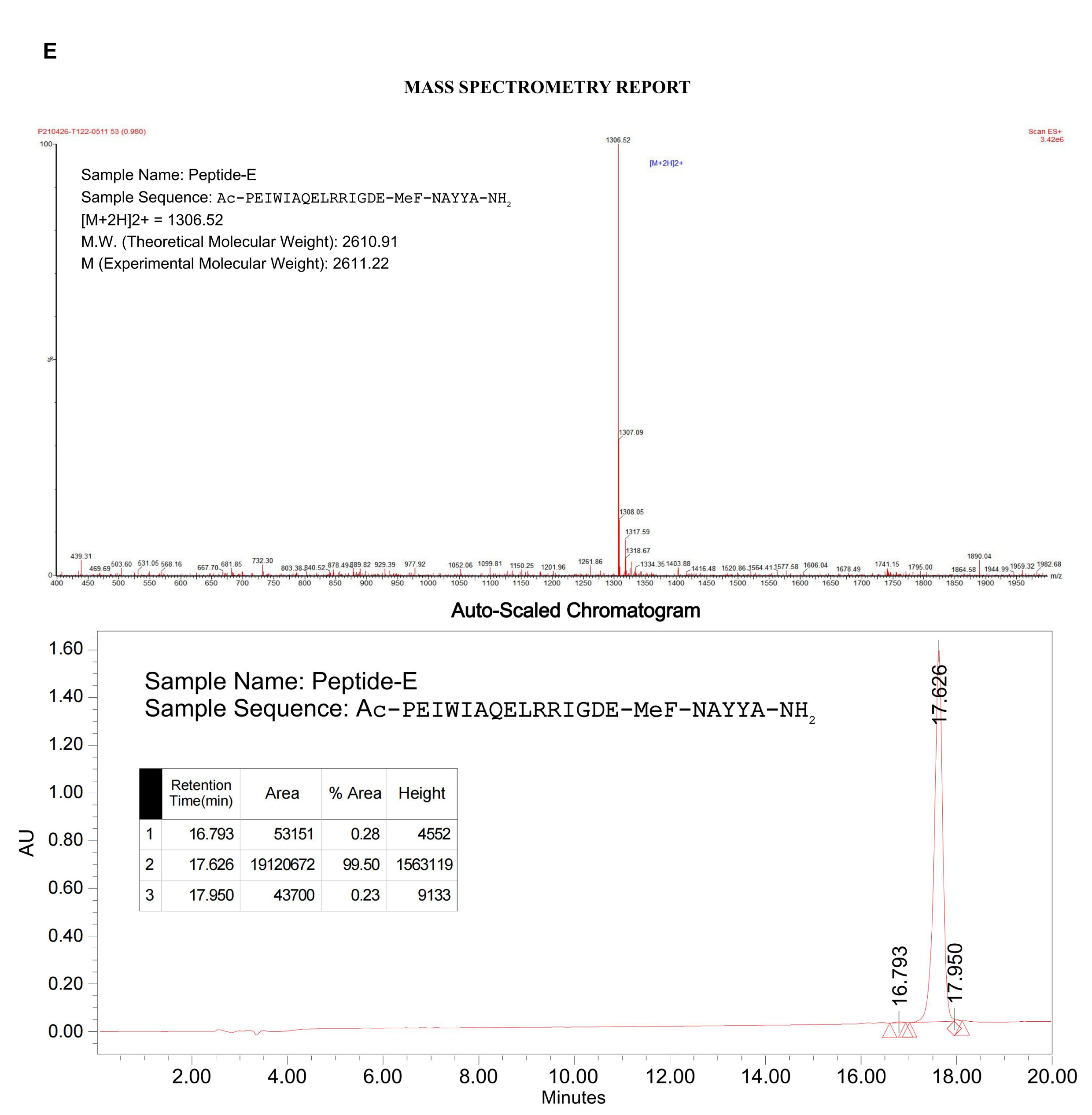


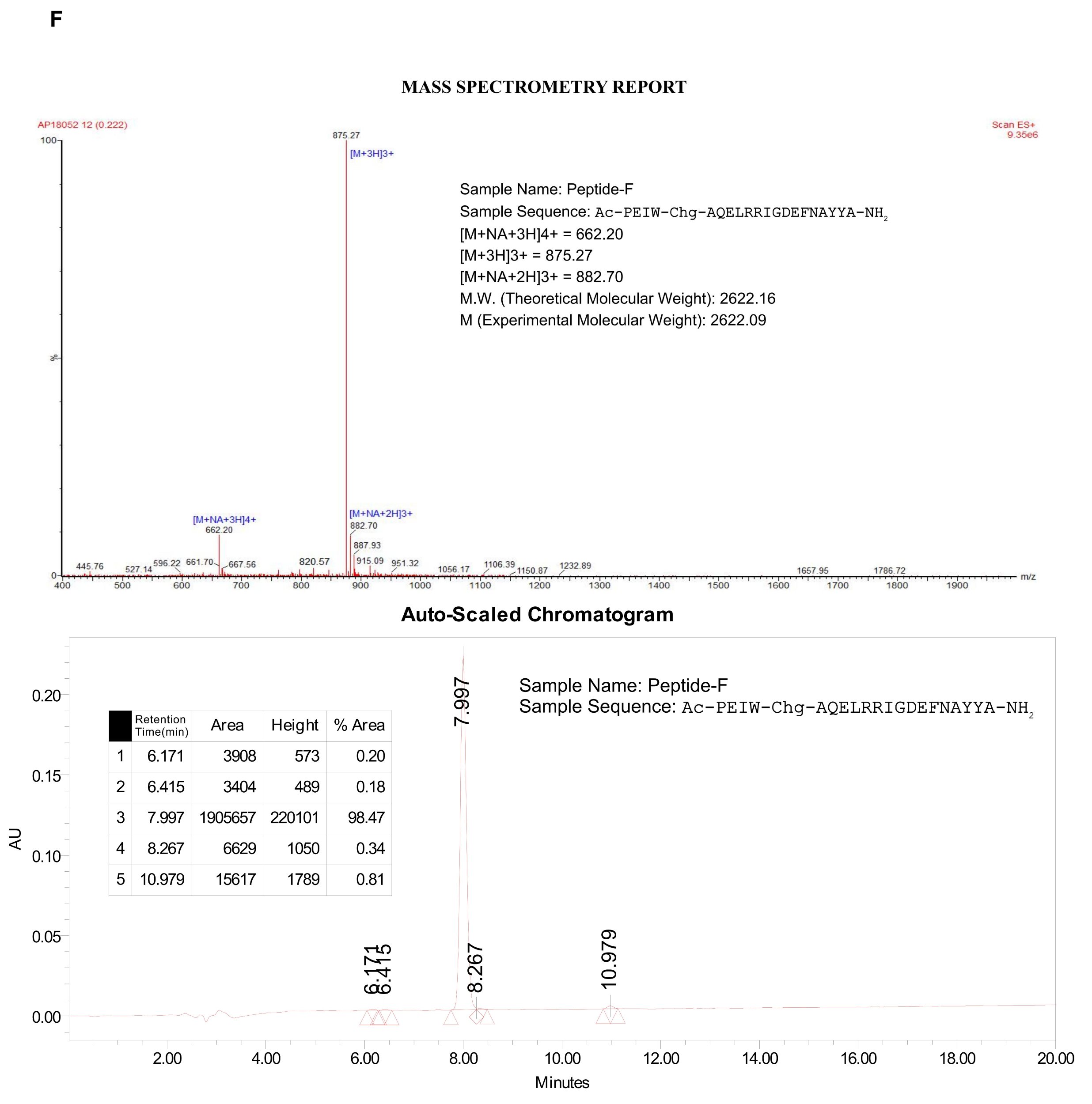


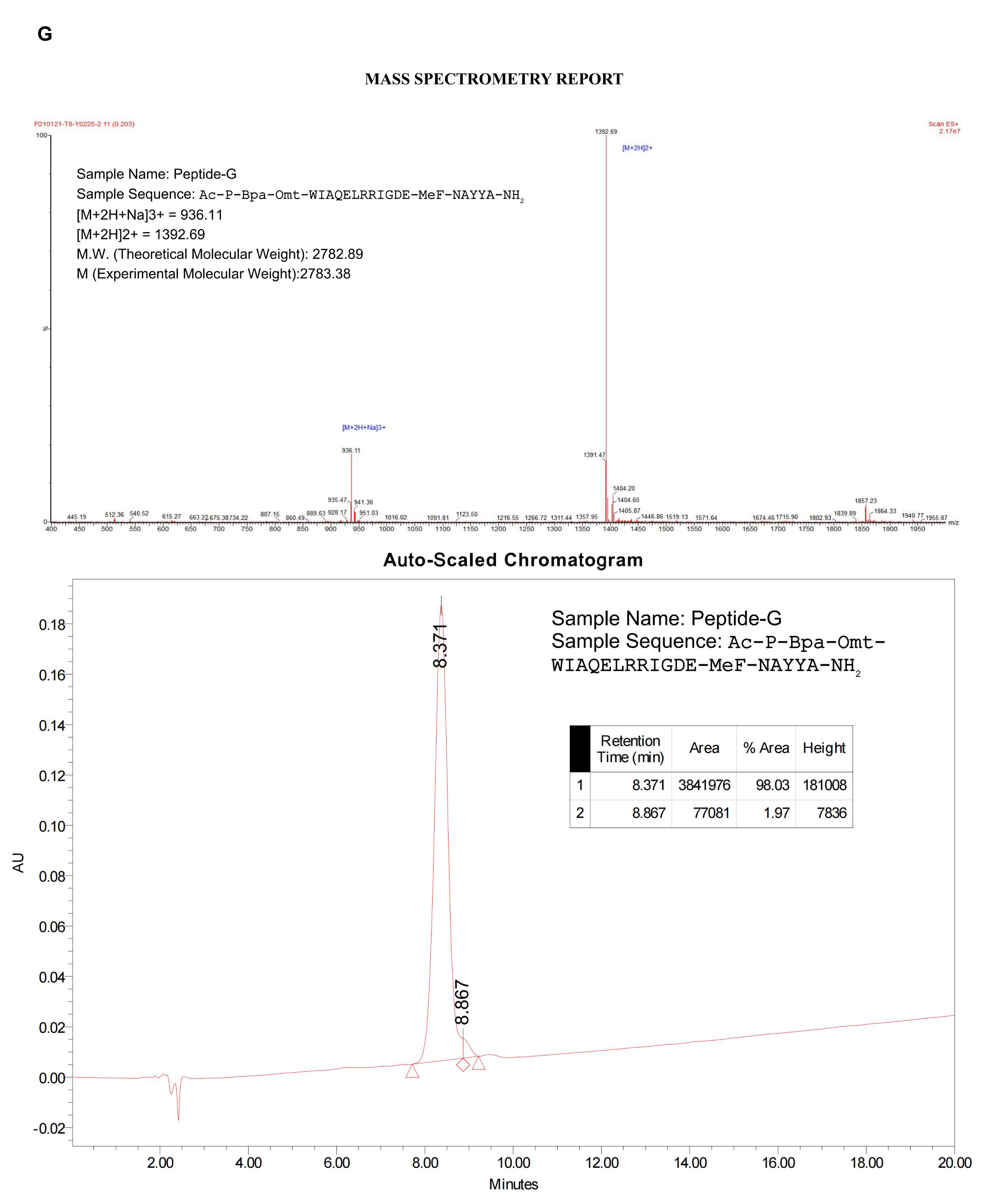


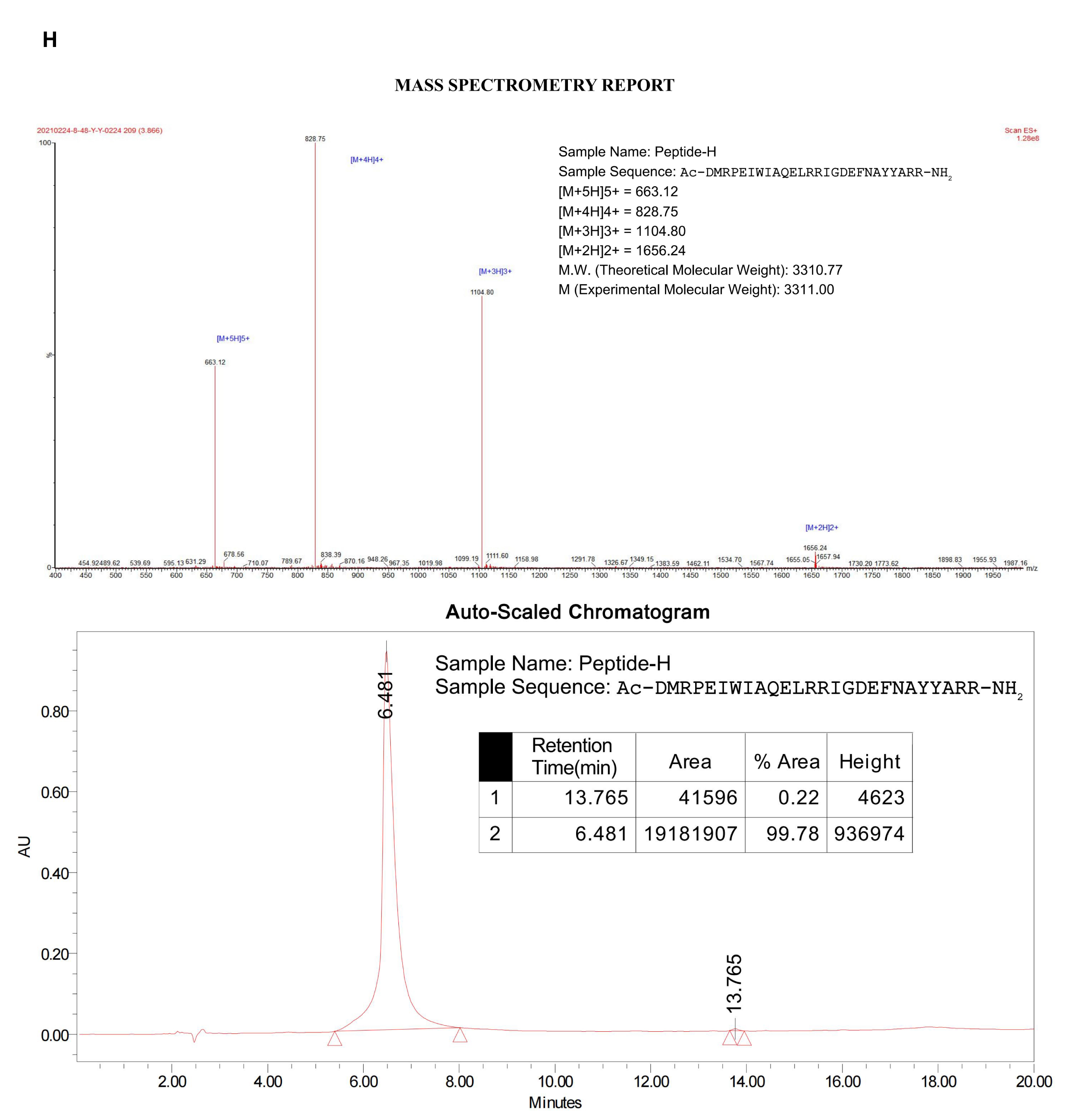


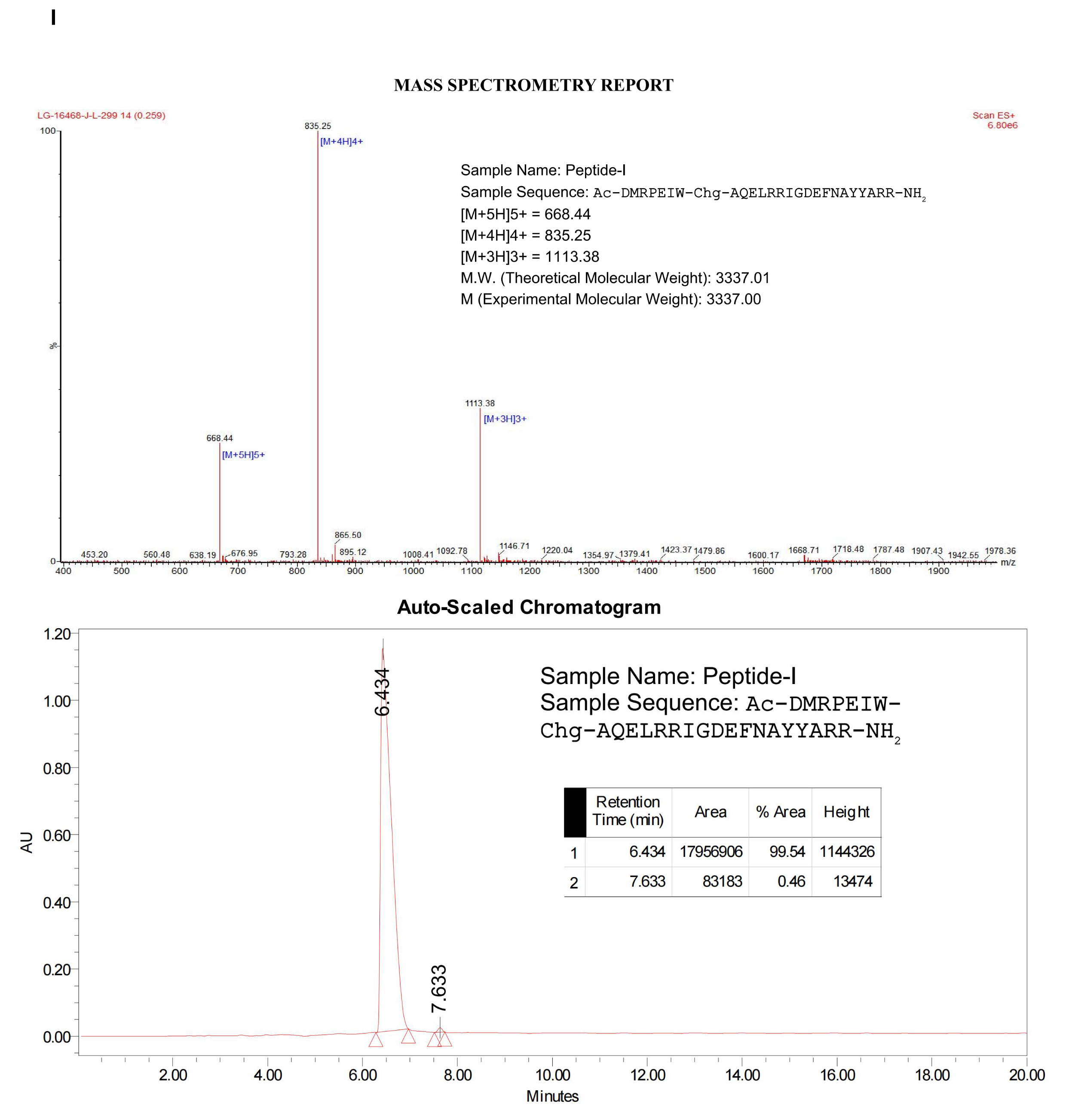


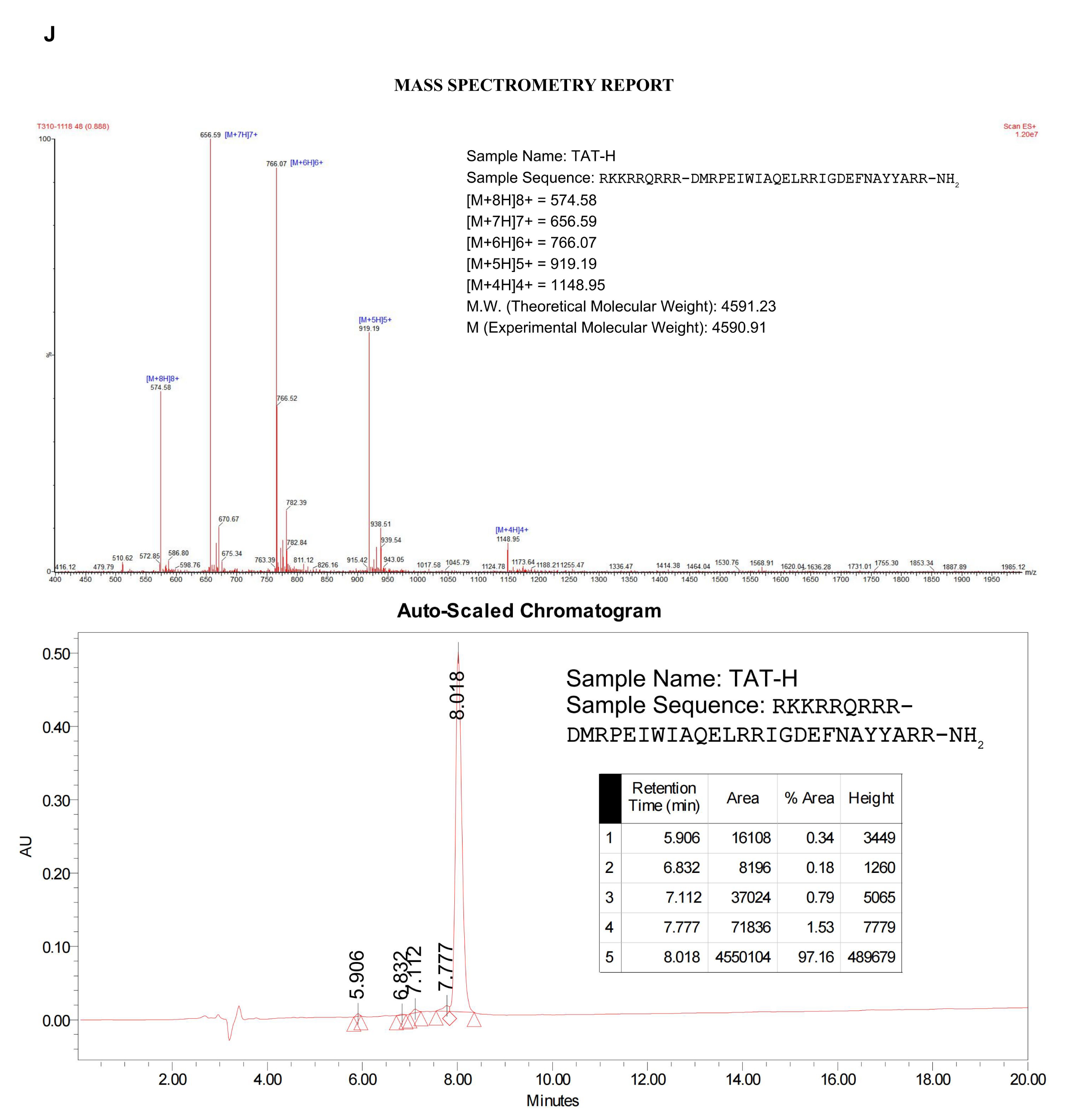


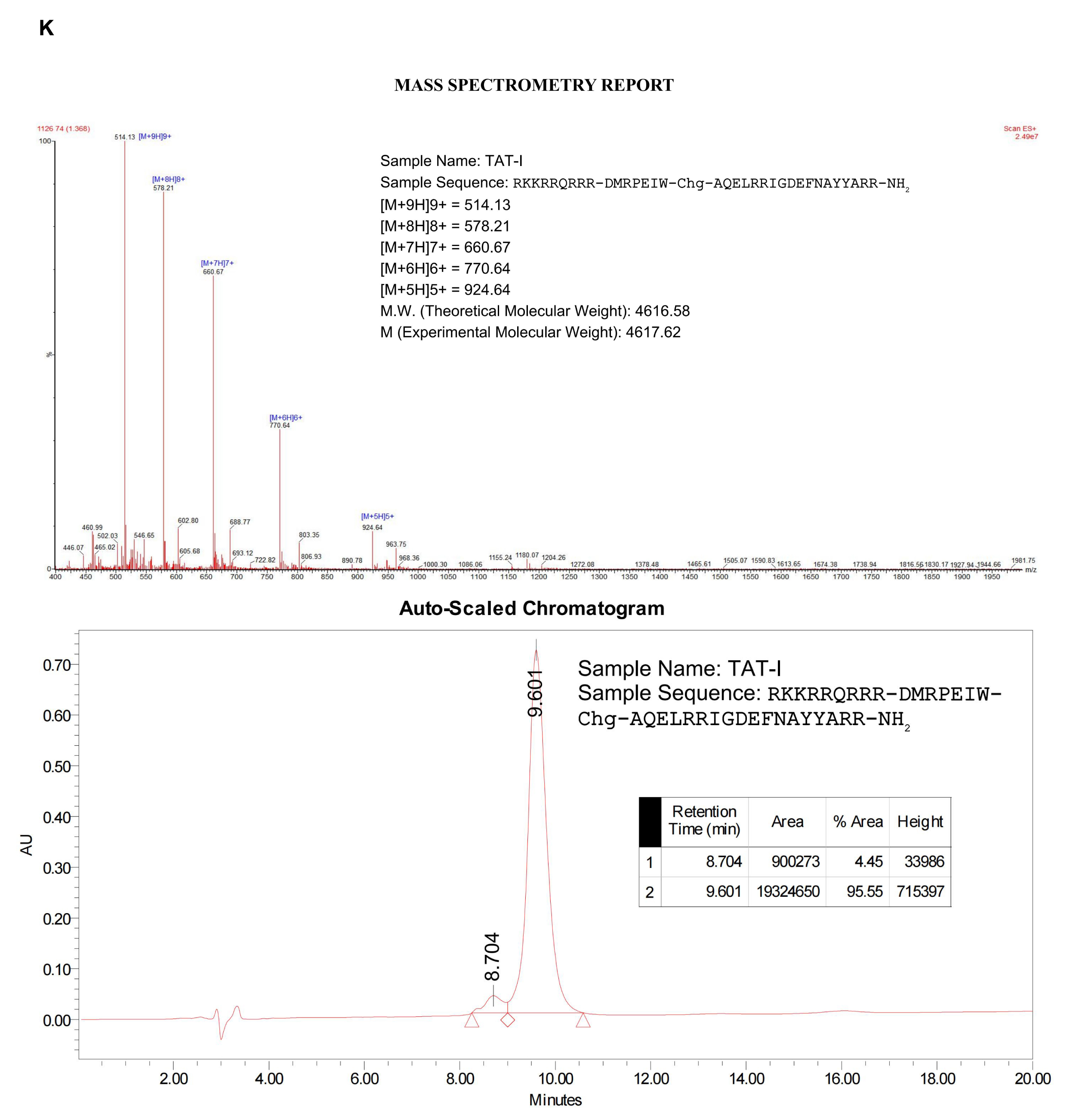


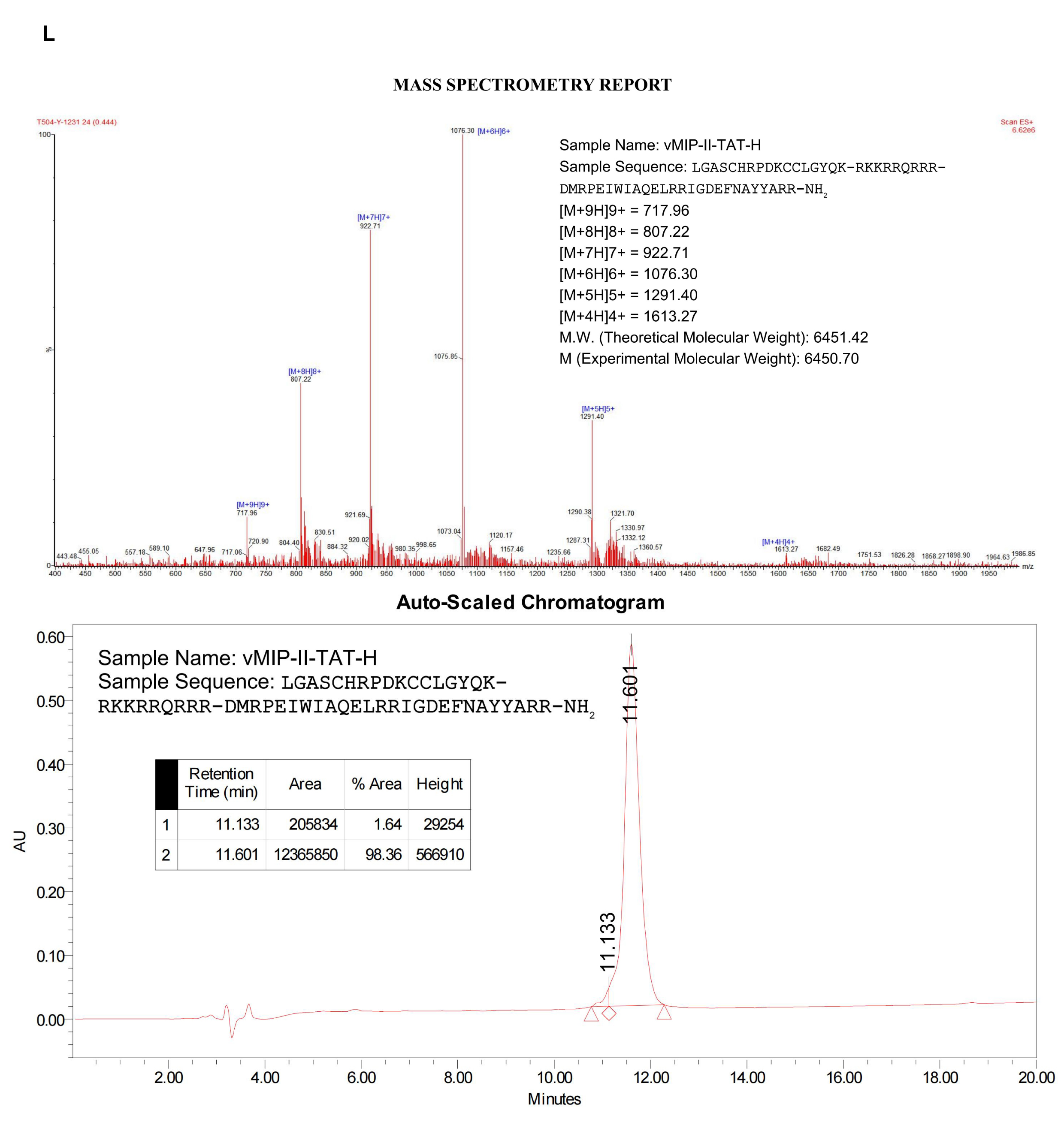


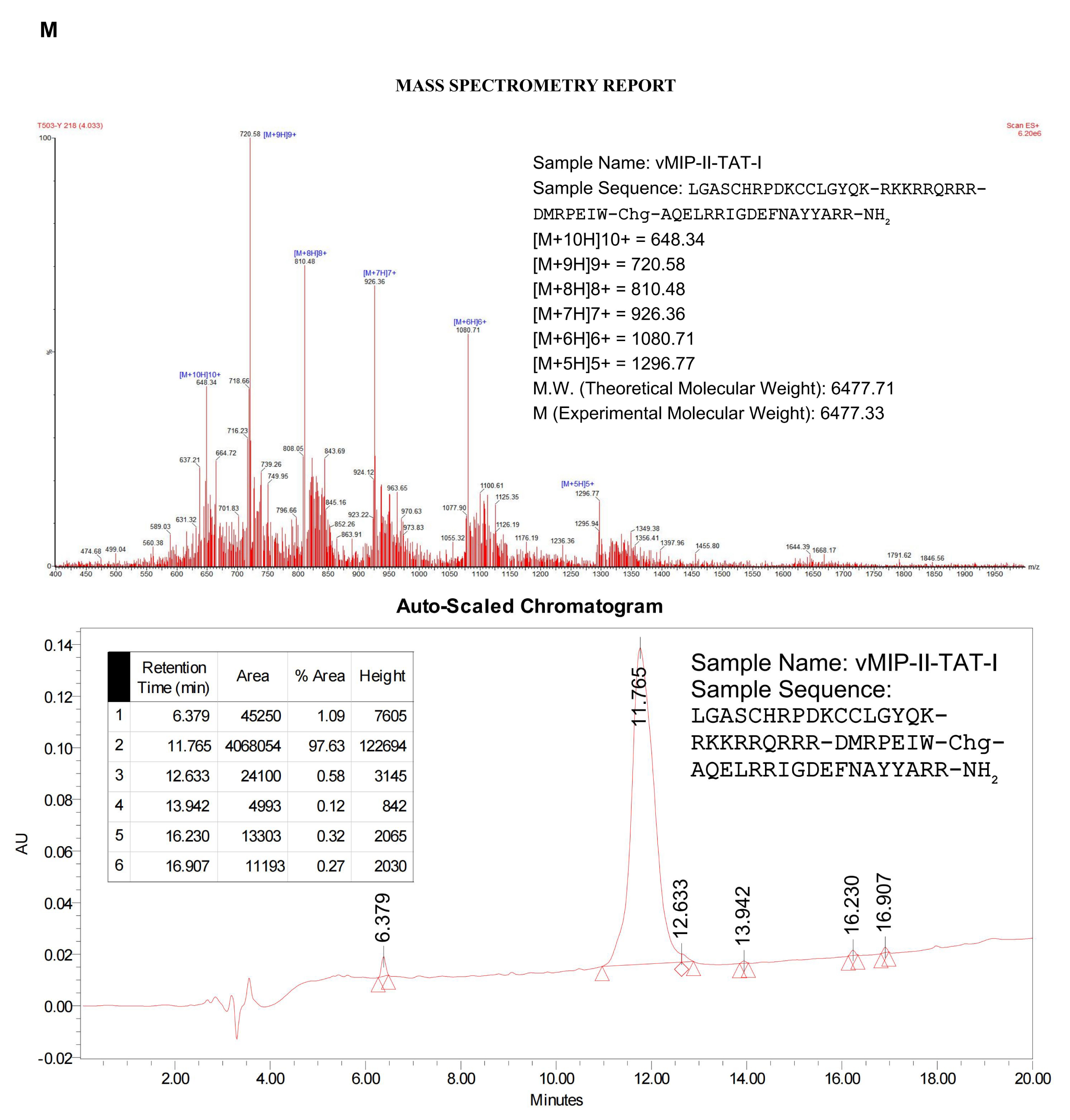


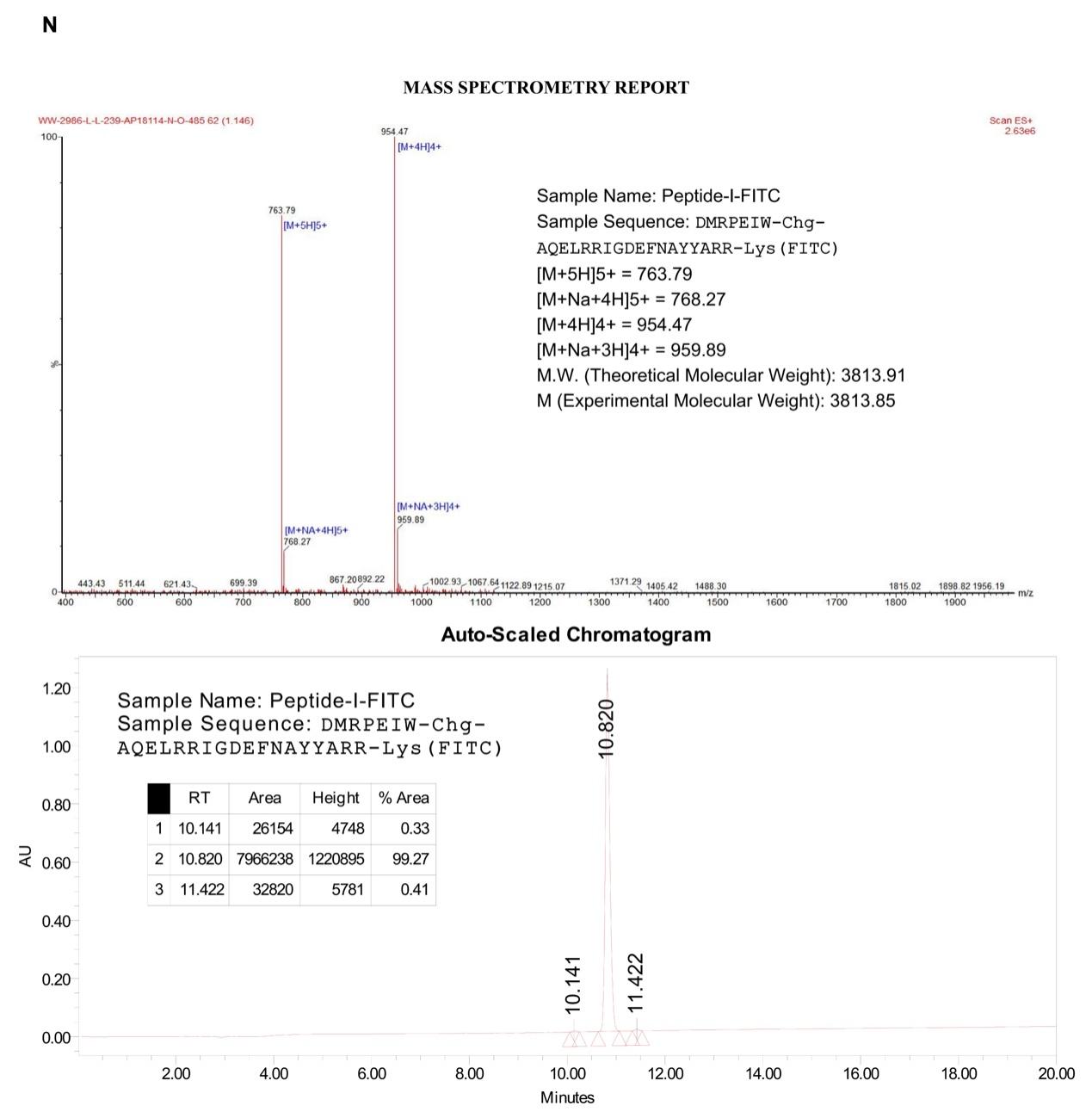

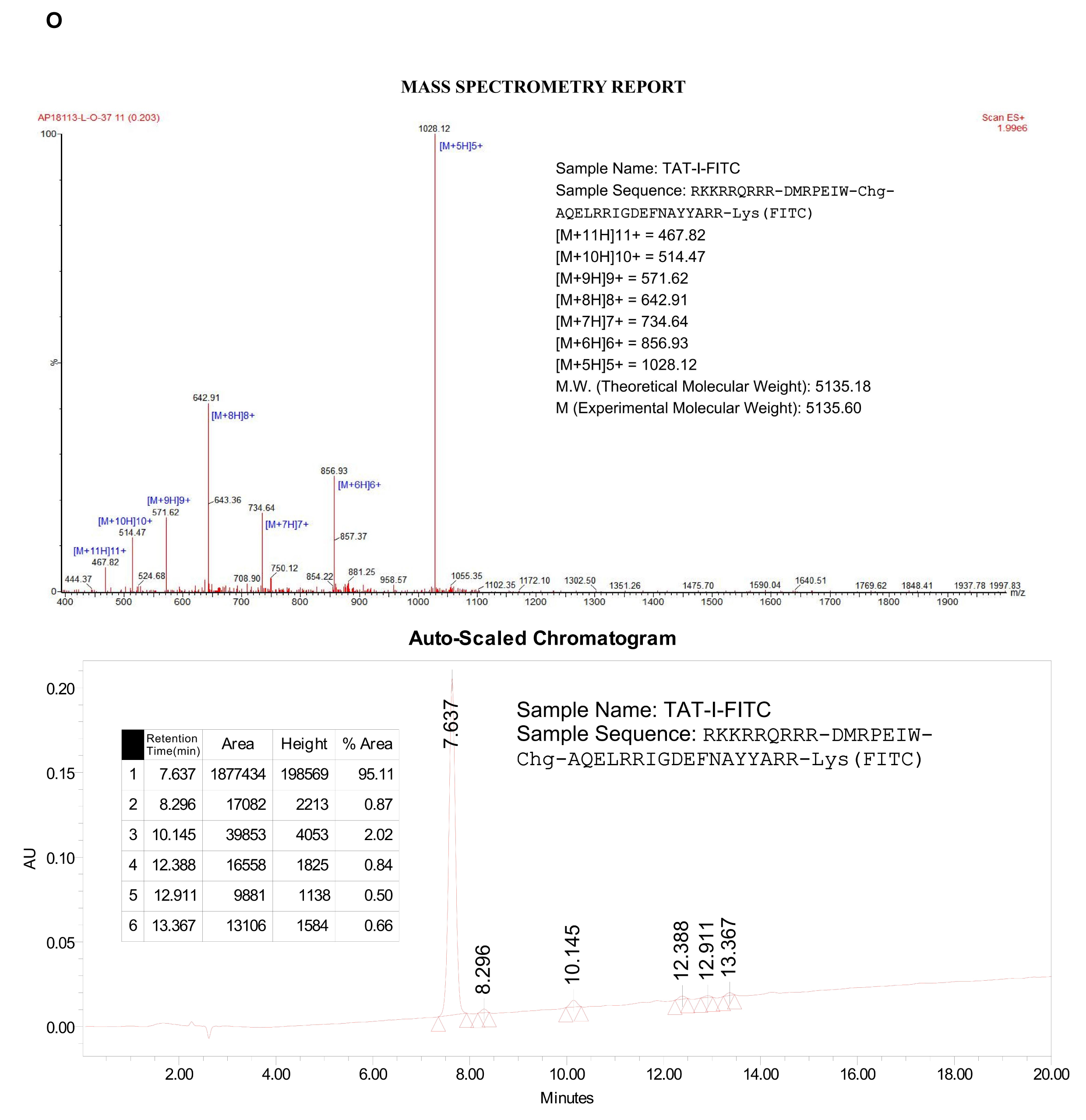

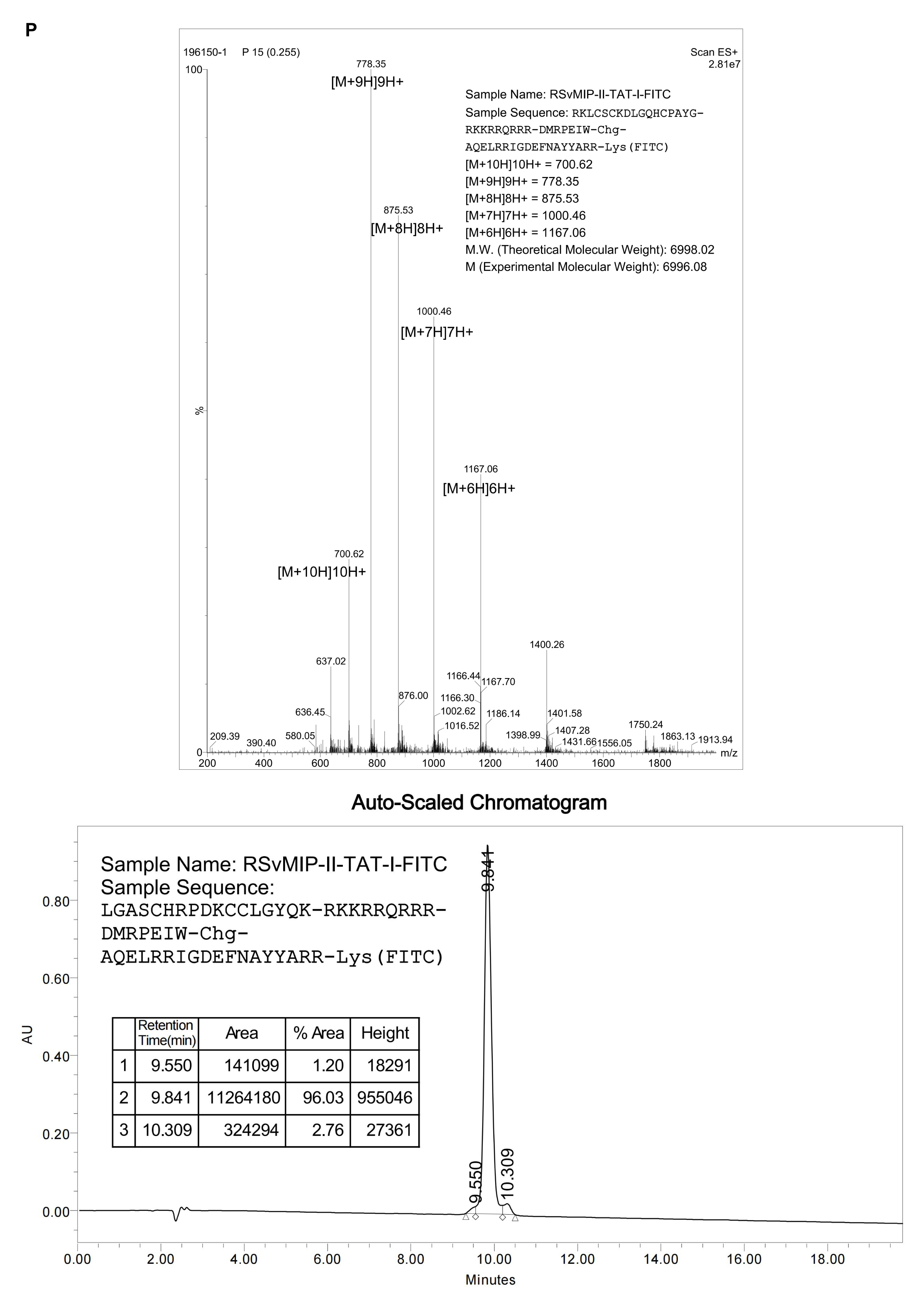

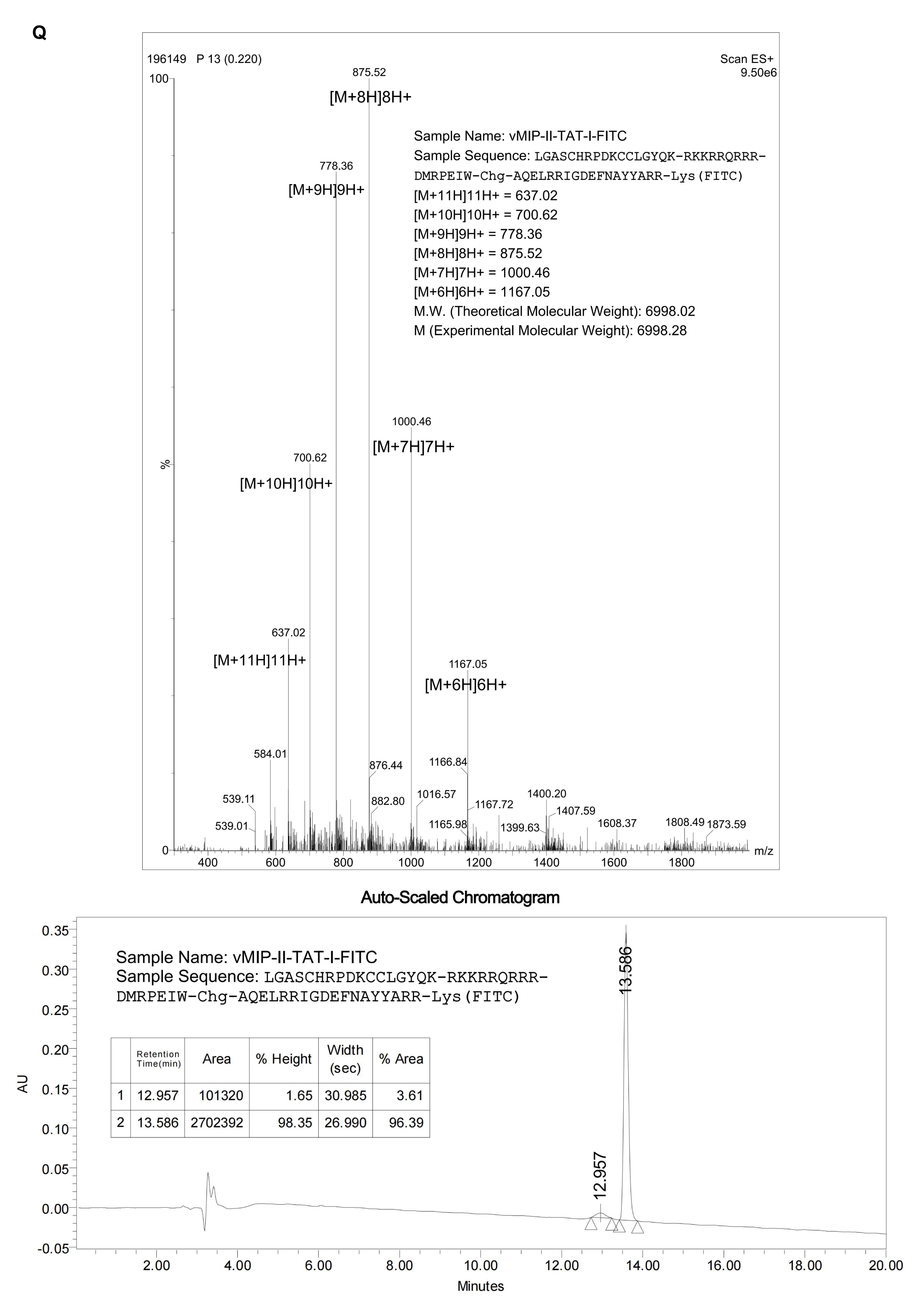


**Figure S2. Characterization of peptide sequences.** High-performance liquid chromatography and mass spectrometry detection peptide characterization data, TFA salt form, purity >95%.


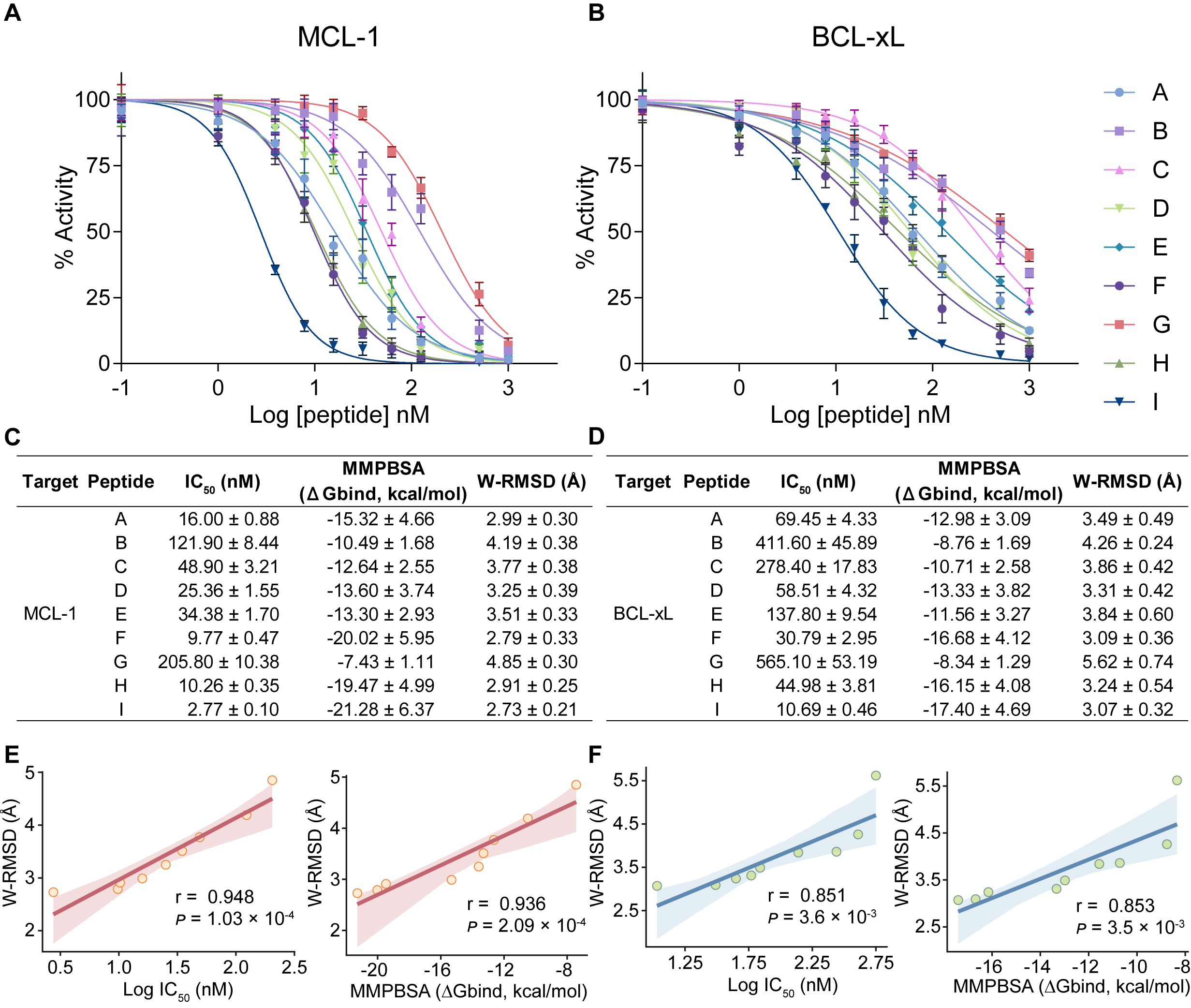


**Figure S3. Peptide binding affinity and correlation analysis.** (A-B) Cell-free TR-FRET detection of peptide binding affinity to MCL-1 and BCL-xL, respectively (n = 4). The activity of each peptide was assessed across a concentration range of 0.1 nM to 1000 nM, revealing varying degrees of affinity for both targets. Error bars represent mean ± SD. (C-D) IC_50_ values, MMPBSA-derived binding free energies (ΔGbind, kcal/mol), and W-RMSD values (n = 4) are provided for each peptide. The data are presented as mean ± SD. (E-F) Pearson correlation analysis between W-RMSD, IC_50,_ and ΔGbind for MCL-1 (E) and BCL-xL (F), each point represents the mean of four replicates, and shaded areas denote 95% confidence intervals. Correlation coefficients (r) and two-tailed *p* values were calculated using Pearson’s method, with p < 0.05 considered statistically significant.


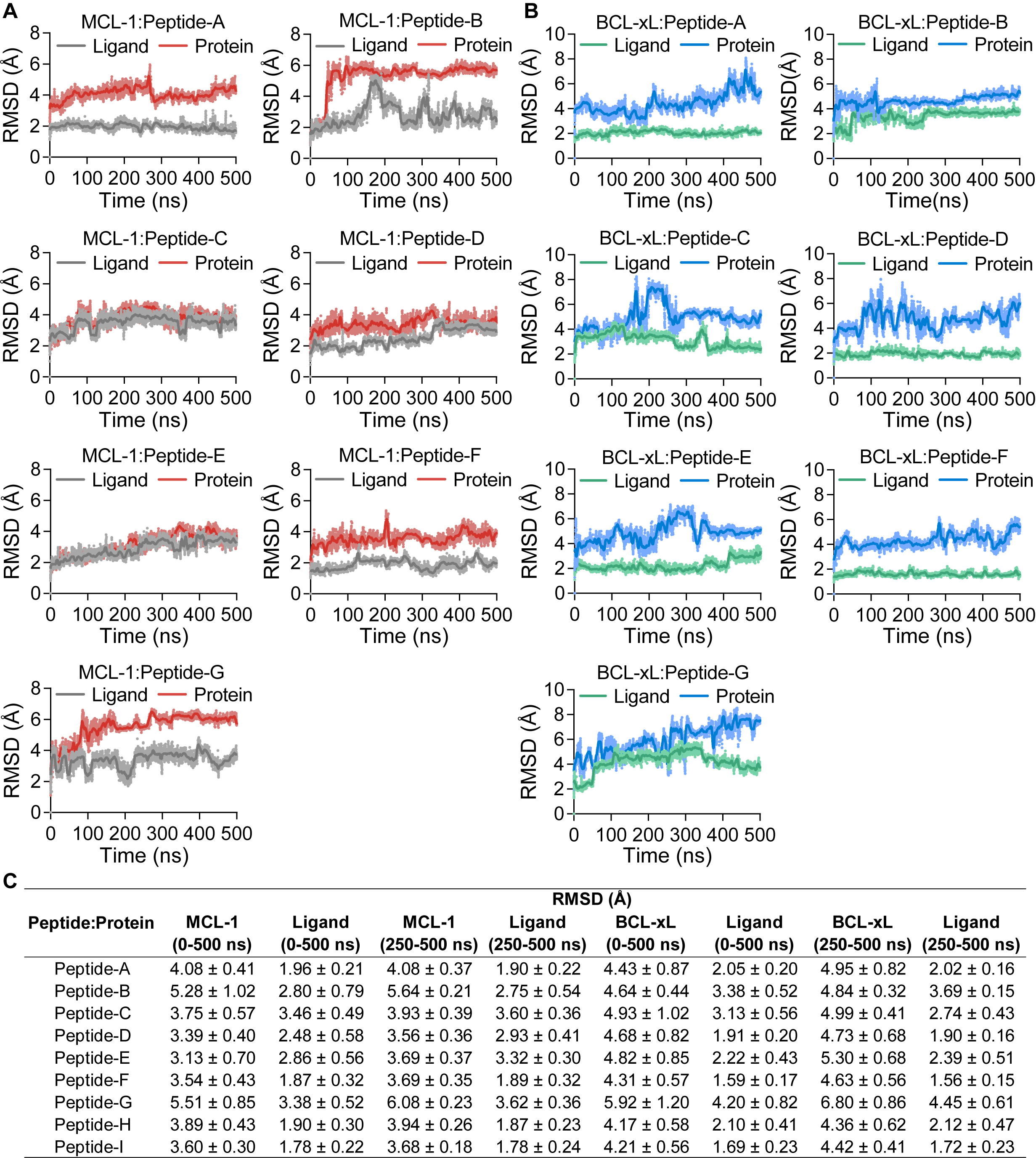


**Figure S4. RMSD analysis of peptides in complex with MCL-1 and BCL-xL.** (A-B) RMSD trajectories over the 500 ns MD simulation for MCL-1 and BCL-xL in complex with various peptides. (C) RMSD data (n = 4) for each peptide in complex with MCL-1 and BCL-xL, measured over two distinct time intervals: 0-500 ns and 250-500 ns. Data are presented as mean ± SD.


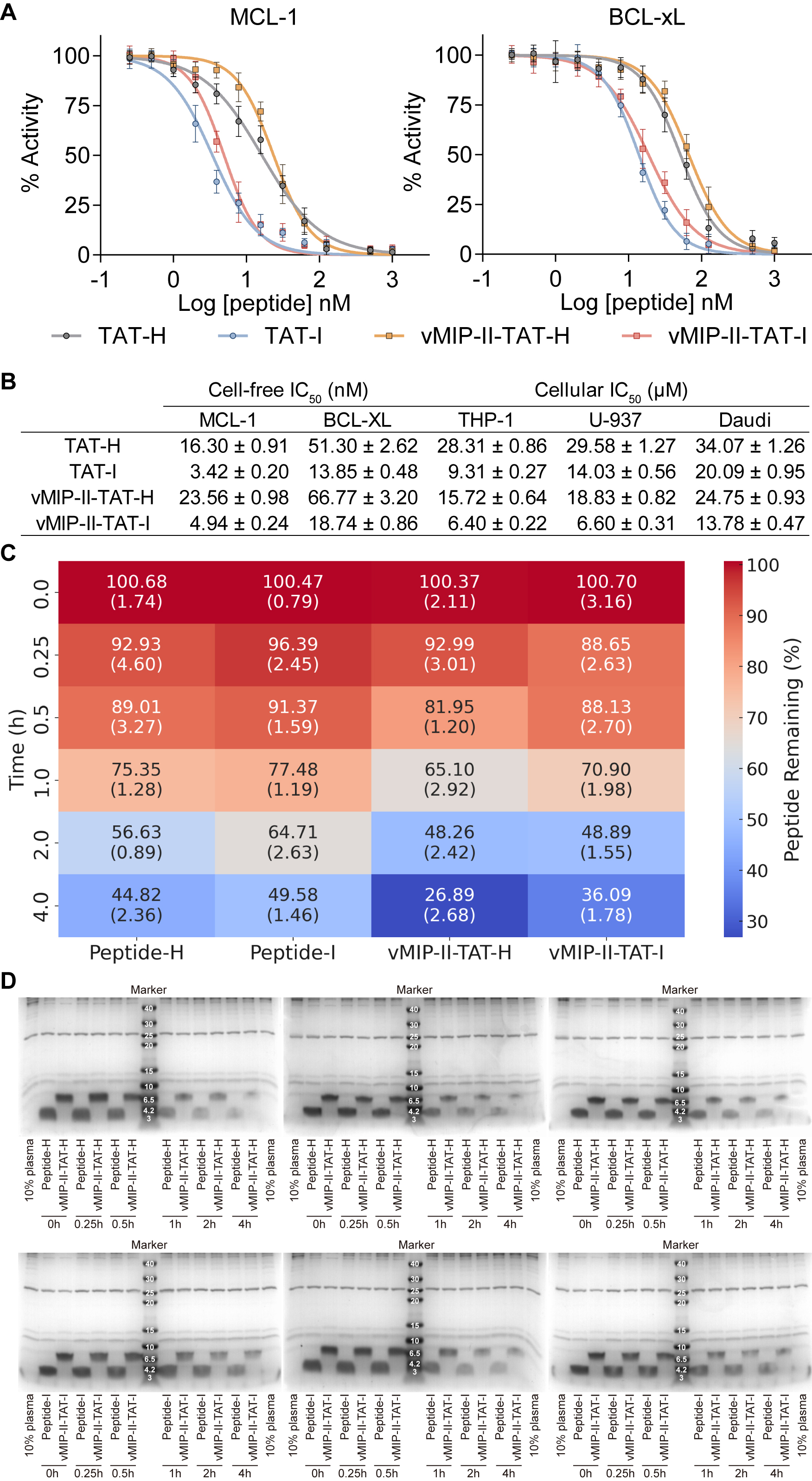


**Figure S5. Binding affinity and plasma degradation analysis of TAT and vMIP-II modified peptides.** (A) Cell-free TR-FRET detection of TAT and vMIP-II modified peptides binding affinity to MCL-1 and BCL-xL. The binding activity was measured across various concentrations ranging from 0.25 nM to 1000 nM (n = 4). Error bars represent mean ± SD. (B) The detailed data of IC_50_ values for each peptide in both cell-free and cellular assays (n = 4) are presented as mean ± SD. (C) Heatmap of Peptide-H, Peptide-I, vMIP-II-TAT-H, and vMIP-II-TAT-I stability analysis in plasma (n = 3), where the upper part of each square represents the mean percentage of peptide remaining, and the lower part shows the corresponding SD. (D) SDS-PAGE analysis showing the stability of the four peptides over time in plasma (n = 3), indicating the degradation rates of each peptide at different time intervals (0 h, 0.25 h, 0.5 h, 1 h, and 4 h).


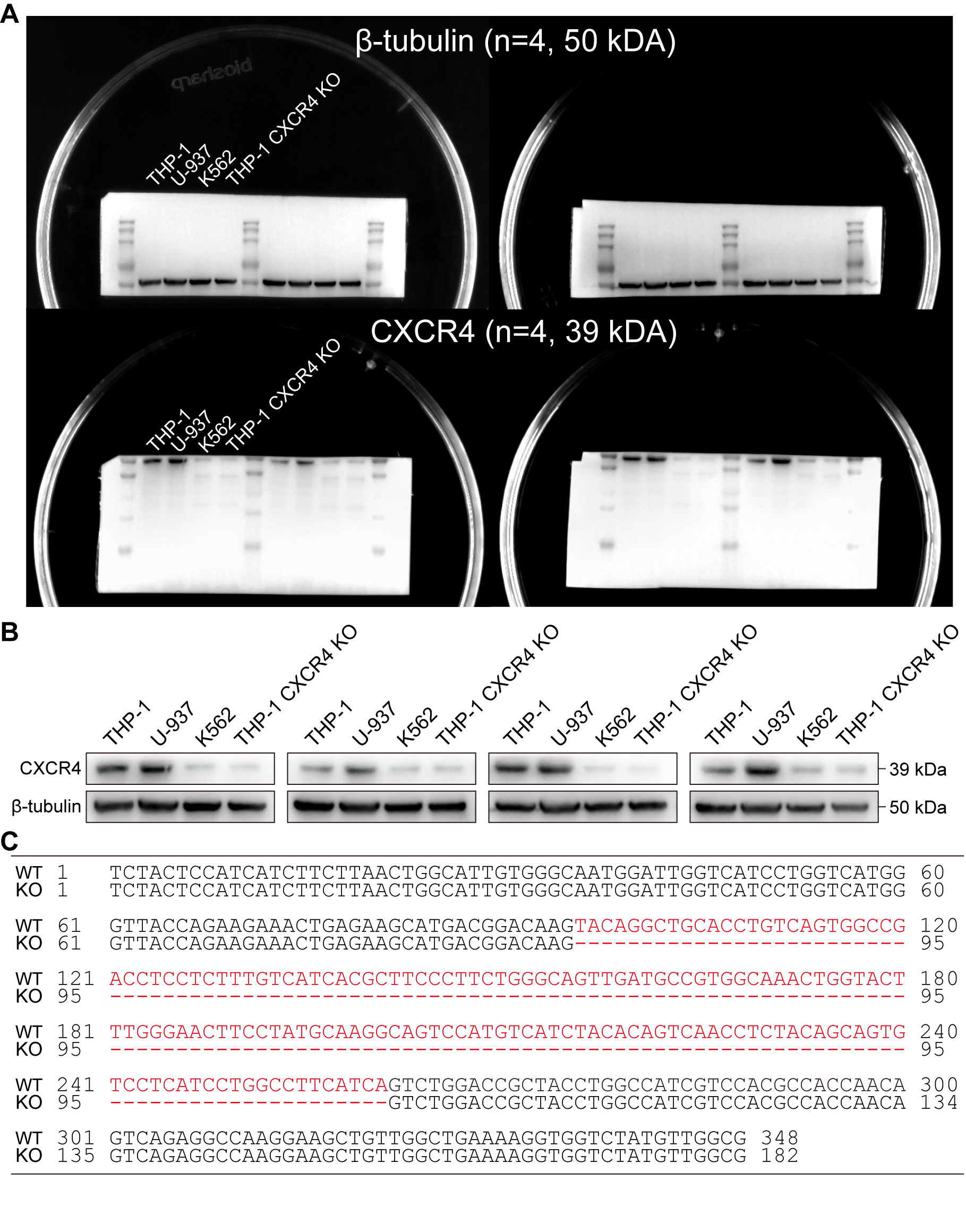


**Figure S6. Western Blot Analysis of CXCR4 Expression and Knockout Verification.** (A) The original protein imprint showing Western blot results (n = 4) for β-tubulin (50 kDa) and CXCR4 (39 kDa) in THP-1, U-937, K562, and THP-1-CXCR4 KO cell lines. (B) Quantitative analysis of CXCR4 expression (n = 4). High CXCR4 expression in THP-1 and U-937 cells, low expression in K562 cells, and successful CXCR4 knockout in the THP-1-CXCR4 KO cell line. (C) The sequence comparison between the wild type (WT) and knockout (KO) CXCR4 genes in THP-1 cells. The KO sequence contains a 166 bp deletion, marked in red with dashed lines, introduced by CRISPR-Cas9 gene editing, disrupting the normal gene sequence.


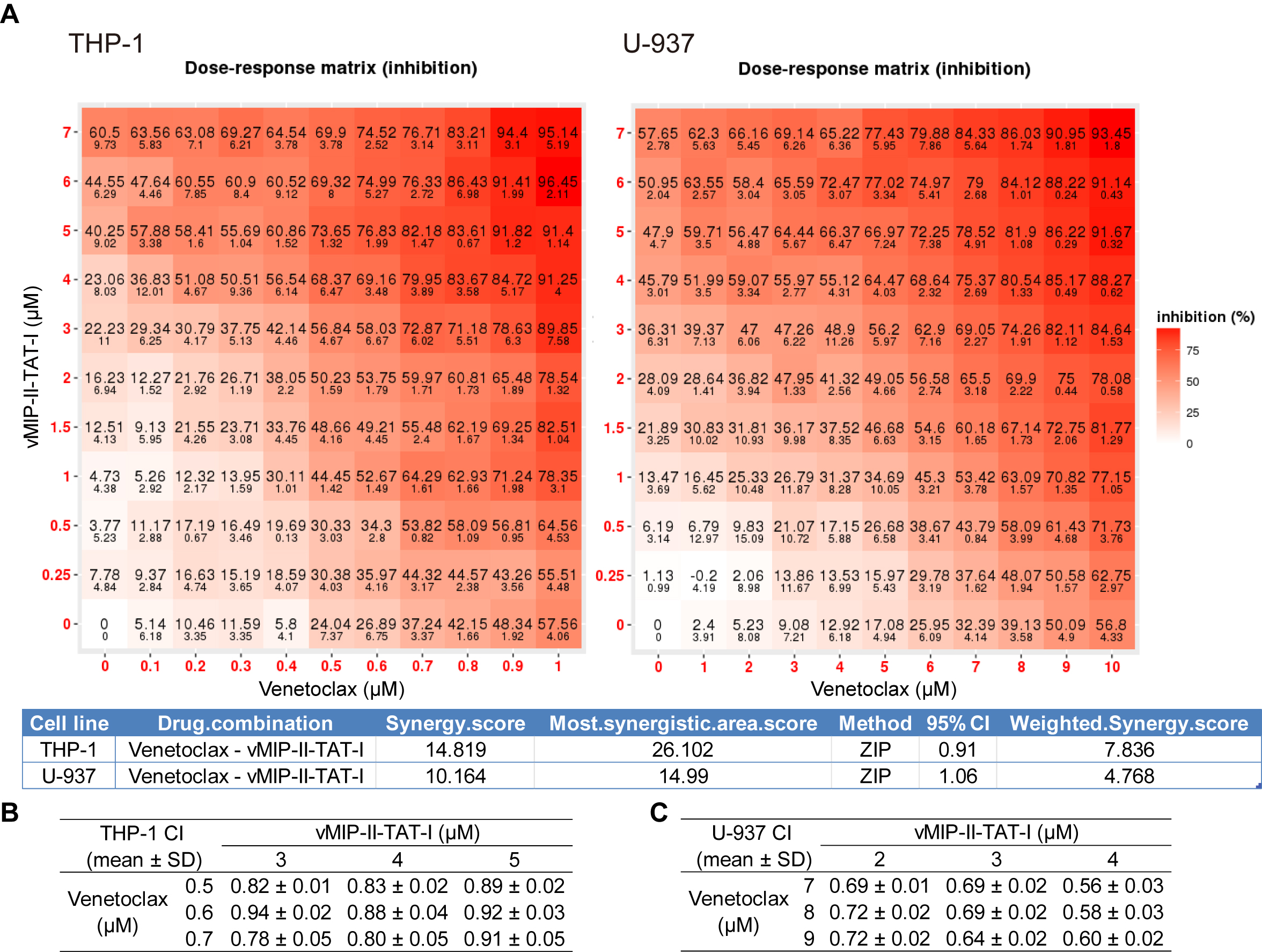


**Figure S7. Synergy analysis of vMIP-II-TAT-I and Venetoclax.** (A) Inhibition rate heat maps for THP-1 and U-937 cells show the effects of varying concentrations of vMIP-II-TAT-I and Venetoclax in combination (n = 4). The upper part of the boxes displays mean inhibition percentages and the lower part shows the SD for each drug combination. (B-C) Combination index (CI) values for the optimal combination concentrations of vMIP-II-TAT-I and Venetoclax in THP-1 and U-937 cells, with mean ± SD for each concentration (n = 4).


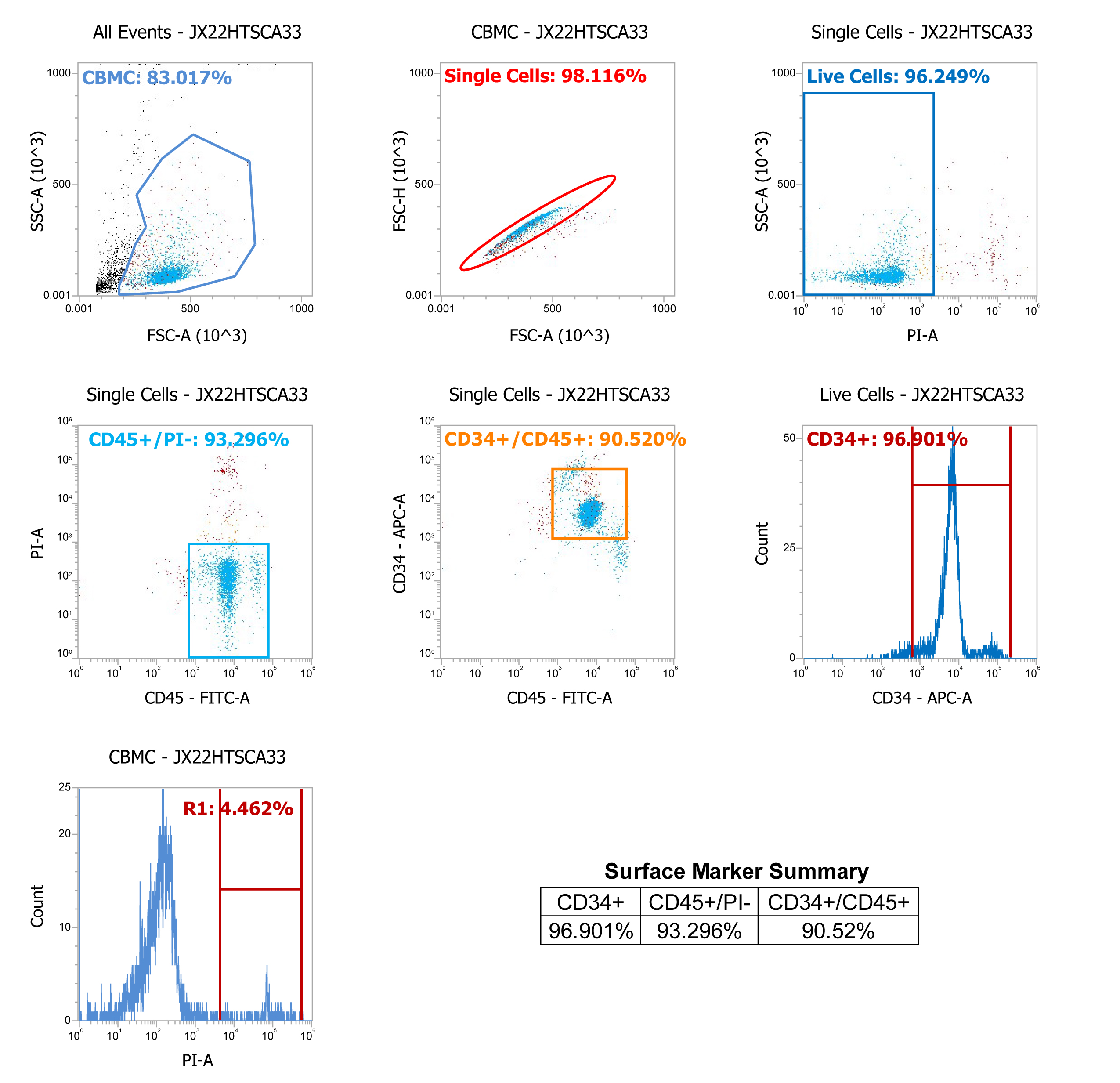


**Figure S8. Analysis of human cord blood purified CD34+ progenitor cells.** Flow cytometry analysis reveals that 96.901% of cells are CD34+, with 93.296% CD45+/PI- and 90.520% CD34+/CD45+, confirming the high purity and viability of the human hematopoietic stem cells (HSCs) population and demonstrating the successful isolation of highly pure CD34+ HSCs from umbilical cord blood.


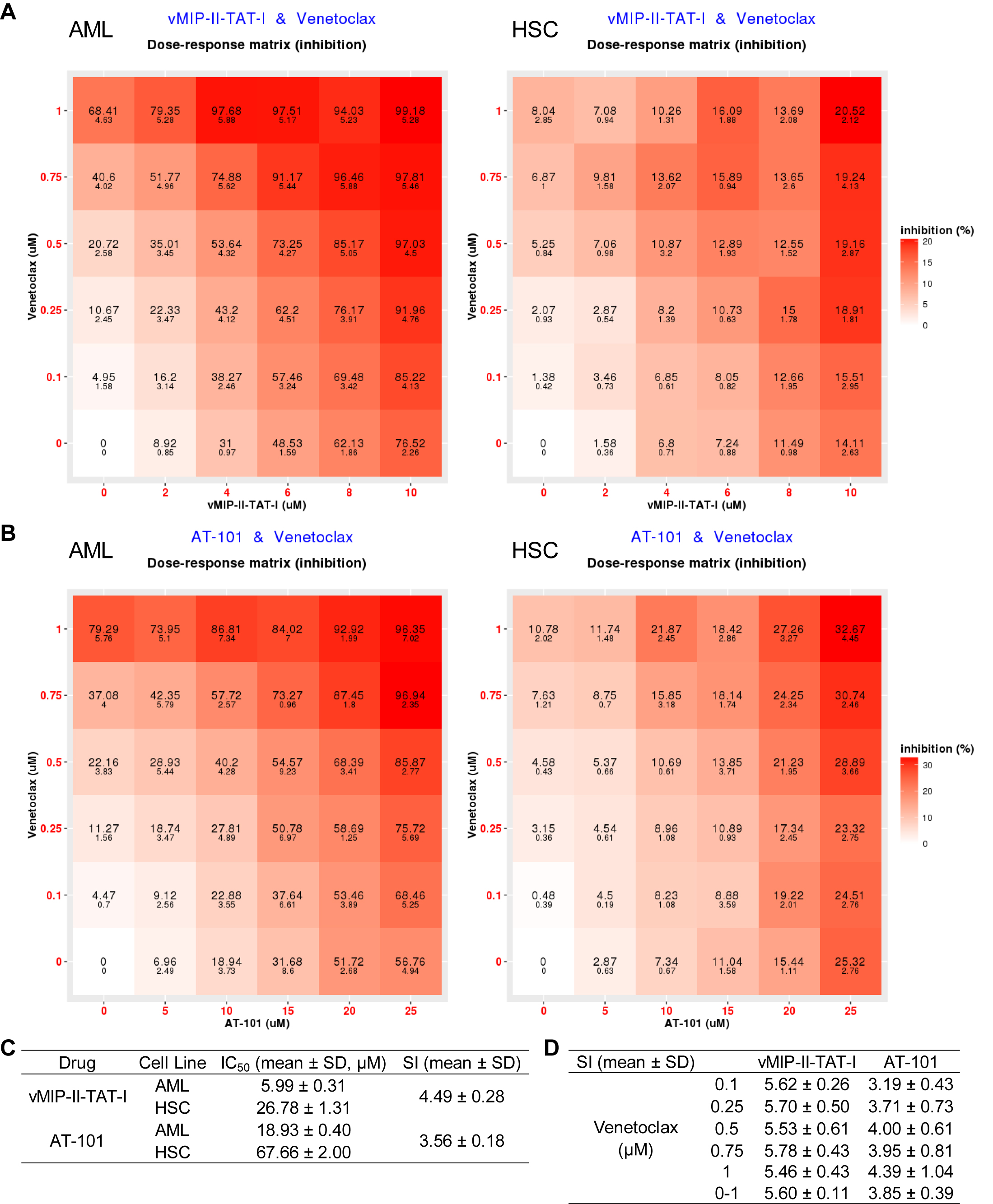


**Figure S9. Synergy and selectivity of vMIP-II-TAT-I with Venetoclax.** (A-B) Heat map showing the inhibition rate of AML (THP-1) and HSC cells after treatment with the combination of vMIP-II-TAT-I (A) or AT-101 (B) and Venetoclax (n= 4). The number above each box represents the mean inhibition percentage, while the number below indicates the SD. (C) CCK-8 assay results for vMIP-II-TAT-I and AT-101 in AML and HSC models, displaying the detailed cytotoxicity data with IC_50_ values (n = 5). (D) Detailed data on the selectivity index (SI) for the combination of vMIP-II-TAT-I or AT-101 with Venetoclax, showing the drug concentrations of vMIP-II-TAT-I or AT-101, in combination with varying concentrations of Venetoclax (n = 5). Data are presented as mean ± SD.


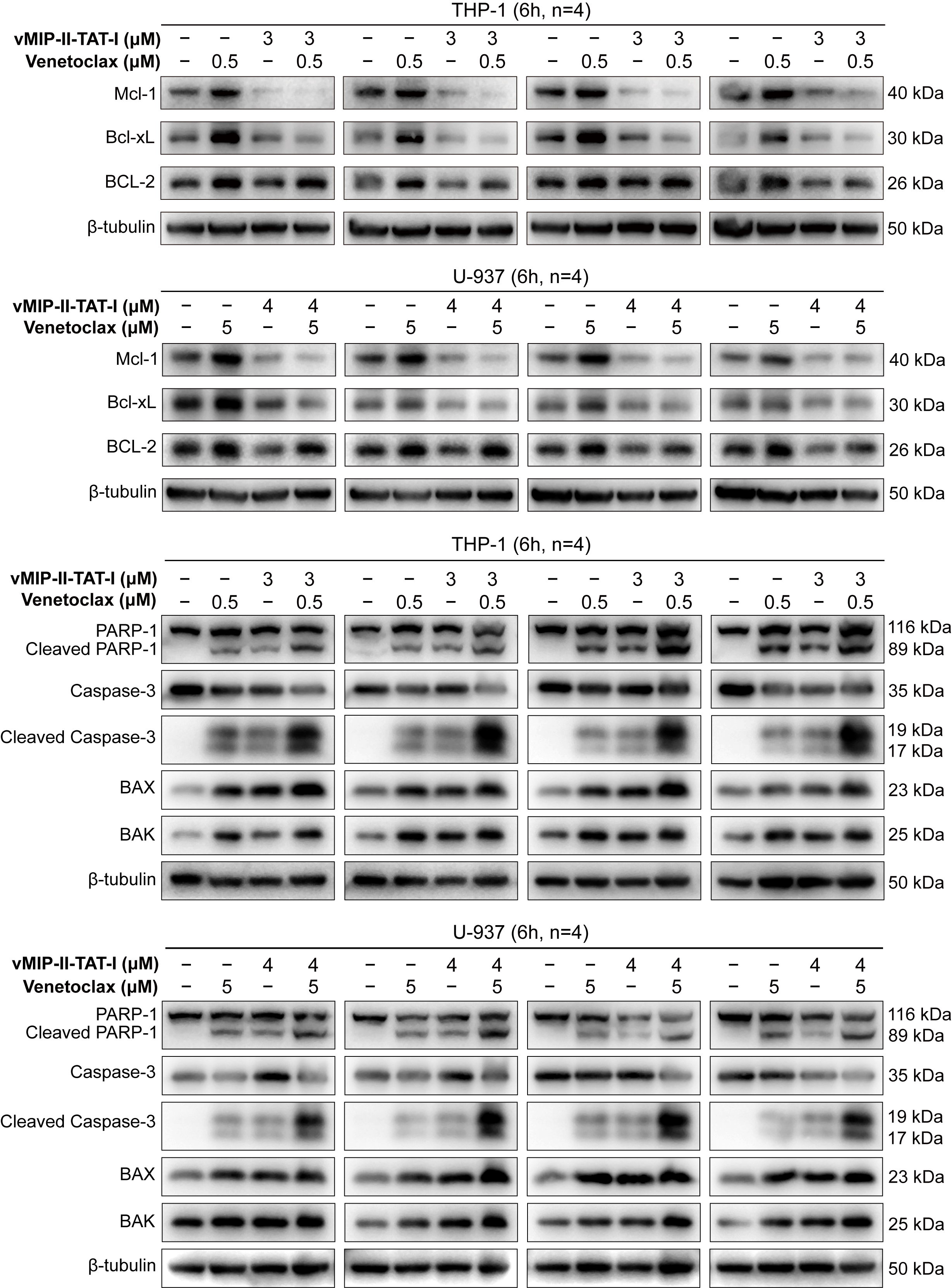


**Figure S10. Reproducibility of western blot analysis.** Western blot analysis of protein expression in THP-1 and U-937 cells after treatment with vMIP-II-TAT-I and Venetoclax. The top panels show the expression of anti-apoptotic proteins in both cell lines after 6-hour treatment with varying concentrations of vMIP-II-TAT-I and Venetoclax. The lower panels show the expression of apoptosis-related proteins.β-tubulin was used as a loading control. Data are shown for THP-1 (6 h, n = 4) and U-937 (6 h, n = 4) cells.


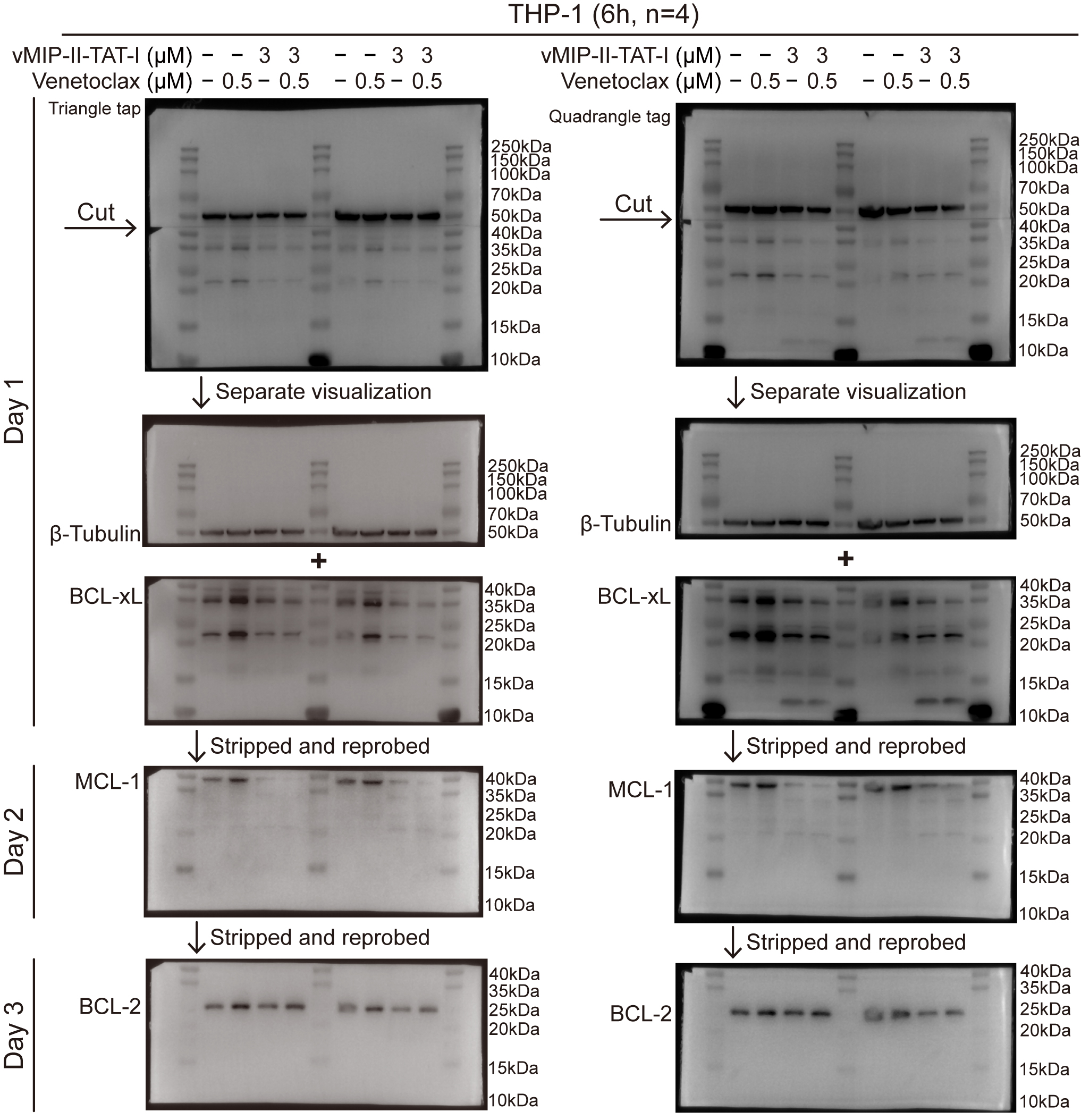

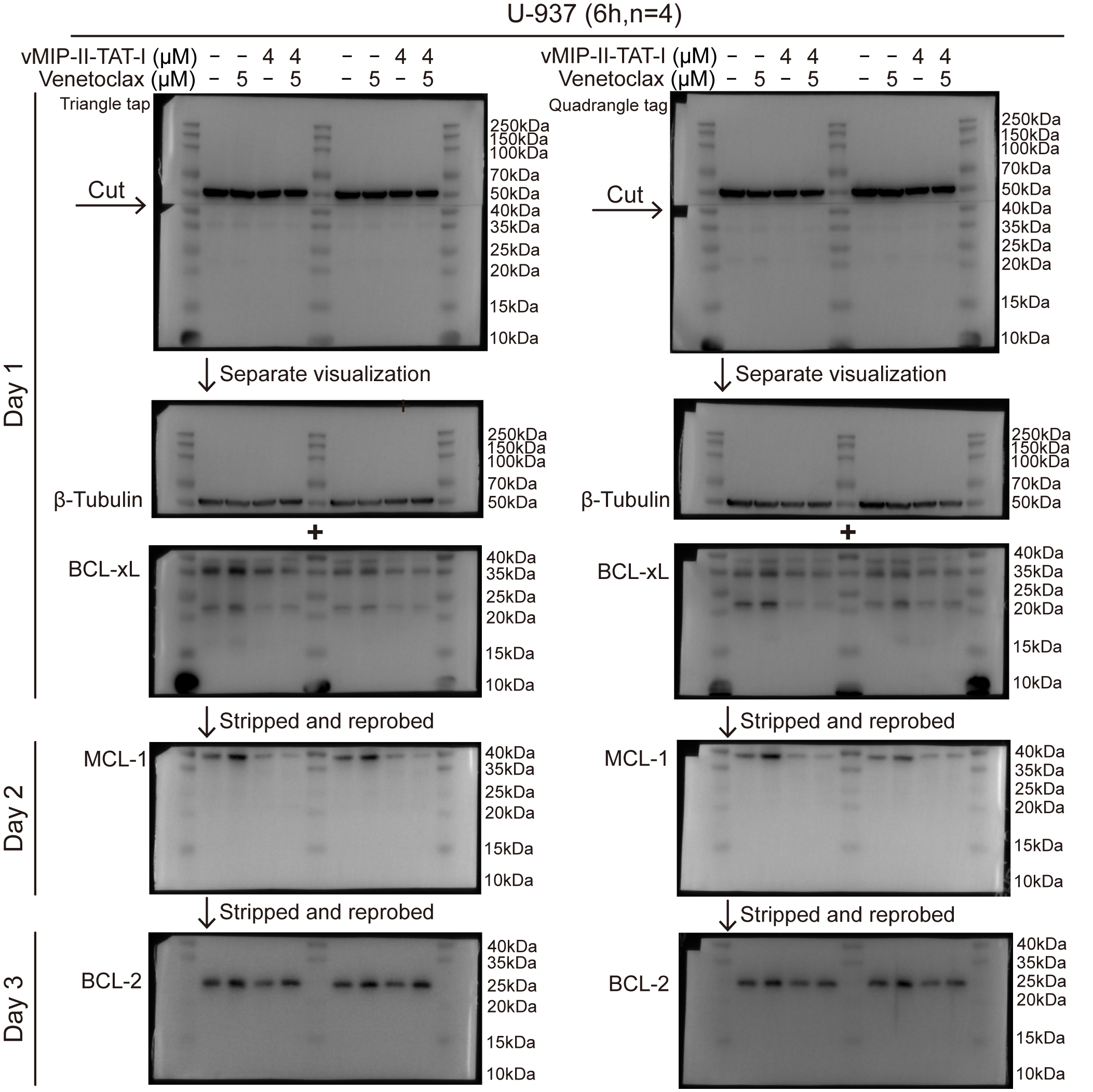

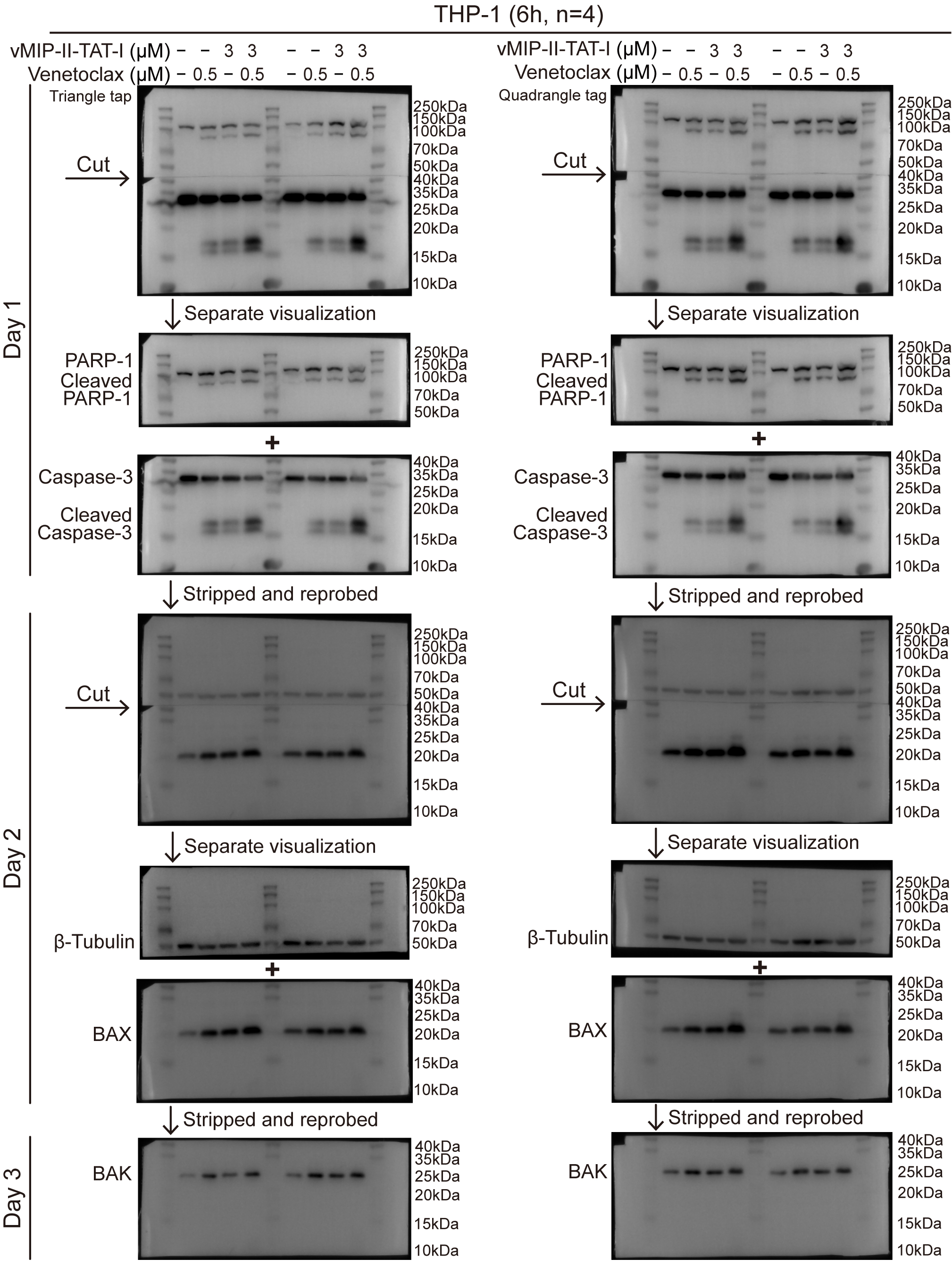

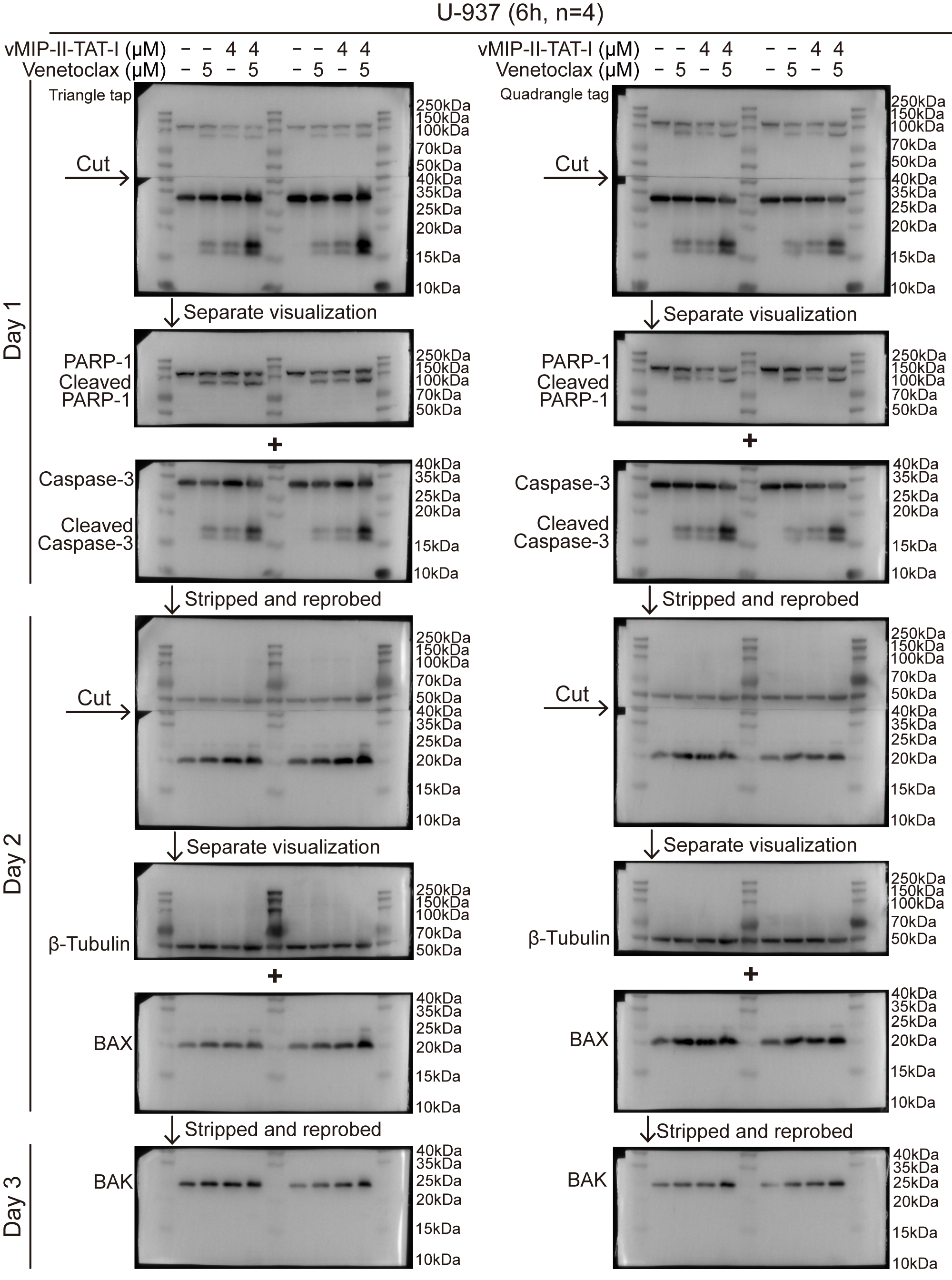


**Figure S11. Unprocessed original scans of western blot analysis.** The PVDF membrane was divided into two sections, and the whole membrane and the segments were exposed and developed each time. Then the antibody binding on the PVDF membrane was washed away with a blot-stripping buffer, and the next antibody incubation test was performed. The same test item was tested with two membranes (n = 4), marked as triangle or quadrilateral membranes, respectively.


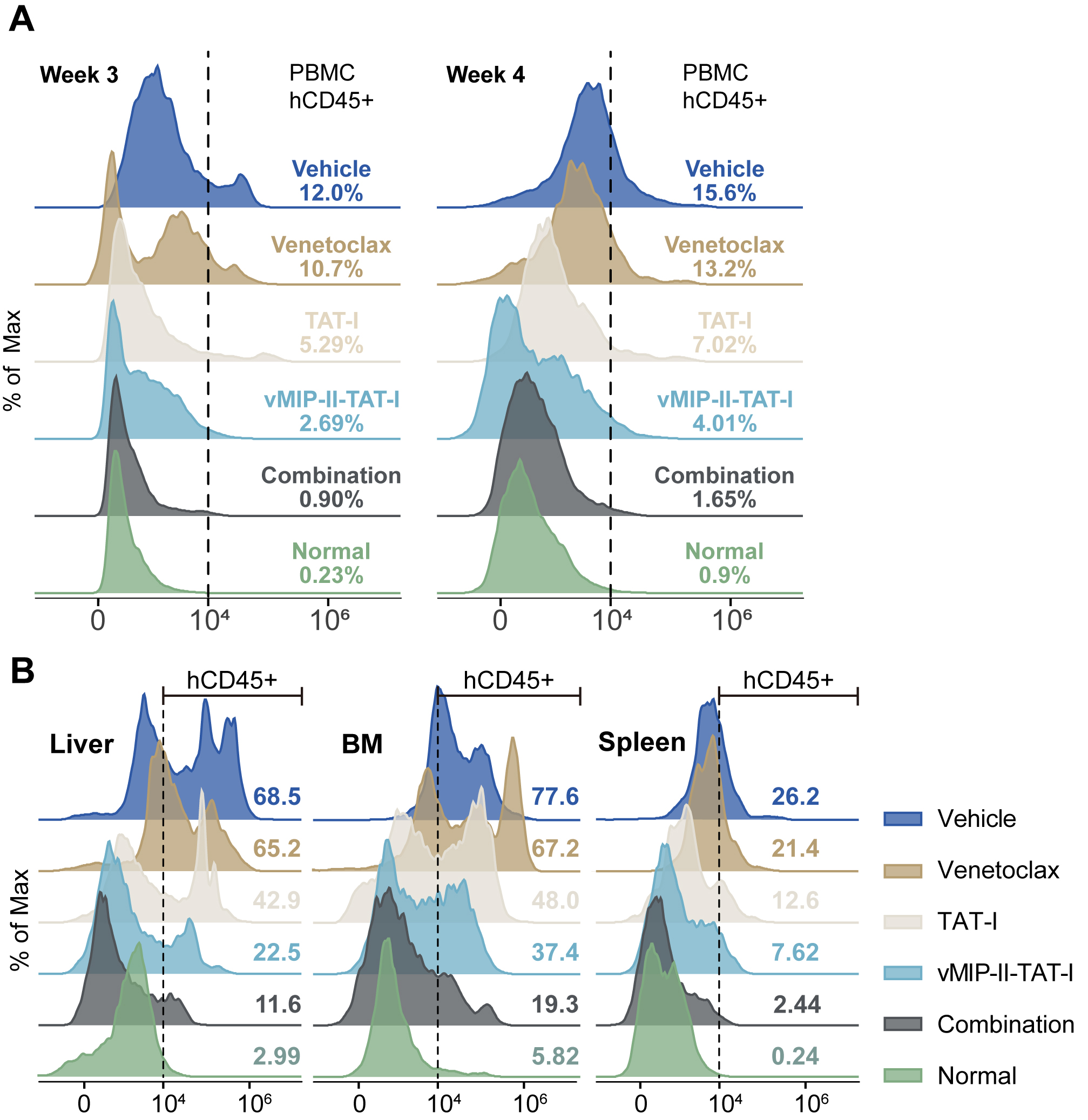


**Figure S12. Leukemia progression in mice monitored by hCD45.** Detection of hCD45+ expression in peripheral blood, liver, bone marrow, and spleen of mice in various experimental groups. The negative range (signal value < 10^4^) is defined as the low/no expression boundary in the corresponding organs and tissues of normal mice.

| Abbreviation | Name | CAS NO. | IUPAC Name | Structure |
| --- | --- | --- | --- | --- |
| Omt | L-Methionine Sulfone | [7314-32-1](https://www.ncbi.nlm.nih.gov/pcsubstance/?term=) | (2S)-2-amino-4-methylsulfonylbutanoic acid |  |
| Chg | L-Alpha-Cyclohexylglycine | 14328-51-9 | (2S)-2-amino-2-cyclohexylacetic acid |  |
| Phe(4-Me), MeF | L-4-Methylphenylalanine | 1991-87-3 | (2S)-2-amino-3-(4-methylphenyl)propanoic acid |  |
| Bpa | L-4-Benzoylphenylalanine | 104504-45-2 | (2S)-2-amino-3-(4-benzoylphenyl)propanoic acid |  |

**Table S1. The identified four favorable ncAAs mutations.**


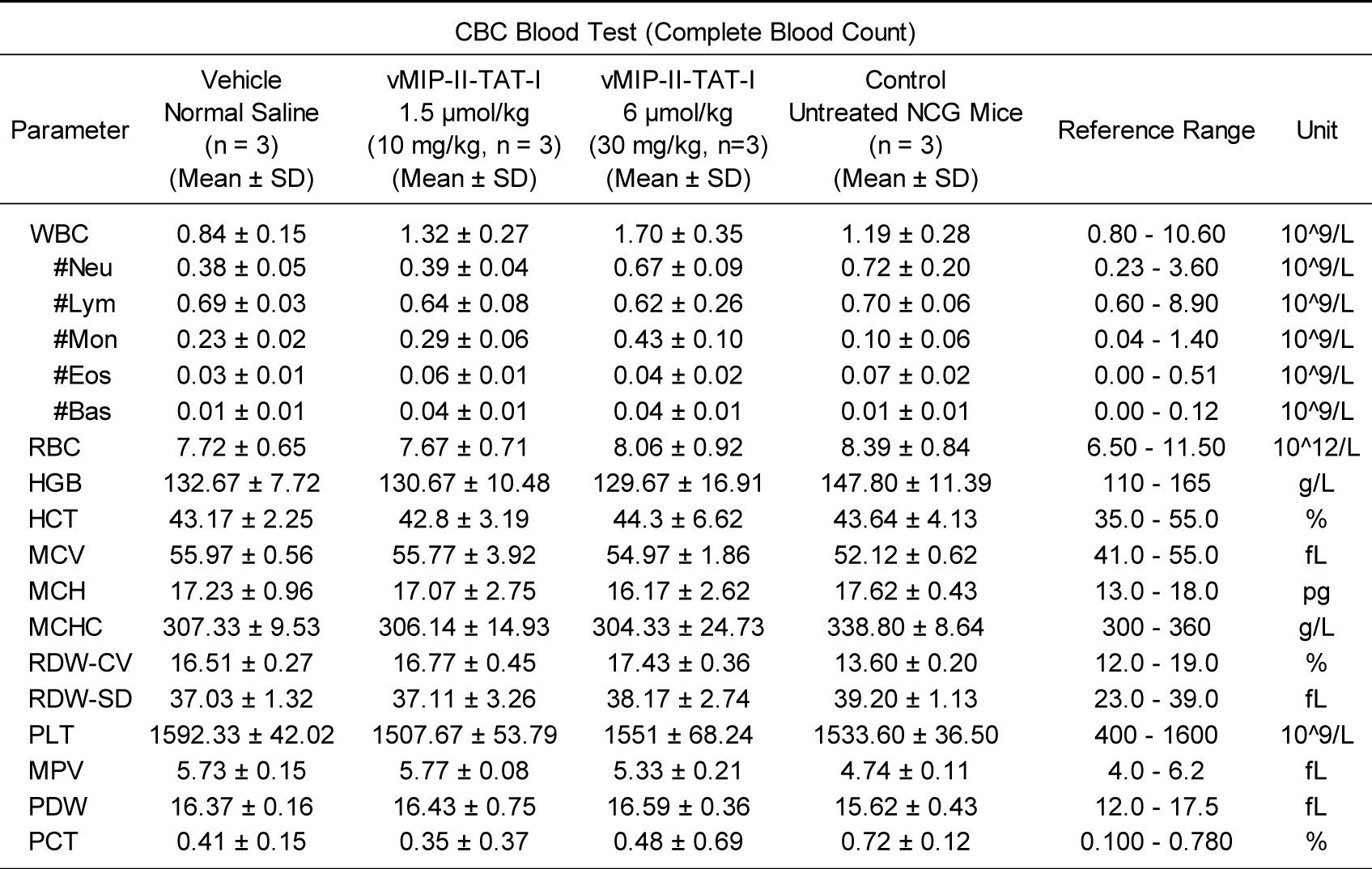


**Table S2. Complete blood counts results.** The results of a complete blood count (CBC) test were conducted to assess the safety profile of vMIP-II-TAT-I at different dosages in NCG mice (n = 3). The parameters measured include white blood cells (WBC), neutrophils (#Neu), lymphocytes (#Lym), monocytes (#Mon), eosinophils (#Eos), basophils (#Bas), red blood cells (RBC), hemoglobin (HGB), hematocrit (HCT), mean corpuscular volume (MCV), mean corpuscular hemoglobin (MCH), mean corpuscular hemoglobin concentration (MCHC), red cell distribution width-coefficient of variation (RDW-CV), red cell distribution width-standard deviation (RDW-SD), platelets (PLT), mean platelet volume (MPV), platelet distribution width (PDW), and plateletcrit (PCT). Data are presented as mean ± SD.
